# Identification of a key peptide cyclase for novel cyclic peptide discovery in *Pseudostellaria heterophylla*

**DOI:** 10.1016/j.xplc.2025.101315

**Published:** 2025-03-13

**Authors:** Xianjin Qin, Fengjiao Wang, Dejin Xie, Qi Zhou, Sheng Lin, Wenxiong Lin, Wei Li

**Affiliations:** 1 Shenzhen Branch, Guangdong Laboratory of Lingnan Modern Agriculture, Key Laboratory of Synthetic Biology, Ministry of Agriculture and Rural Affairs, Agricultural Genomics Institute at Shenzhen, Chinese Academy of Agricultural Sciences, Shenzhen 518000, China; 2 Fujian Provincial Key Laboratory of Agroecological Processing and Safety Monitoring, College of Juncao Science and Ecology, Fujian Agriculture and Forestry University, Fuzhou 350002, China; 3 Bama Yao Autonomous County Rural Revitalization Research Institute, Bama 547500, China

**Keywords:** orbitides, tailoring gene, RiPPs, VIGS, synthetic biology, *Pseudostellaria heterophylla*, heterologous expression

## Abstract

Orbitides, also known as Caryophyllaceae-type cyclic peptides, from the Traditional Chinese Medicine plant *Pseudostellaria heterophylla* (Miq.) Pax, exhibit great potential for improving memory and treating diabetes. Orbitides are ribosomally encoded and post-translationally modified peptides; however, the key biosynthetic enzyme mediating this process remains unknown in *P. heterophylla*. In this study, we investigated the distribution of orbitides in *P. heterophylla* and mined novel precursor peptide genes and peptide cyclases from multiple omics datasets. The function of *PhPCY3*, a gene encoding a key tailoring enzyme, was elucidated using transient heterologous expression and virus-induced gene silencing systems. Our findings suggest that PhPCY3 specifically cyclizes linear precursor peptides *in planta*. Molecular docking and multiple sequence alignment, followed by site-directed mutagenesis, identified N500 and S502 as critical amino acid residues for PhPCY3 function. We identified gene sequences for over 100 precursor peptides and successfully biosynthesized known active orbitides, such as heterophyllin B and pseudostellarin E/F/G. Additionally, four novel orbitides, cyclo-[LDGPPPYF], cyclo-[WGSSTPHT], cyclo-[GLPIGAPWG], and cyclo-[FGDVGPVI], were synthesized using a heterologous expression platform. This study introduces a gene-guided approach for elucidating the biosynthesis pathway and discovering novel orbitides, providing a strategy for mining and biosynthesizing novel orbitides in *P*. *heterophylla* and other plants to further investigate their activities.

## Teaser

Orbitides are bioactive cyclopeptides found in the medicinal plant *Pseudostellaria heterophylla*, but their discovery has been challenging due to their low content. This study reports the identification of the key cyclase PhPCY3 through transient expression and VIGS, enabling the biosynthesis of four novel orbitides using a chassis platform. This reverse genetic strategy overcomes the natural scarcity of orbitides and facilitates the discovery of these valuable compounds.

## Introduction

*Pseudostellaria heterophylla* (Miq.) Pax (family Caryophyllaceae) is utilized in Chinese herbal medicine and possess significant pharmacodynamic value (Hua et al., 2016; Wu et al., 2019; Yang et al., 2020; Xiao et al., 2022). Its roots are traditionally used to treat spleen deficiency, anorexia, post-illness weakness, and spontaneous perspiration (Qin et al., 2017). It is the main component of several commercial products, including indigestion tablets (Ma et al., 2023) and liver rehabilitation pills, generating hundreds of millions of dollars in economic value.

Specifically, heterophyllin B (HB), extracted from the roots of cultivated *P. heterophylla*, is a key quality control indicator required by the Chinese Pharmacopoeia (Commission, 2020). This compound enhances cognitive function through neurite outgrowth and synaptic plasticity and alleviates amyloid-β-induced memory deficits (Yang et al., 2021; Deng et al., 2022). Moreover, HB and its derivatives act as dipeptidyl peptidase IV inhibitors and GLP-1 receptor agonists which have been considered therapeutic alternatives for treating type 2 diabetes (Liao and Tzen, 2022a, 2022b).

Cyclic peptide drugs exhibit valuable pharmacological characteristics, including stable conformation, strong affinity, high metabolic stability, and high oral bioavailability (Muttenthaler et al., 2021; Li et al., 2022; Zhang and Chen, 2022). Drugs based on cyclic peptides, including those with anticancer and antibiotic properties and those used to treat obesity, such as lanreotide and setmelanotide, generate billions of dollars in global sales (Caplin et al., 2014; Ryan, 2020; Zhang and Chen, 2022).

Plant cyclopeptides (CPs) are ribosomally synthesized and post-translationally modified peptides (RiPPs) containing 4–37 amino acids and exhibiting diverse chemical structures (Tan and Zhou, 2006; de Veer et al., 2019; Daly and Wilson, 2021; Chekan et al., 2024). A distinctive characteristic of plant CPs is their ability to undergo macrocyclization, which is typically categorized into three forms: head-to-tail, side-chain-to-side-chain, and side-chain-to-backbone (Kersten and Weng, 2018; de Veer et al., 2019; Slazak et al., 2020; Montalbán-López et al., 2021; Chekan et al., 2024; Kandy et al., 2025). Head-to-tail cyclized CPs, including orbitides and cyclotides, exhibit diverse sequences and bioactivities (Tan and Zhou, 2006; Montalbán-López et al., 2021).

Orbitides containing 5–16 residues and lacking disulfide bonds or non-natural amino acids were originally referred to as Caryophyllaceae-type CPs (Fisher et al., 2019, 2020; Daly and Wilson, 2021) and have demonstrated potential anticancer or antitumor properties (Daly and Wilson, 2021; Tehrani et al., 2021). Evolidine (cyclo-[SFLPVNL]), an orbitide, was the first isolated plant cyclic peptide, and its sequence was confirmed by Fisher et al. (2020). An increasing number of orbitides have been discovered in *P*. *heterophylla*, *Vaccaria segetalis*, and *Stellaria dichotoma* (Morita et al., 1994; Tan and Zhou, 2006; Zhao et al., 2020; Dahiya et al., 2021). Several orbitides, including heterophyllin A–H/J and pseudostellarin A–H/K, have been isolated and purified using traditional phytochemical methods (Tan and Zhou, 2006; Zhao et al., 2020); however, it is unclear whether additional low-abundance orbitides remain undiscovered in *P. heterophylla* using traditional methods.

The precursor peptide genes of CPs are translated by the ribosome as linear peptides and then modified by tailoring and cyclase enzymes (Condie et al., 2011; Barber et al., 2013; Craik and Malik, 2013; Chekan et al., 2017; Montalbán-López et al., 2021). However, the enzymes involved in the biosynthesis of plant cyclic peptides are not conserved across different CPs and do not belong to the same protein family (Chekan et al., 2017; Daly and Wilson, 2021; Kersten et al., 2022). Asparaginyl endoprotease (AEP), a cysteine protease, cleaves Asn or Asp-Xaa bonds to generate an acyl-enzyme intermediate and cyclizes the intermediate into SFTI-like CPs (Bernath-Levin et al., 2015; James et al., 2018; Hemu et al., 2019). Crude oligopeptidase 1 (OLP1) cleaves the linear peptide at the N-terminus to produce a linear intermediate (presegetalin A1[14-32]), which contains an N-terminal glycine (Barber et al., 2013; Chekan et al., 2017). Subsequently, peptide cyclase 1 (PCY1), a serine protease of the S9 family, removes the C-terminal flanking sequence and cyclizes core peptide residues, forming segetalin A (Barber et al., 2013; Chekan et al., 2017). Heterophyllin B, encoded by the *PhPreHB* gene, can be synthesized with crude enzymes in phloem (Zheng et al., 2019), but the role of OLP1 and PCY1 in cyclization is still unclear.

Here, we employed multiple omics datasets to target precursor peptide and tailoring genes. More than 100 precursor peptide genes were identified in the transcriptome, and *PCY1* and *OLP1* were discovered in the genome and transcriptome through Pearson correlation analysis. The key tailoring gene for orbitide formation, *PhPCY3,* was verified using *Nicotiana benthamiana* (*N. benthamiana)* transient expression and *in vivo* virus-induced gene silencing (VIGS) system, and the functional roles of its key active site residues were subsequently examined. We also used this transient expression platform to identify and synthesize new orbitides from the transcriptome. This research not only facilitates the discovery of novel orbitides from the plant kingdom but also provides potential targets for CP drugs.

## Results

### Tissue-specific orbitide accumulation in *P. heterophylla*

**Figure 1:**
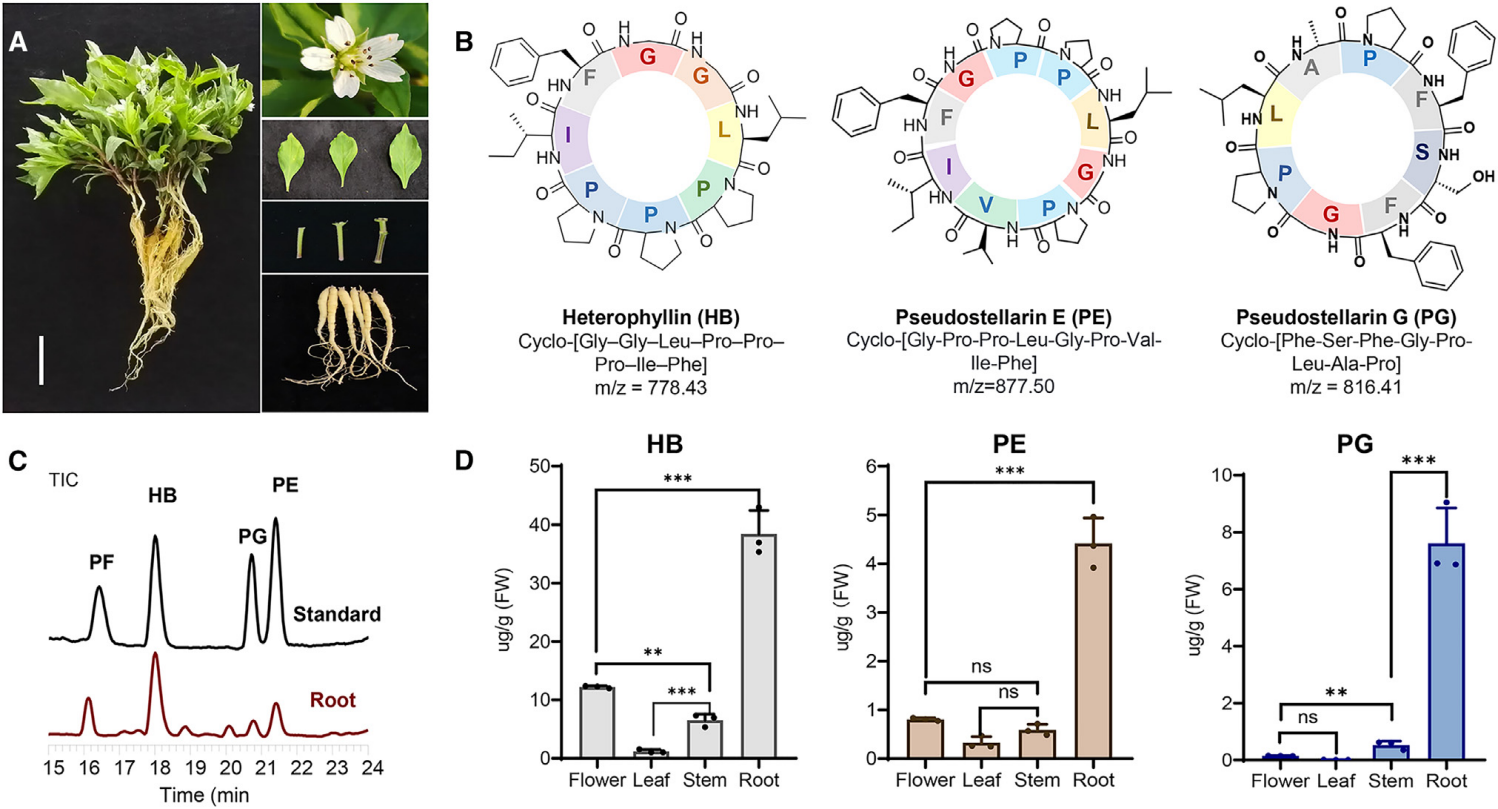
Tissue-specific orbitide accumulation in *P. heterophylla*. (A) Whole plant and tissues sampled from *P. heterophylla in this study.* The scale bar represents 2 cm. (B) Chemical structure of heterophyllin B and pseudostellarin E/G. (C) Total ion chromatogram (TIC) of root extracts from the main cultivar "Zheseng No.2" from Fujian province, showing the known cyclopeptides, including heterophyllin B and pseudostellarin E/F/G, based on Q Exactive HFX LC-MS/MS. (D) Quantified contents of heterophyllin B and pseudostellarin E/G in different tissues using LC-MS (TSQ Quantum Access MAX). The least significant difference method was used for statistical analysis after one-way ANOVA in (completely randomized design) in DPS software (*n* = 3). ∗∗*p* < 0.01, ∗∗∗*p* < 0.001, and “ns” refers to no statistical difference.

To determine orbitide accumulation patterns, we collected different *P. heterophylla* tissues (Figure 1A) and analyzed their orbitide content using liquid chromatography-mass spectrometry (LC-MS) (Figure 1B and 1C; Supplemental Figure 1). Total ion chromatograms (TICs) and extracted ion chromatograms (EICs) generated using a Q Exactive™ HFX Mass Spectrometer showed that HB and pseudostellarin E/F/G (PE/PF/PG) were present in the roots (Figure 1C; Supplemental Figure 1). The particle fragment information was confirmed through secondary mass spectrometry compared to the standards (Supplemental Figure 2). Subsequently, LC-MS (TSQ Quantum Access MAX) was used to quantify orbitide content across different tissues. HB and PE/PG contents were significantly higher in roots than in other tissues (Figure 1D). Additionally, HB content in roots reached approximately 38.4 μg/g (fresh weight [FW]), which was several times higher than PE (4.4 μg/g FW) and PF (7.6 μg/g FW) (Figure 1D).

### Potential tailoring genes in the orbitide biosynthesis pathway

**Figure 2:**
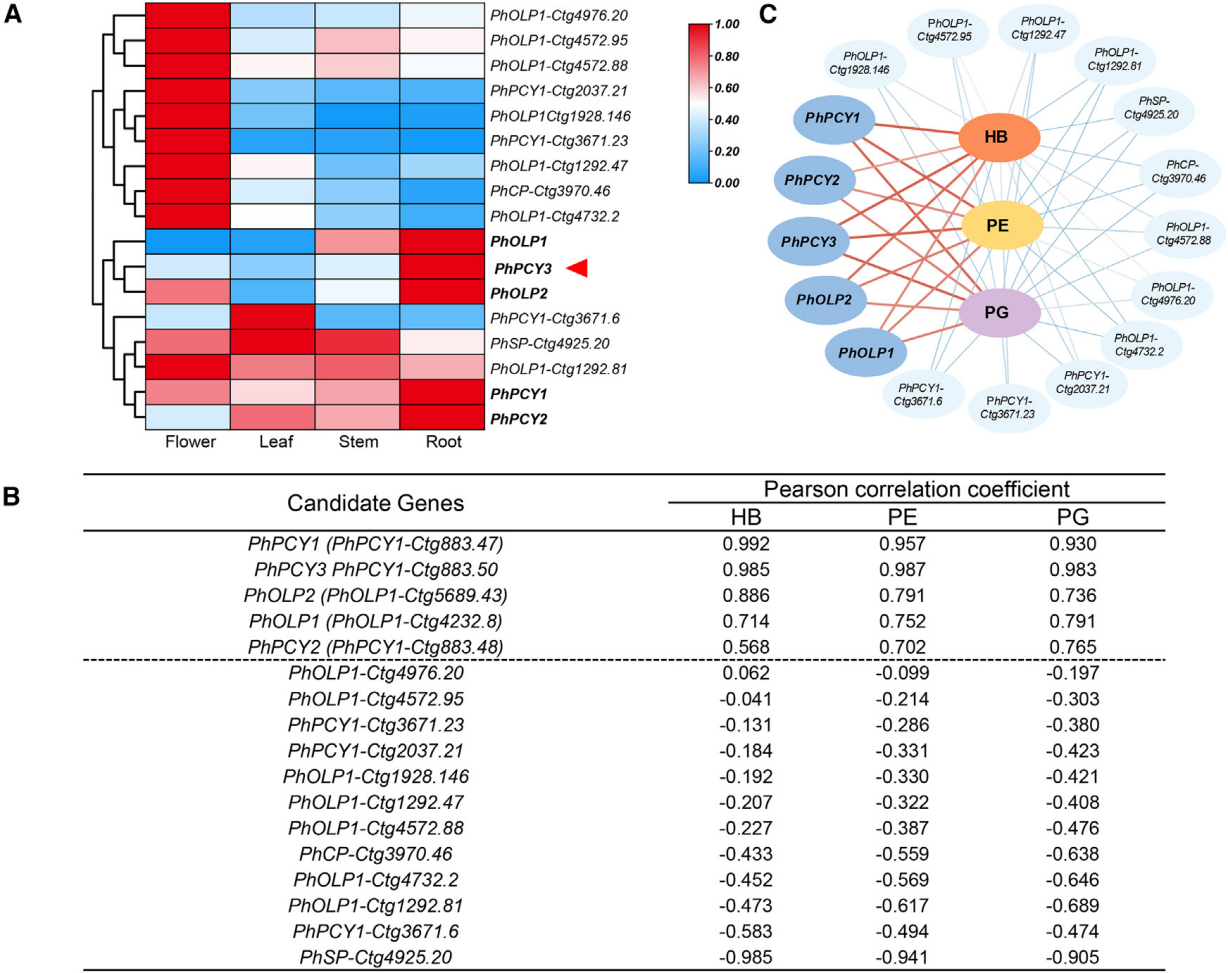
Potential key tailoring genes identified in *P. heterophylla*. (A) Tissue-specific expression of the candidate genes in sampled tissues (flower, leaf, stem, and root). (B) Pearson correlation coefficient between cyclic peptide content and candidate gene expression. (C) Visual network interaction diagram between orbitides and candidate genes. The brown line indicates a Pearson correlation coefficient greater than 0.50.

Cyclic peptides are thought to be synthesized from precursor peptide genes that encode linear precursor peptide sequences (Supplemental Figure 3). OLP1 and PCY1 may be involved in excising and cyclizing linear precursor peptides (Supplemental Figure 3). To analyze the gene expression patterns of candidate genes, RNA-sequencing was performed on the same tissues that were used to detect orbitides. *PhPCY1/2/3* and *PhOLP1/2* genes were highly expressed in roots (Figure 2A), whereas other homologs were primarily expressed in flowers or leaves (Figure 2A). Correlation analysis between cyclic peptide content and candidate gene expression showed that these five genes exhibited a strong positive correlation (Figure 2B and 2C), with *PhPCY1*-*Ctg883.50* (*PhPCY3*) and *PhPCY1*-*Ctg883.47 (PhPCY1)* showing Pearson correlation coefficients greater than 0.90 (Figure 2B).

Phylogenetic analysis of *PhPCY* genes and other known cyclase genes involved in cyclic peptide biosynthesis was performed. Specifically, *SvPCY1* could excise the C -terminus of presegetalin A1[14-32] and cyclize the N -terminus, resulting in the production of segetalin A (Chekan et al., 2017). The results showed that *PhPCY3/2/1* clustered with *SvPCY1*, the first cloned plant cyclase with a confirmed role in peptide cyclization (Barber et al., 2013; Chekan et al., 2017). *PhPCY3* was more closely related to *SvPCY1* than to other homologs (Supplemental Figure 4). In contrast, *PhPCY4*, *PhPCY5*, and *PhPCY6* formed a cluster with *BvPOP*, which encodes a serine protease belonging to the prolyl oligopeptidase subfamily. These results suggest *PhPCY3* is a strong candidate gene in the orbitide biosynthesis pathway.

### PhPCY3 dominates orbitide cyclization

The *N. benthamiana* transient expression platform has been widely used to elucidate biosynthesis pathways (Kersten and Weng, 2018; Xu et al., 2019; Chigumba et al., 2022; De La Peña et al., 2023; Reed et al., 2023; Zhang et al., 2023; Jiang et al., 2024; Zhou et al., 2024). To identify enzymes responsible for CP cyclization, we constructed transient expression vectors for precursor peptide (*PhPreCPs*), *PhPCY1*, and *PhOLP1* genes in pEAQ-HT using homologous recombination (Supplemental Table 1). To narrow down the target genes, these genes were divided into three modules based on their potential functions (Table 1). A wide variety of *PhPreCPs*, which are essential substrates for orbitide synthesis, were placed in module 1. Modules 2 and 3 contained *PhOLP* and *PhPCY* genes, respectively (Table 1).

**Table 1:** Modules of candidate genes in different groups.

Group name	Module 1 (PreCPs)	Module 2 (PhOLPs)	Module 3 (PhPCYs)
pEAQ-HT (negative control)	−	−	−
PhPreHB	+	−	−
PhPreHB + PhOLPs	+	+	−
PhPreHB + PhPCYs	+	−	+
PhPreHB + PhOLPs + PhPCYs	+	+	+

PreCP refers to PreHB. + (−) refers to with (without) the candidate genes in this module. PhOLPs contain *PhOLP1/2*; PhPCYs include *PhPCY1/2/3*.

**Figure 3:**
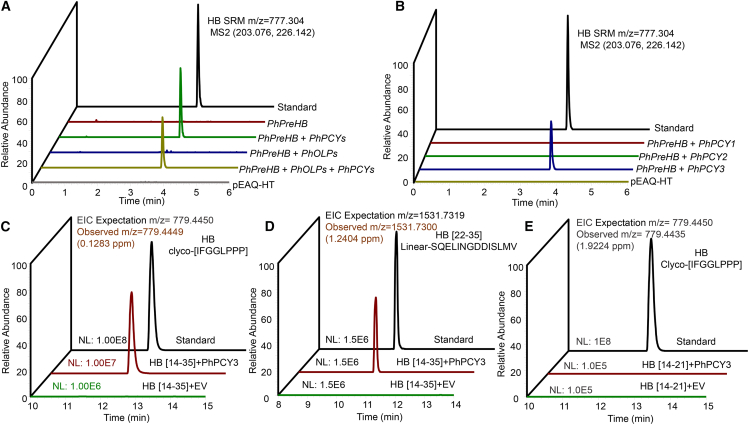
PhPCY3 dominates HB biosynthesis *in vivo* and *in vitro*. (A) PhPCYs containing core genes in the heterophyllin B biosynthesis pathway. The *Nicotiana benthamiana* heterologous expression platform was used to verify the functions of candidate genes. All candidate genes were divided into three modules. Module 1 is the precursor protein gene *PhPreHB* necessary for the pathway. Module 2 includes two OLP genes encoding enzymes that may excise precursor peptides at the N-terminus. Module 3 contains three *PCY* genes encoding enzymes that may break intermediate products at the C-terminus and cyclize to form orbitides. *N. benthamiana* leaf samples were detected using LC-MS in the selective reaction monitoring (SRM) mode. (B) Co-expressing *PhPreHB* and *PhPCY3* in *N. benthamiana* leaves can cyclize PreHB to produce HB. (C and D) *In vitro* activity analysis of PhPCY3 with HB [14-35] as the substrate. PhPCY3 was expressed in the pMAL-c5x vector with an MBP tag and the purified protein was used to elucidate the catalytic mechanism *in vitro*. Extracted ion chromatograms of HB (dark red, observed m/z = 779.4449, z = 1, 0.1283 ppm) are shown with the standard (C). Extracted ion chromatograms of HB [22-35] (dark red, observed m/z = 1531.7300, z = 1, 1.2402 ppm) are shown with the standard. HB [22-35] refers to a linear peptide with the sequence SQELINGDDISLMV (D). (E) *In vitro* activity of PhPCY3 with HB [14-21] as the substrate. Extracted ion chromatograms of HB (black, m/z = 779.4435, z = 1, 1.9224 ppm) are shown with the standard.

Different modules were co-infiltrated into *N*. *benthamiana* leaves with precursor peptide genes. HB was detected using two daughter ion fragments (m/z = 203.035 and 226.129) in selective reaction monitoring (SRM) mode. The *PhPCY* module included the core enzyme(s) responsible for the HB biosynthetic pathway (Figure 3A; Supplemental Figure 5A, 5C, and 5D). The minor peak detected in the PhOLPs module was not attributable to HB, as evidenced by the distinct MS2 fragment profiles (Figure 3A; Supplemental Figure 5B). Furthermore, chromatogram results showed that among PhPCYs, only PhPCY3 catalyzed the linear precursor peptide to HB, and it could function without the help of PhOLPs (Figure 3B; Supplemental Figure 6). Given that *PhOLP1* and *PhOLP2* had a strong positive correlation with orbitide accumulation and were specifically highly expressed in the roots (Figure 2B and 2C), we expressed *PhOLP1/2* in *N. benthamiana* leaves to confirm their activities. Neither could function independently in orbitide biosynthesis (Supplemental Figure 6). *PhPCY3* also cyclized PrePE, PrePF, and PrePG to PE, PF, and PG, respectively (Supplemental Figure 7). These results are highly consistent with the tissue-specific expression and evolutionary relationships of the *PhPCY3* gene (Figure 2B and 2C). Furthermore, an *in vitro* enzymatic activity assay was performed on PhPCY3 using two distinct substrates: HB [14-35], a linear peptide encompassing the IFGGLPPPSQELINGDDISLMV sequence, and HB [14-21], a truncated variant with the IFGGLPPP sequence. PhPCY3 excised the C-terminal region of HB [14-35] and cyclized the propeptide to form HB (Figure 3C and 3D; Supplemental Figures 8 and 9A). Additionally, the linear peptide HB [22-35] (SQELINGDDISLMV) was detected as a byproduct (Figure 3D; Supplemental Figure 9B). However, PhPCY3 was unable to directly catalyze the conversion of the shorter substrates, HB [14-21], into HB (Figure 3E). These results suggest that PhPCY3 is the primary enzyme for orbitide biosynthesis.

### Verification of PhPCY3 activity via VIGS in *P. heterophylla*

**Figure 4:**
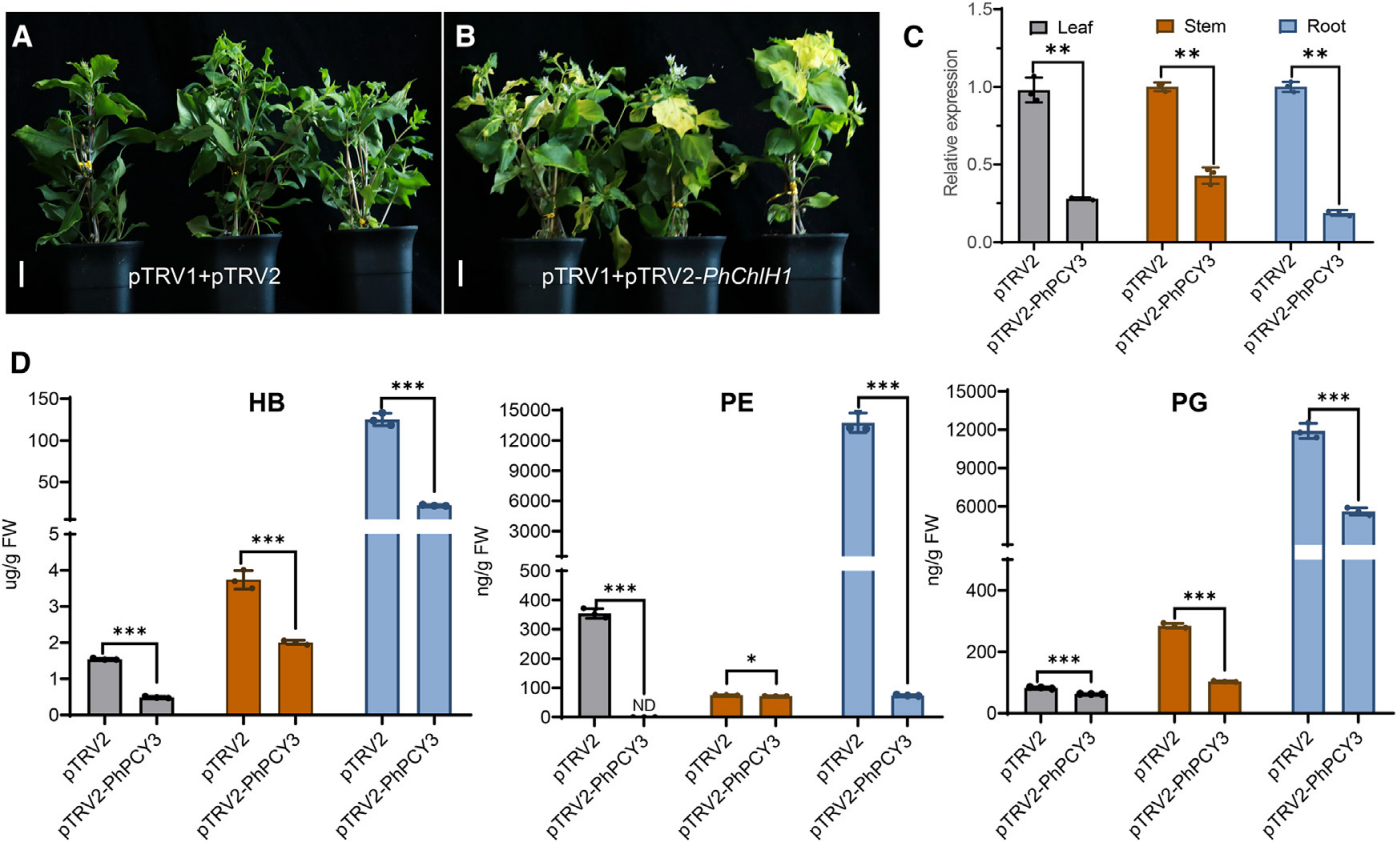
Silencing of the *PhPCY3* gene using the VIGS system. (A) The negative control group was infiltrated with mixed strains containing pTRV1 and pTRV2 empty plasmids separately. The scale bar represents 2 cm. (B) The positive control group was infiltrated with a mixture of strains containing pTRV1 and pTRV2-*PhChlH1* plasmids, showing a yellow leaf phenotype after 4 weeks of treatment. The scale bar represents 2 cm. (C) Relative expression of the *PhPCY3* gene in different tissues in the pTRV2-PhPCY3 group compared to the negative control group. (D) HB, PE, and PG contents in different tissues in the pTRV2-PhPCY3 group and the negative control group. The least significant difference method was used for statistical analysis after one-way ANOVA (completely randomized design) in DPS software (*n* = 3). ∗*p* < 0.05, ∗∗*p* < 0.01, ∗∗∗*p* < 0.001, ND = not detected.

To further investigate the function of *PhPCY3 in planta*, a VIGS platform was employed. *ChlH* encodes the H subunit of magnesium protoporphyrin chelatase, an enzyme that plays a crucial role in chlorophyll biosynthesis. Silencing *ChlH* results in a yellow leaf phenotype in both *N. benthamiana* and *Gossypium arboreum* (Hiriart et al., 2002; Fan et al., 2023), making this gene a suitable control for our VIGS experiments. We silenced the *PhChlH1* gene in *P. heterophylla* using *Agrobacterium*-mediated transformation, resulting in a yellow leaf phenotype for about 4 weeks (Figure 4A and 4B; Supplemental Table 2).

Subsequently, we interfered with *PhPCY3* expression in the leaves and collected tissue samples to determine the expression of the target genes using quantitative real-time PCR (qRT-PCR) (Supplemental Table 3) and orbitide content using LC-MS. Quantitative real-time PCR results showed that *PhPCY3* was significantly downregulated compared with the negative control group (Figure 4C). Moreover, the HB, PE, and PG contents in these tissues decreased significantly when *PhPCY3* was silenced (Figure 4D). *PhPreHB*, *PhPrePE*, and *PhPrePG* were downregulated in leaves when these genes were silenced (Supplemental Figure 10A) which corresponded to the significantly decreased HB, PE, and PG levels (Supplemental Figure 10B). These results indicate that *PhPCY3* is essential for orbitide cyclization *in planta* and that *PreCPs* encode the necessary substrates for orbitide biosynthesis.

### Key catalytic sites affect cyclization activity

**Figure 5:**
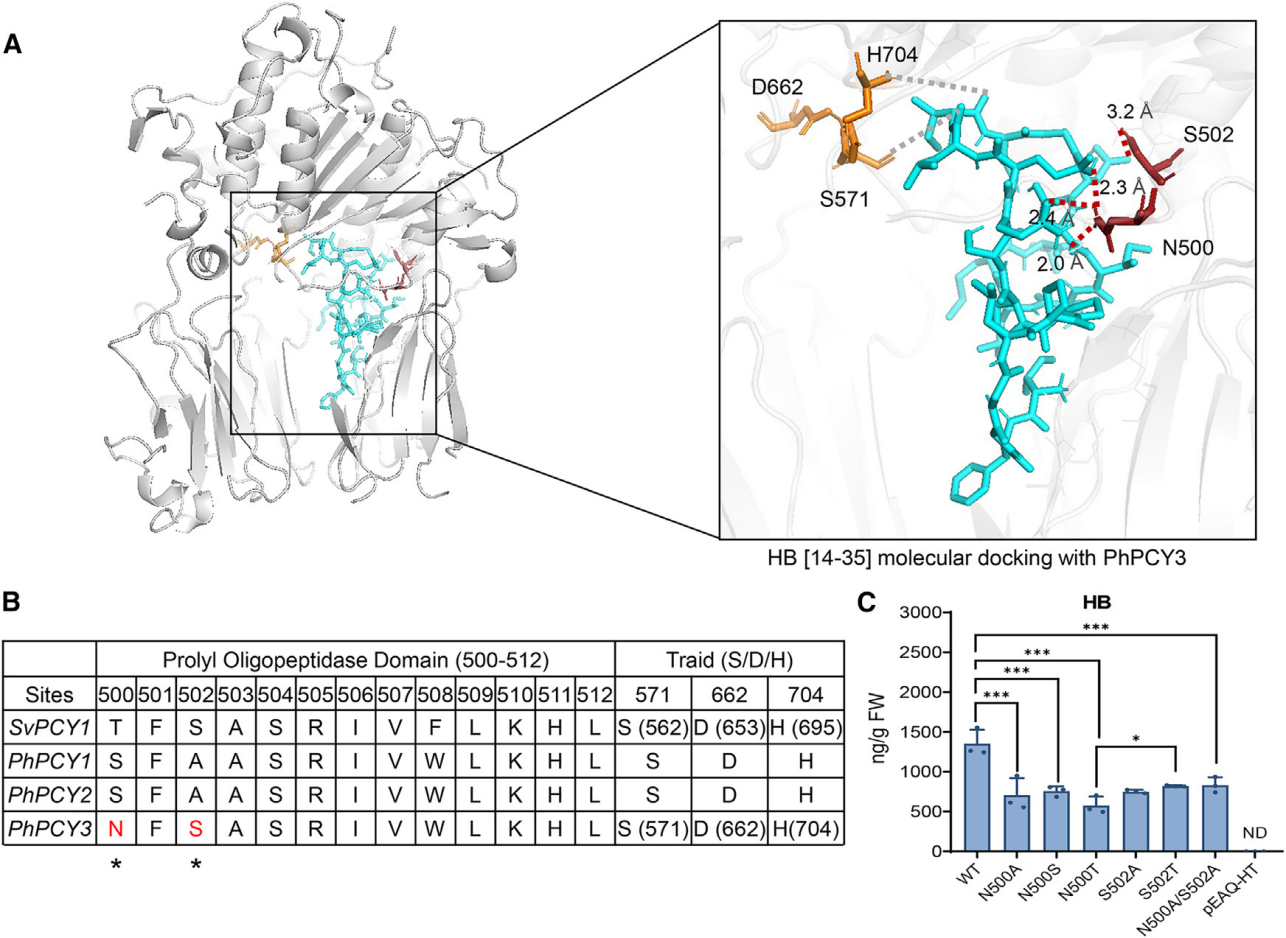
Key catalytic sites identified via molecular docking and multiple sequence alignment. (A) Molecular docking of substrate HB [14-35] (cyan) with PhPCY3, showing hydrogen bonding interactions within 4 Å at the N500 and S502 sites, highlighted by dark red dashed lines. The catalytic triad amino acids (S571, D662 and H704) are shown in the molecular docking, with the residues nearest to the oxygen atom in the substrate highlighted by gray dashed lines. (B) Key catalytic sites (N500 and S502) identified in the prolyl oligopeptidase domain (PF00326) of PCYs. The catalytic triad of amino acids in SvPCY1 and PhPCY3, including S571, D662, and H704, is also shown within the prolyl oligopeptidase domain. (C) Quantitative analysis of HB content in tobacco leaves infiltrated with site-directed mutated PhPCY3 at the N500 and S502 sites by LC-MS in selected reaction monitoring (SRM) mode. The least significant difference method was used for statistical analysis after one-way ANOVA (completely randomized design) in DPS software (*n* = 3). ∗*p* < 0.05 and ∗∗∗*p* < 0.001.

Molecular docking between PhPCY3 and its substrates (PreCPs [14-35/56]) was performed to elucidate the potential cyclization mechanism. The core peptides and the C-terminal follower peptides are known to be substrates catalyzed by peptide cyclase 1 (Chekan et al., 2017). Molecular docking between the substrate PreCPs [14-35/56] and PhPCY3 in 4 Å revealed that amino acid residues in the pocket of PhPCY3 could form hydrogen bonds with the substrate amino acids (Figure 5A; Supplemental Figures 13–15). These catalytic sites include N500, which was identified across different substrates, suggesting its importance for tailoring various cyclic peptides (Supplemental Figure 12). Multiple sequence alignment and amino acid domain analysis of PhPCY3/2/1 and the functionally validated *SvPCY1* gene revealed that N500 and S502 are sites that differ between functional and non-functional PCY1s. Notably, N500 is the first amino acid residue in the prolyl oligopeptidase domain (Pfam PF00326) of PhPCY3 (Figure 5B; Supplemental Figure 11). These results suggest that N500 and S502 may be key catalytic sites that affect cyclization activities.

To assess the roles of these residues, mutations N500 A/S/T, S502 A/T, and the double mutation N500A with S502A were introduced into PhPCY3 using site-directed mutagenesis PCR (Figure 5B; Supplemental Table 4). The catalytic activity of the mutated enzyme and HB content were measured using the transient *N. benthamiana* expression platform through LC-MS (Figure 5C; Supplemental Figure 16). The HB content decreased significantly when the N500 site was mutated to N500A or N500 S/T compared to the wild type (Figure 5C). Similar results were obtained when the S502 site was mutated to A or T. Moreover, the HB content was significantly reduced in tobacco leaves when both sites were mutated to A (Figure 5C; Supplemental Figure 16).

### Identification of novel orbitides through the transient expression platform

**Figure 6:**
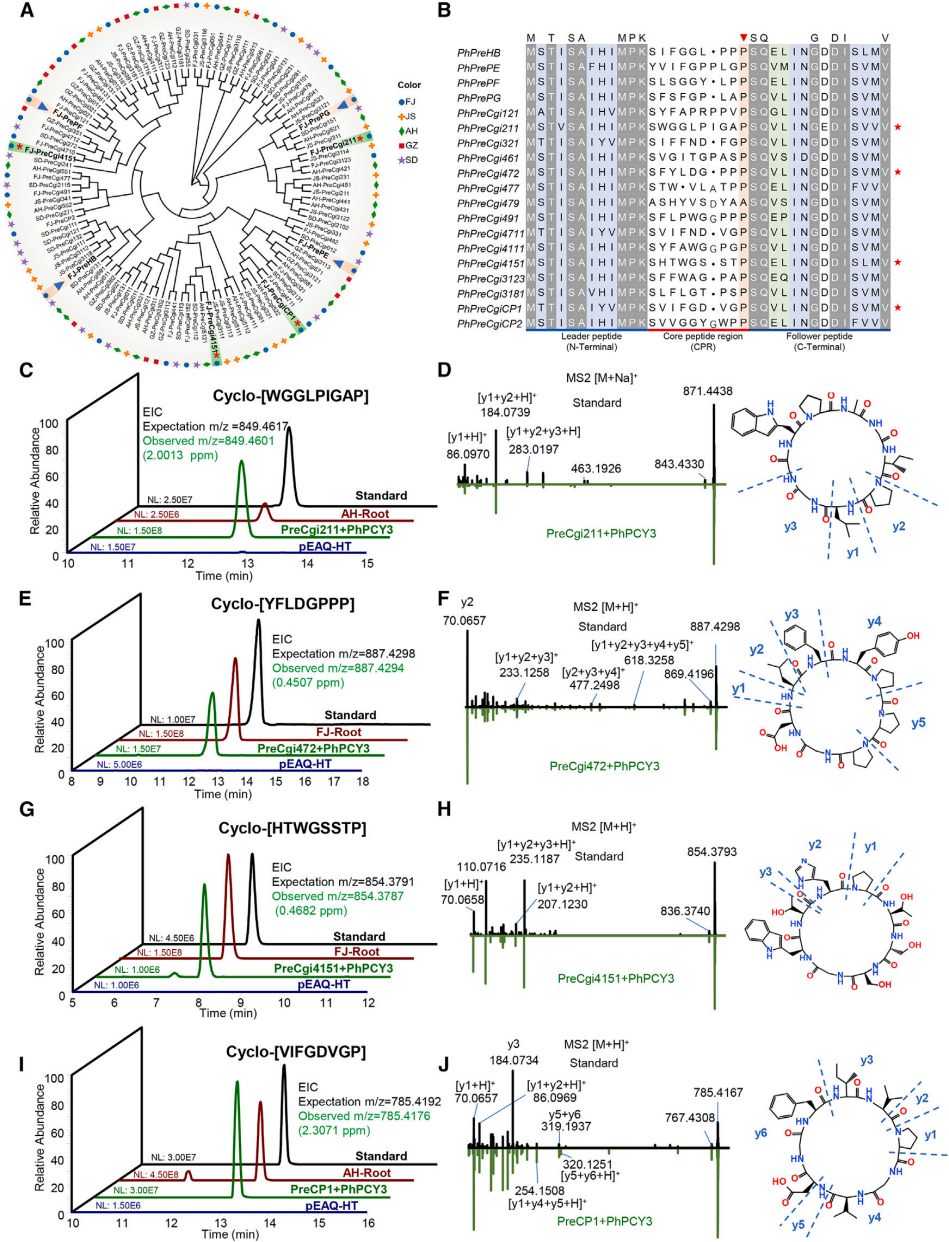
*PreCP*-guided discovery of novel orbitides. (A) Phylogenetic analysis of precursor peptide sequences *in Pseudostellaria heterophylla*. A maximum likelihood tree was constructed using Mega software. Bootstrap values are indicated at the nodes (based on 1000 replicates). FJ, GZ, SD, AH, and JS refer to different *P. heterophylla* cultivars from Fujian, Guizhou, Shandong, Anhui, and Jiangsu provinces, respectively. Blue triangles represent precursor peptide sequences of known orbitides. Red asterisks indicate sequences of selected precursor peptides for novel orbitide discovery. (B) Sequence alignment and conserved structure analysis of precursor peptide sequences in *P. heterophylla* (FJ). Amino acid sequences of the leader peptide (N-terminus) and the follower peptide (C-terminus) are conserved, while the core peptide regions (CPRs) show remarkable diversity. The last amino acid of the core peptide is always Pro or Ala, marked by red triangles. The red star indicates the selected precursor peptide sequence for novel orbitide discovery. (C) Discovery of novel orbitide cyclo-[WGGLPIGAP] by co-expressing *PhPreCgi211* and *PhPCY3* in tobacco leaves. The EIC map of cyclo-[GLPIGAPWG] in the source plant (AH-Root) and tobacco leaves compared with the synthetic standard. The theoretical value (accurate molecular weight) for cyclo-[GLPIGAPWG] was 849.4618. The observed m/z values were: standard sample (black), 849.4598 (2.3544 ppm); source plant sample (dark red), 849.4611 (0.8240 ppm); and tobacco leaf sample (green), 849.4601 (2.0013 ppm). (D) MS2 particle fragment information for novel cyclo-[WGGLPIGAP] biosynthesized in *Nicotiana benthamiana* leaves compared with the synthetic standard. The chemical structure and partial fragmentation patterns of cyclo-[GLPIGAPWG] are illustrated. (E) Discovery of novel orbitide cyclo-[YFLDGPPP] by co-expressing *PhPreCgi472* and *PhPCY3* in tobacco leaves. EIC map of cyclo-[YFLDGPPP] in the source plant (FJ-Root) and tobacco leaves compared with the synthetic standard. The theoretical value (accurate molecular weight) for cyclo-[YFLDGPPP] was 887.4298. The observed m/z values were: standard sample (black), 887.4282 (1.8030 ppm); source plant sample (dark red), 887.4286 (1.3522 ppm); and tobacco leaf sample (green), 887.4294 (0.4057 ppm). (F) MS2 particle fragment information for novel cyclo-[YFLDGPPP] biosynthesized in *N. benthamiana* leaves compared with the synthetic standard. The chemical structure and partial fragmentation patterns of cyclo-[YFLDGPPP] are illustrated. (G) Discovery of novel orbitide cyclo-[HTWGSSTP] by co-expressing *PhPreCgi4151* and *PhPCY3* in tobacco leaves. EIC map of cyclo-[HTWGSSTP] in the source plant (FJ-Root) and tobacco leaves compared with the synthetic standard. The theoretical value (accurate molecular weight) for cyclo-[HTWGSSTP] was 854.3791. The observed m/z values were: standard sample (black), 854.3788 (0.3511 ppm); source plant sample (dark red), 854.3773 (2.1068 ppm); and tobacco leaf sample (green), 854.3787 (0.4682 ppm). (H) MS2 particle fragment information for novel cyclo-[HTWGSSTP] biosynthesized in *N. benthamiana* leaves compared with the synthetic standard. The chemical structure and partial fragmentation patterns of cyclo-[HTWGSSTP] are illustrated. (I) Discovery of novel orbitide cyclo-[VIFGDVGP] by co-expressing *PhPreCP1* and *PhPCY3* in tobacco leaves. EIC map of cyclo-[VIFGDVGP] in the source plant (AH-Root) and tobacco leaves compared with the synthetic standard. The theoretical value (accurate molecular weight) for cyclo-[VIFGDVGP] was 785.4192. The observed m/z values were: standard sample (black), 785.4178 (1.7825 ppm); source plant sample (dark red), 785.4177 (1.9098 ppm); and tobacco leaf sample (green), 785.4176 (2.3071 ppm). (J) MS2 particle fragment information for novel cyclo-[VIFGDVGP] biosynthesized in *N. benthamiana* leaves compared to the synthetic standard. The chemical structure and partial fragmentation patterns of cyclo-[VIFGDVGP] are illustrated.

Numerous precursor peptide genes of orbitides were identified by mining transcriptome data. These genes not only contained known orbitides but were also abundant with potential novel orbitides (Figure 6A and 6B). Several precursor peptide genes and known orbitides, including *PhPreHB*, *PhPrePE*, *PhPrePF*, and *PhPrePG,* were used to identify key tailoring genes (Figure 3). Analysis of amino acid sequences showed that the leader peptides at the N-terminal and the C-terminal ends of the follower peptide were highly conserved, whereas the core peptides displayed diverse cyclic peptide structures (Figure 6B). Novel orbitides, namely cyclo-[GLPIGAPWG], cyclo-[LDGPPPYF], cyclo-[WGSSTPHT], and cyclo-[FGDVGPVI], were identified using the *PhPCY3* heterologous expression platform. These compounds were characterized through comparison with established standards and the source plant (Figure 6C, 6E, 6G, and 6I). Furthermore, the chemical structures and partial fragmentation patterns of the orbitides were utilized to elucidate the peptide sequences. The ion fragment information from secondary mass spectrometry of the novel orbitides was consistent with the standards (Figure 6D, 6F, 6H, and 6J). In summary, we adopted reverse genetics to screen precursor peptide genes using multiple omics datasets, and a transient expression platform was utilized to identify key tailoring genes and orbitides (Supplemental Figure 17).

## Discussion

Natural products have long been explored through bioactivity-guided approaches. However, in recent years, the explosion of multi-omics data has accelerated the gene-guided discovery of novel natural products (Scherlach and Hertweck, 2021; Chigumba et al., 2022; Kersten et al., 2022; Pei et al., 2023; Mydy et al., 2024). Notably, non-squalene triterpenes have been identified by mining fungal chimeric class I triterpene synthases using a yeast-based genome mining platform (Chen et al., 2021; Tao et al., 2022). Cyclic peptides, with their diverse chemical structures and biological activities, are widespread throughout the plant kingdom and are well-suited for discovery via gene-guided approaches. For example, Fisher et al. used *de novo* transcriptomics and tandem mass spectrometry to rediscover evolidine and six novel orbitides in *Melicope xanthoxyloides* (Fisher et al., 2020). Furthermore, Song et al. developed sophisticated mining strategies to identify specific sequences within the *Linum usitatissimum* genome and used these data to investigate the genetic diversity of orbitides (Song et al., 2022). These identified sequences encompass multiple core peptide regions (CPRs), which contrasts the single CPR observed in orbitides from *P. heterophylla* and *M. xanthoxyloides*. However, various precursor peptide genes of potential orbitides were identified in the *P. heterophylla* transcriptome in this study, indicating that CPs exist *in planta* that have not yet been isolated.

Orbitides are the second largest identified group of plant cyclic peptides that do not contain disulfide bonds or non-natural amino acids. Numerous precursor peptide sequences have also been identified in several genera within the Rutaceae family by mining RNA-sequencing and whole genome sequencing data (Fisher et al., 2020). These peptides feature diverse residues at the C-terminus of CPRs, such as Phe, Leu, Ser, or Lys, which are not recognized by asparaginyl endopeptidase (Asn or Asp) or prolyl oligopeptidase (Pro or Ala), suggesting that one or more novel tailoring enzymes have yet to be discovered in Rutaceae. CPs discovered in the tuberous roots of *P. heterophylla* were isolated and identified in the 1990s using traditional phytochemical methods (Tan and Zhou, 2006). However, pseudostellarin K, the only orbitide discovered in the fibrous roots of *P. heterophylla*, was identified more recently (Zhao et al., 2020). This indicates that traditional methods may encounter bottlenecks in isolating low abundance natural products.

Multiple omics datasets have been used to explore candidate genes in the orbitide biosynthesis pathway. Many precursor genes were identified from our transcriptome data (Figure 6A). The amino acid sequences of the core peptide showed remarkable diversity compared with the flanking sequences on both sides, but the residues at the C-terminus of the core peptide were always Pro or Ala (Figure 6B), which can be recognized by SvPCY1 (Barber et al., 2013). A serine protease (SvOLP1) cleaves the linear peptide precursor presegetalin A1 [1-32] to presegetalin A1 [14–32], SvPCY1 then excises the intermediate at the C-terminal presegetalin A1 [20–32] and cyclizes the core peptide into segetalin A (Barber et al., 2013; Chekan et al., 2017). Genes encoding these enzymes in the *P. heterophylla* genome are hypothesized to be involved in orbitide biosynthesis pathways (Figure 2; Supplemental Figure 3). PhPCY3 has been demonstrated to participate in orbitide biosynthesis without the help of OLPs using a plant transient expression platform (Figure 3A and 3B). Meanwhile, PhPCY3 excises the HB [14-35] substrate at the C-terminus and cyclizes the propeptide into HB, as shown in an *in vitro* enzyme activity assay. Although the S9 family of proteases is found ubiquitously in nature, those with peptide ligase activity are much rarer (Nguyen et al., 2014). Furthermore, PhPCY3 is the second orbitide cyclase validated outside the original SvPCY1.

Previous studies have shown that some RiPP-modifying enzymes, such as those in thiazole/oxazole–modified microcin (TOMM) biosynthesis, recognize and bind the conserved N-terminal leader of precursor peptides (Burkhart et al., 2015; Koehnke et al., 2015). In addition, aspartate endopeptidases (AEPs) are cysteinyl enzymes that catalyze the hydrolysis and transpeptidation reaction and play a crucial role in the cleavage and cyclization of plant cyclic peptides. Karen et al. identified *OaAEP1*_*b*_ from *Oldenlandia affinis* and found that its encoded enzyme couples the C-terminus cleavage of propeptide substrates with backbone cyclization, however, it cannot cleave the N-terminal site of cyclotide precursors (Harris et al., 2015). Similarly, CeAEP1 contains cleavage-coupled macrocyclization activity at the C-terminus to form SFTI-1 via intramolecular transpeptidation (Bernath-Levin et al., 2015). Interestingly, MCoAEP2 mediates both the N-terminal excision and the C-terminal cyclization of cyclotide precursors (Du et al., 2020). However, SvPCY1, which belongs to the S9 class of the protease family, generates macrocyclic products by catalyzing the hydrolytic cleavage of peptide substrates at the C-terminus (Luo et al., 2014; Chekan et al., 2017). In the present study, hydrogen-bonding interactions were predicted between PhPCY3 residues and the follower peptide of PreCPs [14-35/36] at the C-terminus in 4 Å (Supplemental Figure 12).

The C-terminus follower peptides bind to SvPCY1 with hydrogen bond interactions via the triad amino acids (Ser562, Asp653, and His695), indicating that the triad plays a critical role in the cyclase during segetalin A biosynthesis (Chekan et al., 2017). The molecular basis of recognition and catalysis for SvPCY1 has been revealed, expanding the substrate scope of this enzyme (Ludewig et al., 2018). In the present study, multiple sequence alignment showed that the triad amino acids were conserved in PhPCY1/2/3 and SvPCY1, although PhPCY1/2 had no catalytic activity to produce orbitides. Amino acid sequence comparison between PhPCY3 and PhPCY1/2 and the molecular docking of PhPCY3 showed that N500 and S502 in the prolyl oligopeptidase domain were important for the catalytic activities of PhPCY3 via hydrogen bond interactions. However, our activity assays demonstrated that mutations at these two sites reduced but did not completely abolish catalytic activity. The phenotypic effects of the Ser502 mutation in PhPCY3 were consistent with those observed for the analogous Ser493 mutation in SvPCY1 (Ludewig et al., 2018). The cyclization mechanisms and processes vary among plants. Orbitides in the Caryophyllaceae and Rutaceae families likely involve distinct protease classes, underscoring the need for diverse approaches to uncover novel cyclization mechanisms. While the *PhPCY3* gene has proven effective in discovering orbitides in *P. heterophylla*, further evidence is still needed to confirm whether it can facilitate mining orbitides in other species.

RiPP CPs are a rich source of chemical and structural diversity, and macrocyclic peptides have emerged as excellent targets for drug discovery over the past decade (Passioura et al., 2014; Hosseinzadeh et al., 2017). Disulfide-rich CPs, in particular, have garnered increased attention in drug development, thanks to a yeast-based protocol that enables their biosynthesis via AEP *in vitro* (Yap et al., 2021). Here, we established an approach using the plant transient expression platform to discover and synthesize novel orbitides by mining transcriptome data (Supplemental Figure 17). These novel CPs could be screened for their anti-nematode activity using an open-source platform to analyze and share worm behavior (Javer et al., 2018a, 2018b; Barlow et al., 2022). This work presents a novel strategy for mining orbitides in plants with diverse cyclic peptide origins and structures and offers a feasible platform for elucidating the cyclization mechanisms of cyclic peptides in plants.

## Methods

### Chemicals and reagents

All chemicals and reagents were purchased from commercial vendors. The authentic standard of HB (CAS:145 459-19-4) was obtained commercially from Yuanye (Shanghai, China). Other authentic standards, including PE, PF, PG, and four novel orbitides, cyclo-[GLPIGAPWG], cyclo-[LDGPPPYF], cyclo-[WGSSTPHT], and cyclo-[FGDVGPVI], were chemically synthesized by ZPC (Zhejiang, China) and verified by LC-MS.

### Collection of plant materials

*Pseudostellaria heterophylla* ("Kangbing No.1" cultivar, Linyi, Shandong province, China) seeds were soaked overnight in water. The moisture on the seed surface was maintained for one week, and the seeds were cultured in an incubator at 15°C for germination. The germinated seeds were sown in soil and grown in a phytotron at 25°C for 2 months. These seedlings are used for subsequent VIGS experiments. Flowers, leaves, stems, and roots of the "Zheseng No.2" cultivar of *P. heterophylla* (Zherong, Fujian province, China) were collected and quickly frozen in liquid nitrogen for orbitide content analysis and RNA-sequencing. Tuberous roots with buds from different cultivars were collected and planted in field plots at the Agricultural Genomics Institute at Shenzhen (Shenzhen, Guangdong, China) during the winter and collected the following summer. Tuber roots were used to mine precursor peptide genes and determine the orbitide content.

### Identification of PreCPs, PCYs, and OLPs in *P. heterophylla*

Previously reported precursor peptide genes (PreCPs) of *S. vaccaria* and *P. heterophylla* were used as query sequences, and the PreCP sequences were extracted by BLASTP comparison with the transcript library. Considering that the coding sequence (CDS) of PreCP was extremely short (∼108 base pairs [bp]), novel PreCP sequences were screened from the transcript libraries, which were assembled with transcriptome data from different cultivars using Trinity software (version 2.5.1) (Grabherr et al., 2011). PreCP sequences are listed in Supplemental Note 1. Given that PCY1 and OLP1 may be involved in the excision and cyclization of precursor peptide sequences (Craik and Malik, 2013), sequences of a serine protease, a cysteine protease, nine *OLP1* genes, and six *PCY1* genes that belong to the protease family in the *P. heterophylla* genome data are listed in Supplemental Note 2.

### RNA-sequencing and correlation analysis

Total RNA was extracted from tissues of different *P. heterophylla* cultivars, as described above, using the FastPure® Plant Total RNA Isolation Kit (Vazyme). The Micropoly (A) Purist mRNA Purification Kit was used to purify mRNA from total RNA. Sequencing libraries were created and sequenced on the Illumina HiSeq 2500 platform to generate paired-end 125-bp reads. De novo and genome-guided transcriptome assembly was performed using Trinity (Version 2.5.1) with default parameters (Grabherr et al., 2011). Expression abundance was estimated with Topmast and Cufflinks using HTSeq (Version 0.6.1) (Trapnell et al., 2012). Pearson correlation coefficients between cyclic peptide contents and candidate gene expression were calculated using Microsoft Excel, and Cytoscape software (Version 3.9.1) was used to visualize the data.

### Plasmid construction

The target fragment of the gene of interest was amplified using KOD One PCR Master Mix (Toyobo) and gel purified with a DNA purification kit (Gel and PCR Clean-up, NucleoSpin). The pEAQ-HT vector was linearized using *Age*I and *Xho*I restriction enzymes (NEB), and the pTRV vector was linearized using *Eco*RI and *Bam*HI (NEB). All target fragments were introduced into vectors using the ClonExpress II One Step Cloning Kit (Vazyme). The primers are listed in Supplemental Tables 1 and 2. Assembled plasmids were transformed into JM109 (pEAQ-HT vector) and DH5α (pTRV vector) chemically competent cells and cultured overnight on selective Luria–Bertani (LB) plates containing 50 μg mL^−1^ kanamycin. Subsequently, positive transformants were screened by PCR, used to inoculate 5 mL of liquid LB, and cultured at 37°C with shaking (200 rpm). Plasmid extraction was performed using a TIANprep Mini Plasmid Kit (TIANGEN®), and the insert sequences were confirmed using Sanger sequencing (Shangya Biotechnology Co., Ltd., China). The verified plasmids were stored at −20°C for subsequent chemical transformation into the *Agrobacterium tumefaciens* strain GV3101.

### *Agrobacterium*-mediated transient expression in *Nicotiana benthamiana*

Plasmids containing the target fragment of the gene of interest were transformed into *Agrobacterium tumefaciens* GV3101 (Rif^R^, AC1001L, WEIDI) using the freeze-thaw method and selected on LB agar plates containing 50 μg mL^−1^ kanamycin and 25 μg mL^−1^ rifampicin at 28°C. Positive transformants were confirmed by PCR, used to inoculate 5 mL of liquid LB, and cultured at 28°C for 2 days. Cell culture (100 μL) was plated on previously-described double antibiotic resistance plates and incubated at 28°C overnight. Subsequently, these bacteria were collected and resuspended in induction buffer (10 mM MES buffer, pH 5.6, 10 mM MgCl_2_, and 100 μM AS). Strain mixtures for co-expressing several candidate genes were maintained at an OD_600_ infiltration constant of 1.0. Cell suspensions were incubated at 28°C for 2 h before infiltration into *N. benthamiana* leaves.

*N. benthamiana* was seeded onto cylindrical rock wool blocks (20 mm × 27 mm, Grodan) and covered with vermiculite immersed in Hoagland’s nutrient solution (Coolaber). Thereafter, seedlings were thinned twice over the next 2–3 weeks to leave a single plant on each rockwool block. These cylindrical rock wool blocks were then transferred to square rock wool blocks (50 mm × 50 mm × 40 mm, Grodan) for further rapid growth under a 16/8-h light/dark cycle for another 2–3 weeks. Three leaves were chosen for infiltration into the previously mixed suspensions to minimize any batch effects among leaves in different positions on the plants. Qualitative experiments were repeated three times on different tobacco plants. Quantitative experiments were performed on more than three plants for each combination. Infiltrated *N. benthamiana plants* were grown without light for 24 h and then under light conditions for 5 days before samples were collected. All samples were flash frozen using liquid nitrogen.

### Metabolite extraction

Different tissues and heterogeneously expressed *N. benthamiana* leaves were ground into powder at 60 Hz for 45 s with a Tissuelyser tissue grinder (Tissuelyser-48L, Shanghai Jingxin) under liquid nitrogen quick-freezing conditions. We weighed 100 mg (when extracting tobacco leaf samples, we need accurately weigh 500 mg powder) of *P. heterophylla* powder into a 2 mL (15 mL) centrifuge tube and added 1 mL (5 mL) of chromatographic-grade methanol. Subsequently, these samples were thoroughly mixed using a vortex mixer (Vortex 3, IKA) and extracted at 40 kHz for 30 min at room temperature using an ultrasonic cleaner (SB25-12DT, SCIENTZ). These samples were then centrifuged at 4000 rpm for 15 min, and the supernatant was transferred to a new centrifuge tube. Tobacco samples were concentrated to 1 mL using a vacuum freezing centrifugal concentrator (CV600, JM) at 4°C and 1300 rpm, and all samples were treated with OASIS PRIME HLB (Waters) to remove pigments and impurities. These samples were filtered with an organic phase filter membrane (0.22 μm) using a blunt-end syringe and stored in brown sample vials for later analysis.

### Enzyme activity assay for PhPCY3 *in vitro*

Two potential substrates, HB [14-35] (linear IFGGLPPPSQELINGDDISLMV) and HB [14-21] (linear IFGGLPPP), as well as a standard for HB [22-35] (linear SQELINGDDISLMV), were synthesized by Sangon Biotech (Shanghai) Co., Ltd., and used to determine the catalytic activity of PhPCY3 *in vitro*. We constructed the prokaryotic expression vector for this gene using pMAL-c5x with an MBP tag, while the negative control contained the empty plasmid (pMAL-c5x). Briefly, the pMAL-c5x vector was linearized through double digestion with *Bam*HI and *Eco*RI restriction enzymes (NEB). The primer sequences are listed in Supplemental Table 1. As previously described in the plasmid construction section, the vector was also assembled via homologous recombination. The resultant plasmid was introduced into the *Escherichia coli* strain BL21, and a positive clone was selected for induced expression and purification experiments. Expression and purification followed the protocols described in the NEBExpress MBP Fusion and Purification System manual (NEB #E8200S). The purified proteins were quantified using the BCA method and analyzed using SDS-PAGE. The induced expression and purification of proteins (250 ng/μL) were performed for subsequent enzyme activity experiments using 30 μM substrates, 20 mM Tris-HCl (pH 8.5), 5 mM dithiothreitol, and 100 mM NaCl in a 200 μL reaction system at 30°C for 1 h. Subsequently, an equal volume of methanol was used to extract the reaction products. After centrifugation (4°C, 5000 *g*, 5 min), the supernatant was analyzed using a Q Exactive HFX (LC-MS).

### Quantitative and qualitative LC-MS analysis

The quantitative and qualitative analyses of the samples were conducted using two different LC-MS instrument setups: (1) Various *P. heterophylla* tissues and transiently expressing *N. benthamiana* leaves were evaluated using an UltiMate 3000 HPLC and Vanquish TSQ Quantum Access Max (Thermo) using electrospray ionization (ESI) in negative ionization mode with authentic standards. Quantitative analysis was performed by diluting the standard into different concentrations to precisely quantify the known orbitides. The detection method for HB, PE, PF, and PG was established in selective reaction monitoring (SRM) mode using an XBridge BEH C18 Column (Waters, 130 Å, 2.5 μm, 2.1 mm × 100 mm). The mobile phases consisted of acetonitrile (A) and 0.1% formic acid water (B), with a flow rate of 0.3 mL/min. The column temperature was maintained at 40°C, and the sample injection volume was fixed at 5 μL. Specific mobile phase elution gradients and mass spectrometry fragment ion information are provided in Supplemental Method 1. (2) Heterogeneously expressed novel orbitides in *N. benthamiana* leaves and the *in vitro* enzymatic products were detected using an UltiMate 3000 HPLC and Q Exactive HFX Mass Spectrometer (Thermo Scientific) in the full scan/ddMS2 mode. These tobacco samples underwent qualitative analysis using an ACQUITY UPLC BEH C18 VanGuard Pre-column (Waters, 130 Å, 1.7 μm, 2.1 mm × 5 mm) to determine the precise molecular weights of newly identified CPs. The mobile phase and flow velocity were consistent with those previously described. The LC-MS methods are detailed in Supplemental Method 1. Raw data were analyzed using Thermo Xcalibur software (Version 4.1) with either the Qual Browser or the Quan Browser app.

### *In vivo* activity verification via the VIGS platform

The VIGS Tool (https://vigs.solgenomics.net/) was used to design silenced target sequences. Target gene segments were assembled into the pRTV2 vector by homologous recombination, as described above. The positive plasmids were transformed into the *Agrobacterium strain* GV3101, and the screening, activation, culturing, and resuspension of strains followed the same procedures detailed in *Agrobacterium*-mediated transient expression. Cell suspensions were mixed with a strain containing the pTRV1 vector and inoculated into the leaves of 3-week-old plants. Each treatment was applied to three pots of plants. *PhChlH1* was used as a positive control and exhibited a leaf-yellowing phenotype. Four weeks after *Agrobacterium* infiltration, the newly grown leaves at the top of the plants showed a stable yellow leaf phenotype, and different tissues were then collected for subsequent metabolic detection and gene expression analysis.

### Quantitative real-time PCR analyses

VIGS samples were ground into powder using a Tissuelyser tissue grinder and total RNA was extracted using a FastPure Plant Total RNA Isolation Kit (Vazyme). To synthesize the first-strand cDNA, 1 μg of mRNA was mixed with TransScript All-in-One First-Strand cDNA Synthesis SuperMix (AT341-01, TRAN) to generate the cDNA template. The PCR reaction system (15 μL) included 0.6 μL of cDNA template, 7.5 μL of 2×TransStart Green qPCR SuperMix, and 0.45 μL of 10 mM upstream and downstream primers. The qRT-PCR primers are listed in Supplemental Table 3. Quantitative real-time PCR was performed using the Bio-Rad CFX Opus 96 Real-Time System, and the relative expression level of each gene was calculated using the 2 ^(−ΔΔCt)^ method compared with an internal control gene (β-actin) (Livak and Schmittgen, 2001). Three biological replicates were analyzed to determine the average value.

### Molecular docking and sequence alignment

The protein structures of PhPCYs were predicted using AlphaFold (https://www.alphafold.ebi.ac.uk/). The chemical structure of the target substance was drawn using ChemDraw 20.0 software and converted to a MOL2 format file using Chem3D software. AutoDock Tools (Version 1.5.7) was used to prepare protein models and substrates, namely PreHB [14-35], PrePE [14-36], PrePF [14-35], and PrePG [14-35]. The molecular docking of PreCPs [14-35/36] with PhPCY3 was performed using AutoDock Vina (Forli et al., 2016). Subsequently, the interactions between the substrate ligand and receptor protein in 4 Å were visualized using PyMOL software. The key catalytic sites were identified using multiple sequence alignment (Multailin 5.4.1) and protein structure analysis, combined with information on molecular docking residues. The residues regarded as key sites of PhCPY3 involved in orbitide cyclization were mutated to their counterparts in PhPCY1/2. The plasmid pEAQ-HT-PhPCY3 (WT) was used as the template, and the primers for site-directed mutagenesis are listed in Supplemental Table 4. Site-directed mutagenesis was carried out using PCR and assembled with a multi-fragment homologous recombination kit (ClonExpress Ultra One Step Cloning Kit, C115, Vazyme). The resultant strains harboring the *PhPCY3* mutants were co-expressed with *PhPreHB* and assayed in the *N. benthamiana* system, as described above.

## Data availability

The data that support the findings of this study are available in the supplemental information of this article. We have uploaded raw sequencing reads (RNA-sequencing) to the NCBI SRA database under accession number SUB14839290.

## Funding

This work was supported by the 10.13039/501100012166National Key R&D Program of China (Grant No. 2020YFA0907900 and 2017YFE0121800), and the 10.13039/501100001809National Natural Science Foundation of China (Grant No. 32170264, Grant No. 81573530, and Grant No. 31401950).

## Acknowledgments

We thank Dr. Ran Du for his support with chemical analysis, and Yanchun Peng and Meifeng Su from the Metabolic Platform at the AGIS for their assistance with metabolism analysis. We also thank Zhexue Sun, Hailang Xu, Fancheng Zeng, Congrong Jiang, Wenwen Zhang, Kui Wang, and Mingze Ma for their help in collecting and grinding samples. There are no conflicts of interest among the participants.

## Author contributions

X.Q. and W.Li designed the research; X.Q. performed the research; F.W. and D.X. provided the analytical tools; X.Q. and Q.Z. collected VIGS samples; S.L. and W.Lin provided genome and transcriptome data; X.Q. and W.Li wrote and revised the paper.

## Supplemental information

Document S1. Supplemental Figures 1–17, Supplemental Tables 1–4, and Supplemental Method 1

Supplemental Note 1. The CDS sequences of precursor peptide genes in *P. heterophylla* The fasta file describes the CDS sequences of precursor peptide genes in different cultivated strains of *P. heterophylla*

Supplemental Note 2. Sequences of the candidate genes that may be involved in orbitide biosynthesis The word file described the CDS sequences of PhSP, PhCP, PhOLPs, and PhPCYs.

Document S2. Article plus supplemental information
