# Supplementary material for: Identification of a key peptide cyclase for novel cyclic peptide discovery in Pseudostellaria heterophylla
Source: Plant Commun. 2025 Mar 13;6(5):101315. doi: 10.1016/j.xplc.2025.101315 (PMC12143145; doi:10.1016/j.xplc.2025.101315)
Supplement: Document S1. Supplemental Figures 1–17, Supplemental Tables 1–4, and Supplemental Method 1 [file mmc1.pdf]

Plant Communications, Volume *6*

Supplemental information

Identification of a key peptide cyclase for novel cyclic peptide discovery in *Pseudostellaria heterophylla*

Xianjin Qin, Fengjiao Wang, Dejin Xie, Qi Zhou, Sheng Lin, Wenxiong Lin, and Wei Li

Supplemental information

Identification of a key peptide cyclase for novel cyclic peptide discovery in *Pseudostellaria heterophylla*

Xianjin Qin, Fengjiao Wang, Dejin Xie, Qi Zhou, Sheng Lin, Wenxiong Lin and Wei Li

***Correspondence: Wenxiong Lin** (lwx@fafu.edu.cn), **Wei Li** (liwei11@caas.cn).

Supplemental information

Article title: **Identification of a key peptide cyclase for novel cyclic peptide discovery in *Pseudostellaria heterophylla***

Authors: Xianjin Qin, Fengjiao Wang, Dejin Xie, Qi Zhou, Sheng Lin, Wenxiong Lin and Wei Li

This PDF file includes:

Supplemental Figures 1 to 17
Supplemental Tables 1 to 4
Supplemental Method 1
Legends for Supplemental Note 1 to 2

Other supporting materials for this manuscript include the following:

Supplemental Note 1 to 2

Supplemental Figure 1. Chemical structure and chromatograms of CPs in *P. heterophylla.*

Supplemental Figure 2. Particle fragment information of HB and PE/PF/PG in secondary mass spectrometry.

Supplemental Figure 3. Potential biosynthesis pathway of orbitides in *P. heterophylla*.

Supplemental Figure 4. Phylogenetic analysis of the PhPCYs genes with other known cyclase gene *SvPCY1* and *HaAEP*.

Supplemental Figure 5. Two specific daughter ion fragment information of peaks in different groups in the selected Ion monitoring (SRM) mode.

Supplemental Figure 6. *PhOLP1* and *PhOLP2* were not involved in the cyclization of Heterophyllin B.

Supplemental Figure 7. *PhPCY3* cyclizes different *PhPreCPs* into orbitides in the transient expression platform.

Supplemental Figure 8. The SDS-PAGE image shows the purification of pMAL-MBP-tag and PhPCY3 proteins for the *in vitro* assay.

Supplemental Figure 9. MS2 particle fragment information of HB and HB [22-35] in the enzyme activity assay of Ph PCY3 with HB [14-35] *in vitro*.

Supplemental Figure 10. VIGS the *PhPreCP* genes involved in orbitides biosynthesis *in planta*.

Supplemental Figure 11. Amino acid sequence alignment and mutation site analysis of *PhPCY1/2/3* and *SvPCY1*.

Supplemental Figure 12. Key catalytic sites identified via molecular docking.

Supplemental Figure 13. Molecular docking of the substrate PE [14-36] with PhPCY3 within 4 Å and triad amino acids.

Supplemental Figure 14. Molecular docking of the substrate PF [14-35] with PhPCY3 within 4 Å and triad amino acids.

Supplemental Figure 15. Molecular docking of the substrate PG [14-35] with PhPCY3 within 4 Å and triad amino acids.

Supplemental Figure 16. The ion chromatograms (SRM) of heterologous

expression mutations of PhPCY3 with PhPreHB in *N. benthamiana* leaves.

Supplemental Figure 17. Reverse genetics strategy for mining novel orbitides via a transient expression platform.

Supplemental Table 1. Primers for all the candidate genes with pEAQ-HT and pMal-c5x vectors.

Supplemental Table 2. Primers for the candidate genes with pTRV2 vector.

Supplemental Table 3. qRT-PCR primers for precursor peptide genes and PhPCY3.

Supplemental Table 4. Primers for site-directed mutation at N500 and S502 site of PhPCY3.

Supplemental Method 1. Supplementary methods for LC-MS analysis.

Supplemental Note 1. The CDS sequence of precursor peptide genes in *P. heterophylla*.

Supplemental Note 2. Sequences of the candidate genes that may be involved in orbitide biosynthesis.

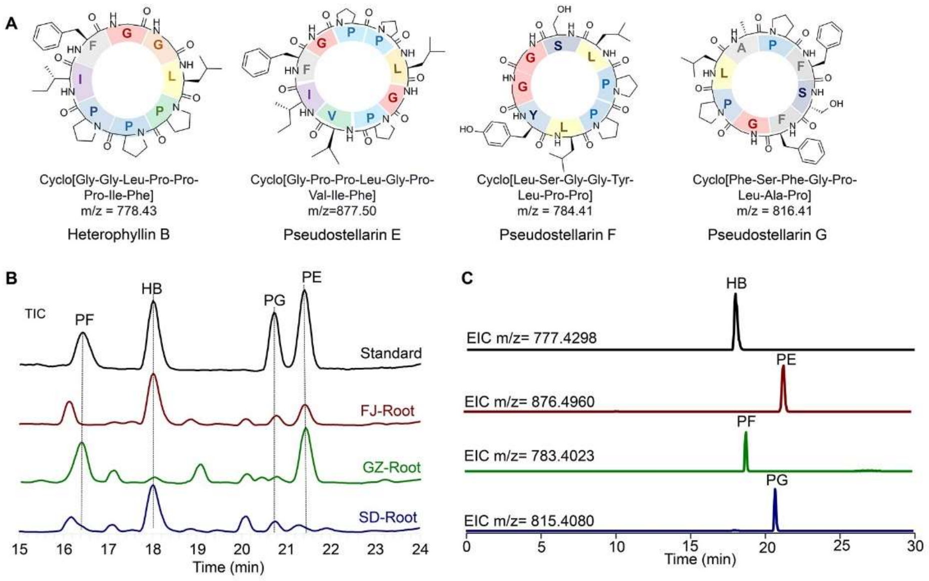

Supplemental Figure 1. Chemical structure and chromatograms of orbitides in *P. heterophylla.*

(A) Chemical structure of heterophyllin B and pseudostellarin E/F/G.

(B) The total ion chromatograms (TICs) of the root extracts of the cultivars (FJ-ZS II: Main cultivar cultivated in Fujian province, GZ-ST: Main cultivar cultivated in Guizhou province, SD-KB: Main cultivar cultivated in Guizhou province) with the known orbitides (including heterophyllin B and pseudostellarin E/F/G) using Q Exactive^{TM} HFX LC-MS/MS.

(C) Extracted ion chromatograms (EICs) of HB, PE, PF, and PG.

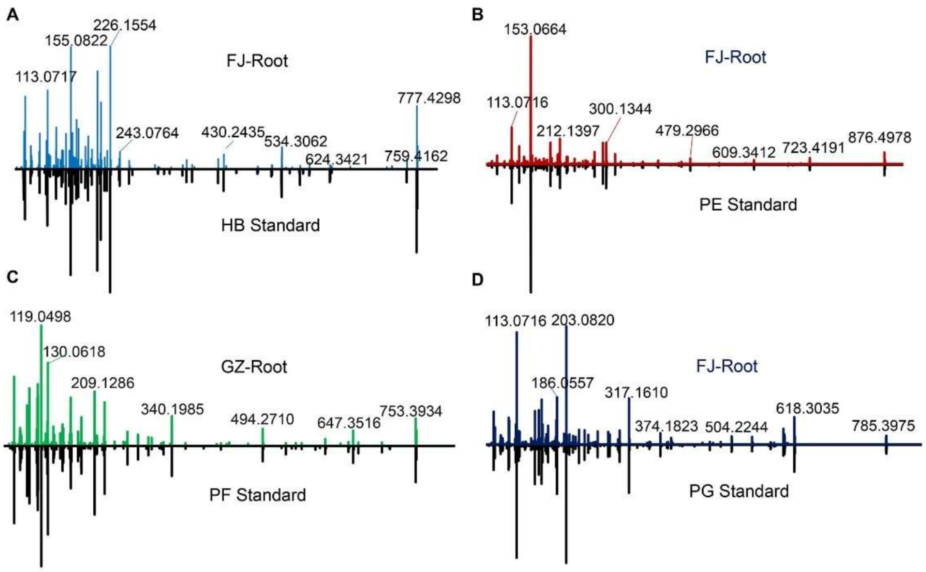

Supplemental Figure 2. Particle fragment information of HB and PE/PF/PG in secondary mass spectrometry.

(A) Particle fragment information of HB in secondary mass spectrometry (MS2).

(B) Particle fragment information of PE in secondary mass spectrometry (MS2).

(C) Particle fragment information of PF in secondary mass spectrometry (MS2).

(D) Particle fragment information of PG in secondary mass spectrometry (MS2).

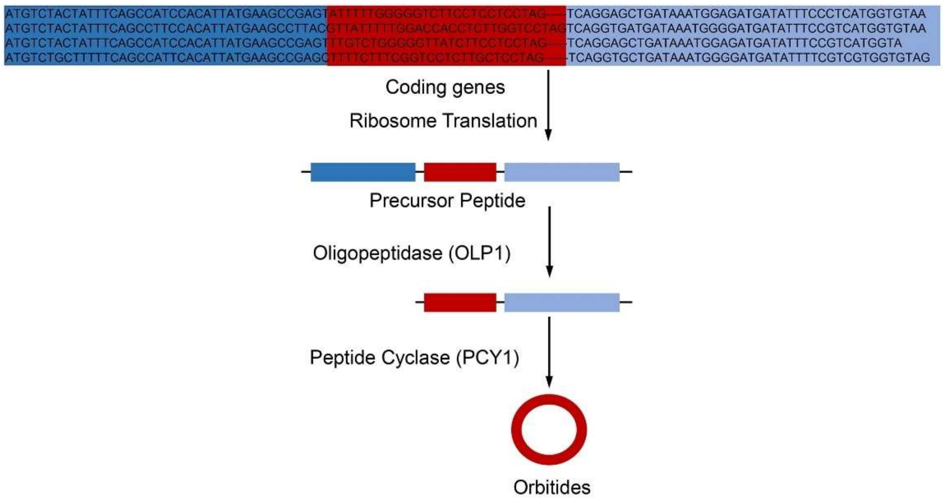

Supplemental Figure 3. Potential biosynthesis pathway of orbitides in *P. heterophylla*.

The precursor peptide genes were translated into linear precursor peptides. Oligopeptidase (OLP1) and peptide cyclase (PCY1) may be involved in excise at the N-terminal and the cyclize at the C-terminal.

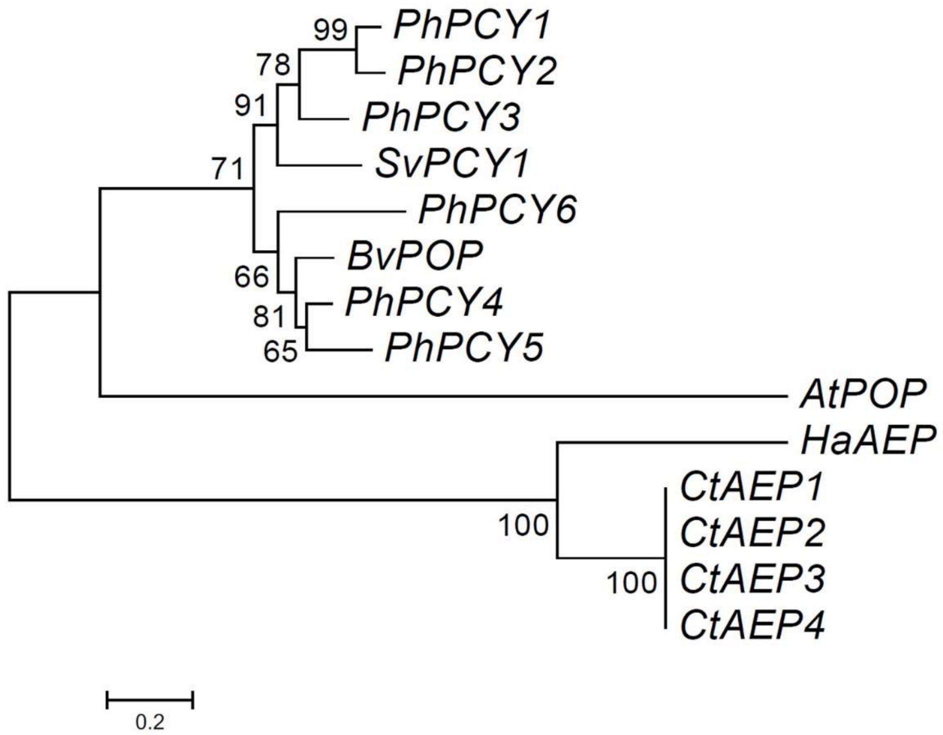

Supplemental Figure 4. Phylogenetic analysis of the PhPCYs genes with other known cyclase gene *SvPCY1* and *HaAEP*.

The maximum likelihood tree was constructed by Mega software. Bootstrap values are indicated in the nodes (based on 1000 replicates). *SvPCY1*: *PCY1* gene from *Saponaria vaccaria*, *BvPOP* and *AtPOP* refers to the prolyl oligopeptidase gene from *Beta vulgaris* and *Arabidopsis thaliana,* respectively. *HaAEP* and *CtAEP1/2/3/4* refer to asparaginyl endopeptidase gene from *Helianthus annuus* and *Clitoria ternatea*. PhPCYs refer to *PhPCY1*: *PhPCY1-Ctg883.47*, *PhPCY2*: *PhPCY1-Ctg883.48*, *PhPCY3*: *PhPCY1-Ctg883.50, PhPCY4*: *PhPCY1-Ctg2037.21*, *PhPCY5*: *PhPCY1-Ctg3671.23*, *PhPCY6*: *PhPCY1-Ctg3671.6*, respectively.

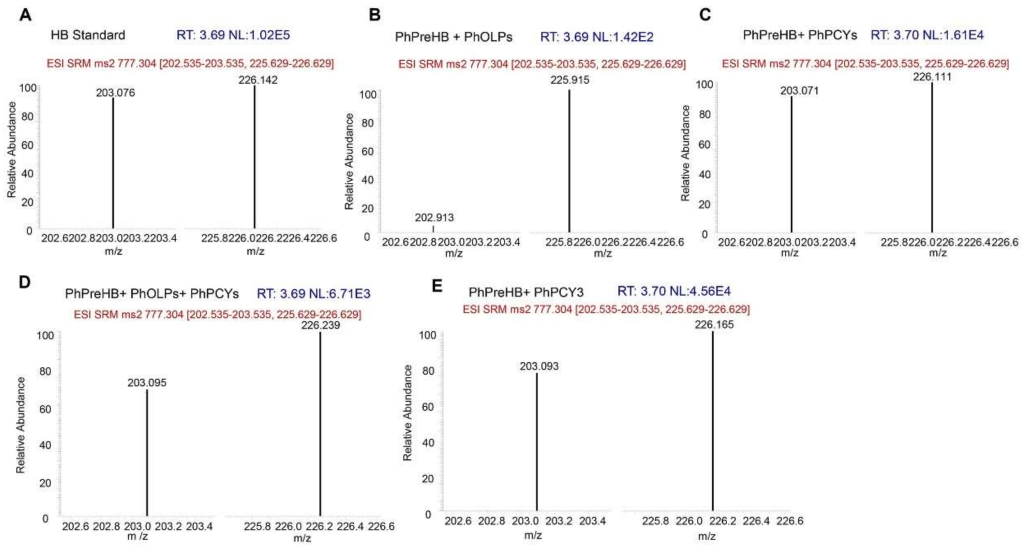

Supplemental Figure 5. Two specific daughter ion fragment information of peaks in different groups in the selected Ion monitoring (SRM) mode.

(A) Two specific daughter ion fragment information of the HB peak in SRM mode (the peak was HB standard).

(B) Two specific daughter ion fragment information of the PhPreHB + PhOLPs group peak in SRM mode (the tiny peak was not HB).

(C) Two specific daughter ion fragment information of the PhPreHB + PhPCYs group peak in SRM mode.

(D) Two specific daughter ion fragment information of the PhPreHB +PhOLPs + PhPCYs group peak in SRM mode.

(E) Two specific daughter ion fragment information of the PhPreHB +PhPCY3 group peak in SRM mode.

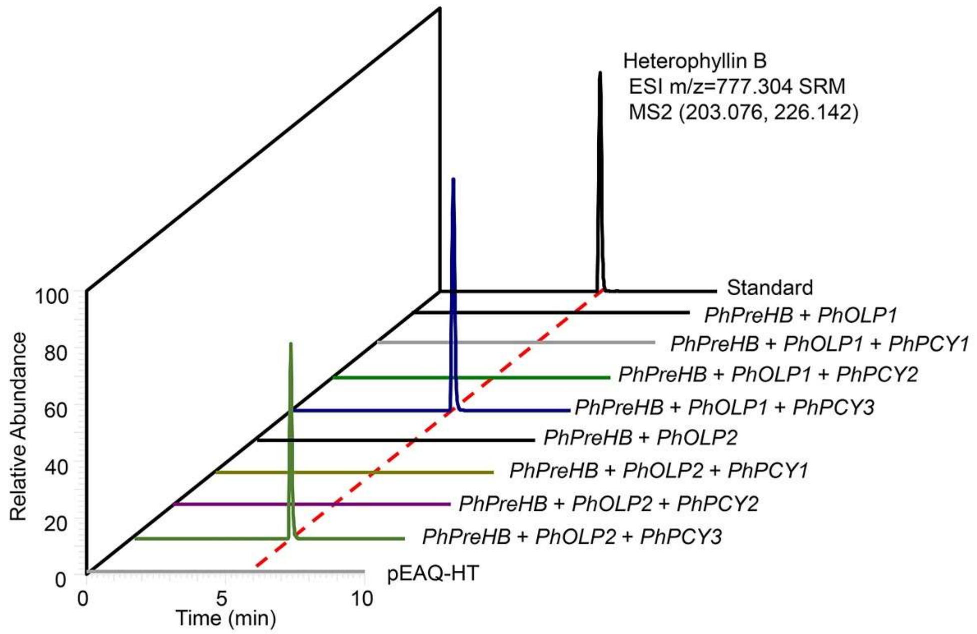

Supplemental Figure 6. *PhOLP1* and *PhOLP2* were not involved in the cyclization of Heterophyllin B.

The ion chromatograms of *N. benthamiana* leaf samples which combine *PhPreHB* with *PhOLP1/2* and *PhPCY1/2/3* were detected using LC-MS in the selected reaction monitoring (SRM) mode. *PhOLP1* refers to *PhOLP1-Ctg4232.8* and *PhOLP2* refers to *PhOLP1-Ctg5689.43.*

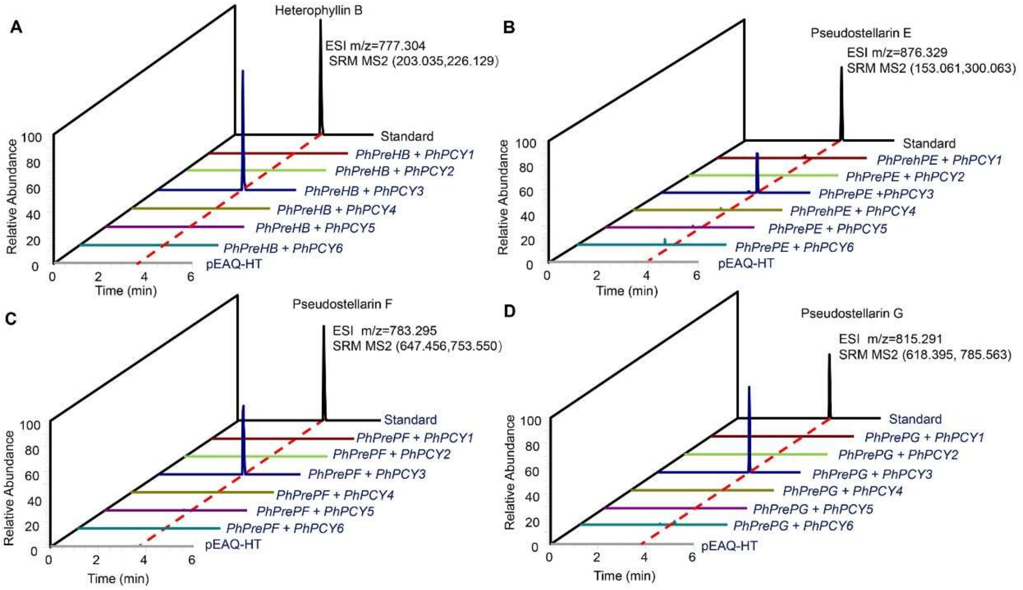

Supplemental Figure 7. PhPCY3 cyclizes *PhPreCPs* into orbitides in transient expression platform.

(A) *PhPCY3* can cyclize *PhPreHB* to heterophyllin B.

(B) *PhPCY3* cyclizes *PhPrePE* to pseudostellarin E.

(C) *PhPCY3* can cyclize *PhPrePF* to pseudostellarin F.

(D) *PhPCY3* can cyclize *PhPrePG* to pseudostellarin G.

The ion chromatograms of *N.benthamiana* leaf samples which combine *PhPreHB*, *PhPrePE*, *PhPrePF,* and *PhPrePG* with *PhPCY1/2/3/4/5/6* were detected using LC-MS in the selected reaction monitoring (SRM) mode, respectively.

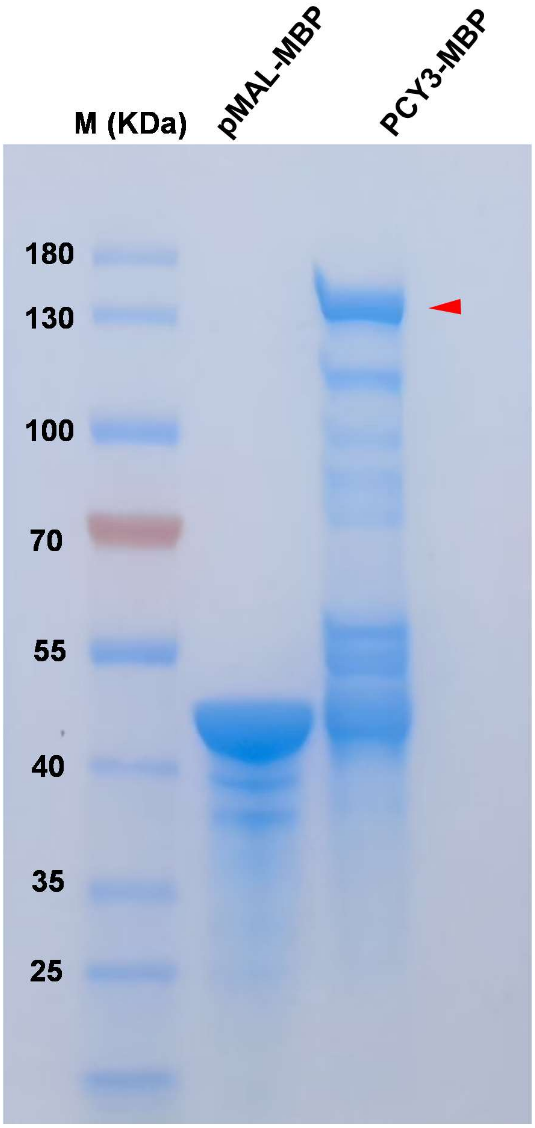

Supplemental Figure 8. The SDS-PAGE image shows the purification of pMAL-MBP-tag and PhPCY3 proteins for the *in vitro* assay. The MBP tag ia about 42.5 KDa, and PhPCY3 is about 80.7 KDa. Thus, the size of the fusion expression protein with MBP tag after induction is about 123.3 KDa (The red arrows indicate fusion expressed proteins).

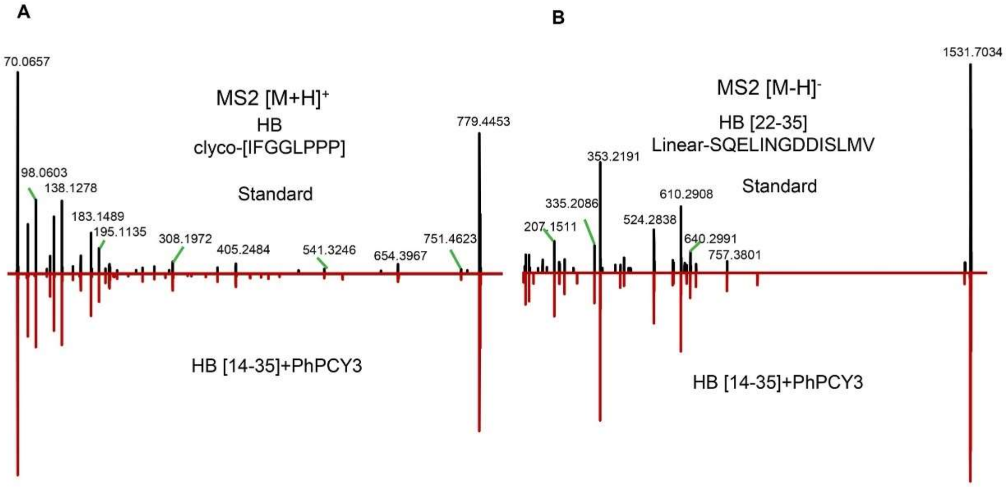

Supplemental Figure 9. MS2 particle fragment information of HB and HB [22-35] in the enzyme activity assay of PhPCY3 with HB [14-35] *in vitro*.

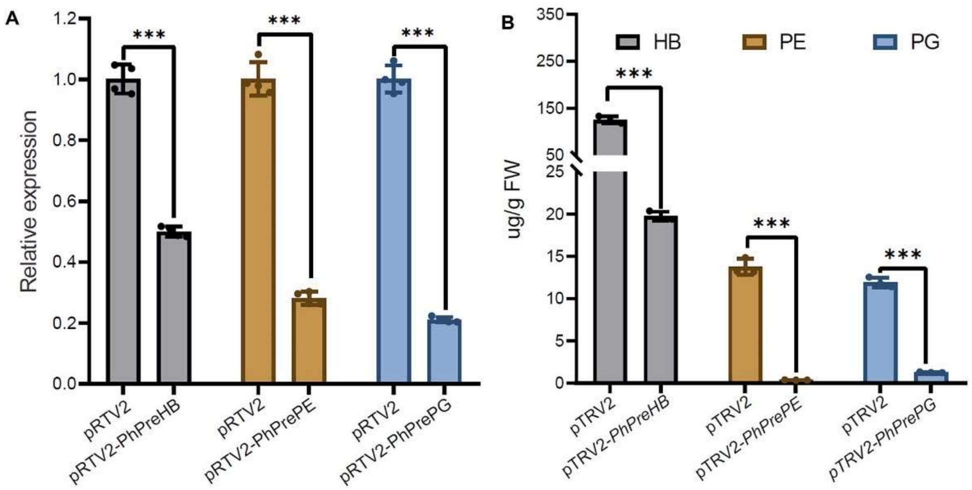

Supplemental Figure 10. VIGS the *PhPreCP* genes involved in orbitides biosynthesis *in planta*.

(A) The relative expression of the *PhPreHB*, *PhPrePE,* and *PhPrePG* gene *in P. heterophylla* leaves when these genes were silenced *in planta*.

(B) The HB, PE, and PG contents *in P. heterophylla* roots when these genes were silenced *in planta*.

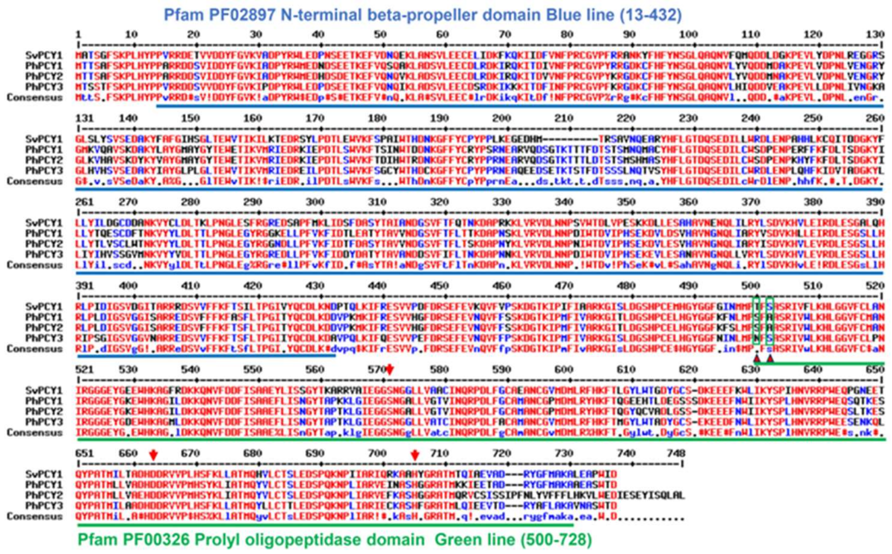

Supplemental Figure 11. Amino acid sequence alignment and mutation site analysis of *PhPCY1/2/3* and *SvPCY1*.

The PCYs have beta-propeller domain (13-432) at the N-terminal (Pfam PF02897) under the blue line and prolyl oligopeptidase domain (500-728) at the C-terminal (Pfam PF00326) under the green line.The conserved triad amino acids are marked with red arrows (S562, D653, H695 in SvPCY1 and S571, D662, H704 in PhPCY3). Meanwhile, N500 and S502 sites were highlighted with green text box and red triagnle.

Substrate Name	Substrate PreCPs [14-35/36]																						
	14	15	16	17	18	19	20	21	22	23	24	25	26	27	28	29	30	31	32	33	34	35	36
PreHB [14-35]	I	F	G	G	L	P	P	P	S	Q	E	L	I	N	G	D	D	I	S	L	M	V	
Residue Sites (AA)	V84	G401	G402							M498	R505	G570	N594	R707		S502	N500	K414	N104		Q712		
																A503							
																S504							
PrePE [14-36]	V	I	F	G	P	P	L	G	P	S	Q	V	M	I	N	G	D	D	I	S	V	M	V
Residue Sites (AA)							K250			E356	N494			N594	M498		H488	N594		R719	S502		N500
										R369	N496						G570	R719			S504		
																					R505		
PrePF [14-35]	L	S	G	G	Y	L	P	P	S	Q	E	L	I	N	G	D	D	I	S	V	M	V	
Amino Acid (AA)		R88			H133	H133					N104		K414			N500	H488	N594	S502		N500	N500	
		S136									T709					R505	G570	R719	S504				
		V137									Q712												
PrePG [14-35]	F	S	F	G	P	L	A	P	S	Q	V	L	I	N	G	D	D	I	S	V	M	V	
Residue Sites (AA)	N594	R719		N500	K414		K414		G401	V84			Q103	R81		G706			N500			N496	
	R719													R707		R707							
														T709		Q712							
														Q712									

Supplemental Figure 12. Key catalytic sites identified via molecular docking.

Hydrogen-bonding interactions in 4 Å were analyzed between the residue sites of PhPCY3 and the substrate PreCPs [14-35/36], and the residues binding position at the substrate were also identified and recorded. The core peptide regions have the pink background color and the follower peptide at C termial have the blue background color, while the common residue site N500 and S502 has a red background.

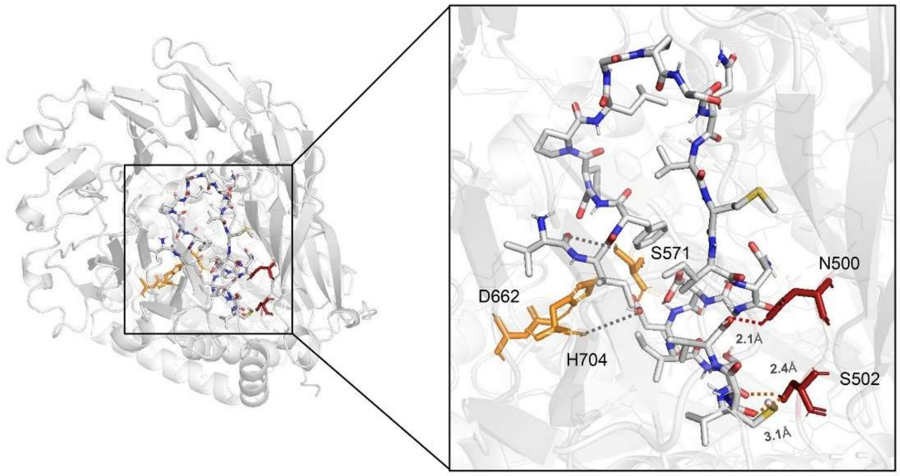

Substrate PE [14-36] molecular docking with PhPCY3

Supplemental Figure 13. Molecular docking the substrate PE [14-36] with PhPCY3 within 4Å and triad amino acids.

Hydrogen-bonding interactions between the residue sites (N500 and S502) of PhPCY3 and the substrate PE [14-36] (color the sticks with heteroatom) within 4 Å were highlighted by dark red dashed lines. The triad amino acids (S571, D662 and H704) are highlighted with orange sticks in the molecular docking. The triad residues nearest to oxygen atom in the substrate are highlighted by the gray dashed lines.

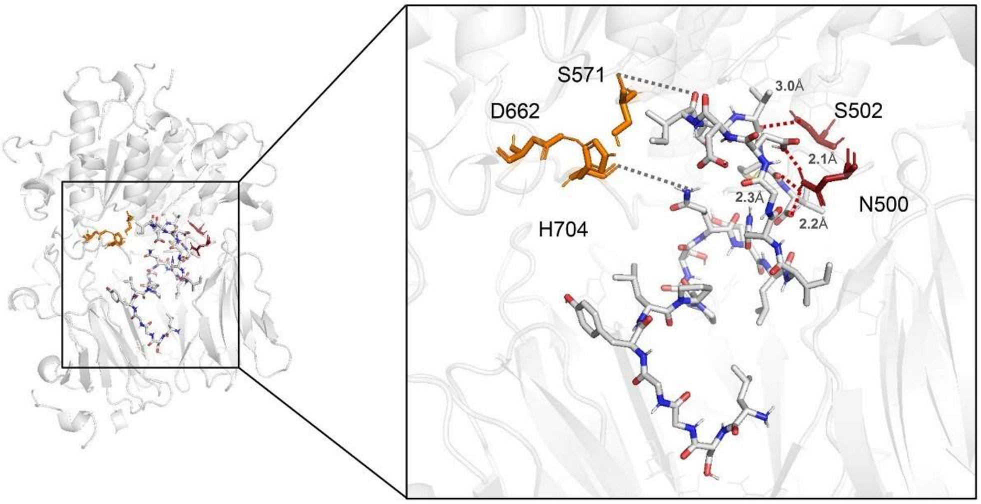

Substrate PF [14-35] molecular docking with PhPCY3

Supplemental Figure 14. Molecular docking the substrate PF [14-35] with PhPCY3 within 4Å and triad amino acids.

Hydrogen-bonding interactions between the residue sites (N500 and S502) of PhPCY3 and the substrate PF [14-35] (color the sticks with heteroatom) within 4 Å were highlighted by dark red dashed lines. The triad amino acids (S571, D662 and H704) are highlighted with orange sticks in the molecular docking. The triad residues nearest to oxygen atom in the substrate are highlighted by the gray dashed lines.

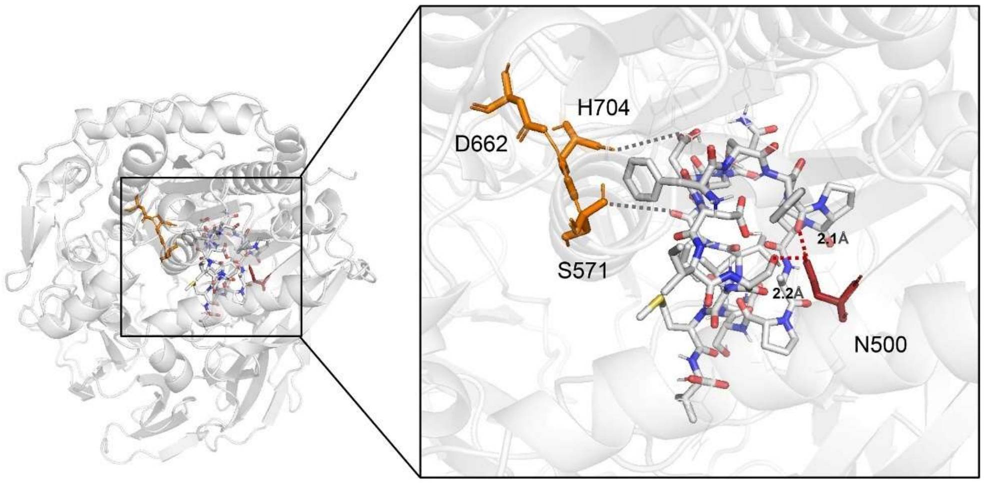

Substrate PG [14-35] molecular docking with PhPCY3

Supplemental Figure 15. Molecular docking the substrate PG [14-35] with PhPCY3 within 4Å and triad amino acids.

Hydrogen-bonding interactions between the residue site (N500) of PhPCY3 and the substrate PG [14-35] (color the sticks with heteroatom) within 4 Å were highlighted by dark red dashed lines. The triad amino acids (S571, D662 and H704) are highlighted with orange sticks in the molecular docking. The triad residues nearest to oxygen atom in the substrate are highlighted by the gray dashed lines.

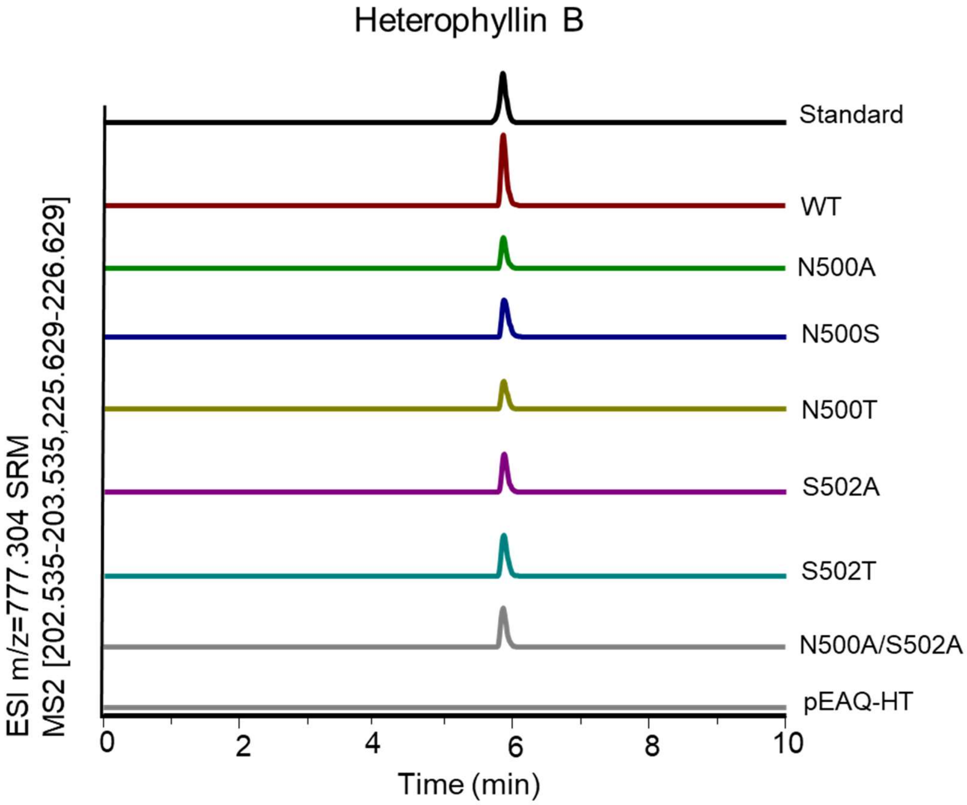

Supplemental Figure 16. The ion chromatograms (SRM) of heterologous expression mutations of *PhPCY3* with *PhPreHB* in *N. benthamiana* leaves. The ion chromatograms (SRM) of *N. benthamiana* leaf samples which have expressed *PhPreHB* with *PhPCY3* (WT) (or *PhPCY3* mutants at the N500 and S502 sites, including N500A, N500S, N500T, S502A, S502T, N500A/S502A) were detected using LC-MS in the selected reaction monitoring (SRM) mode, respectively.

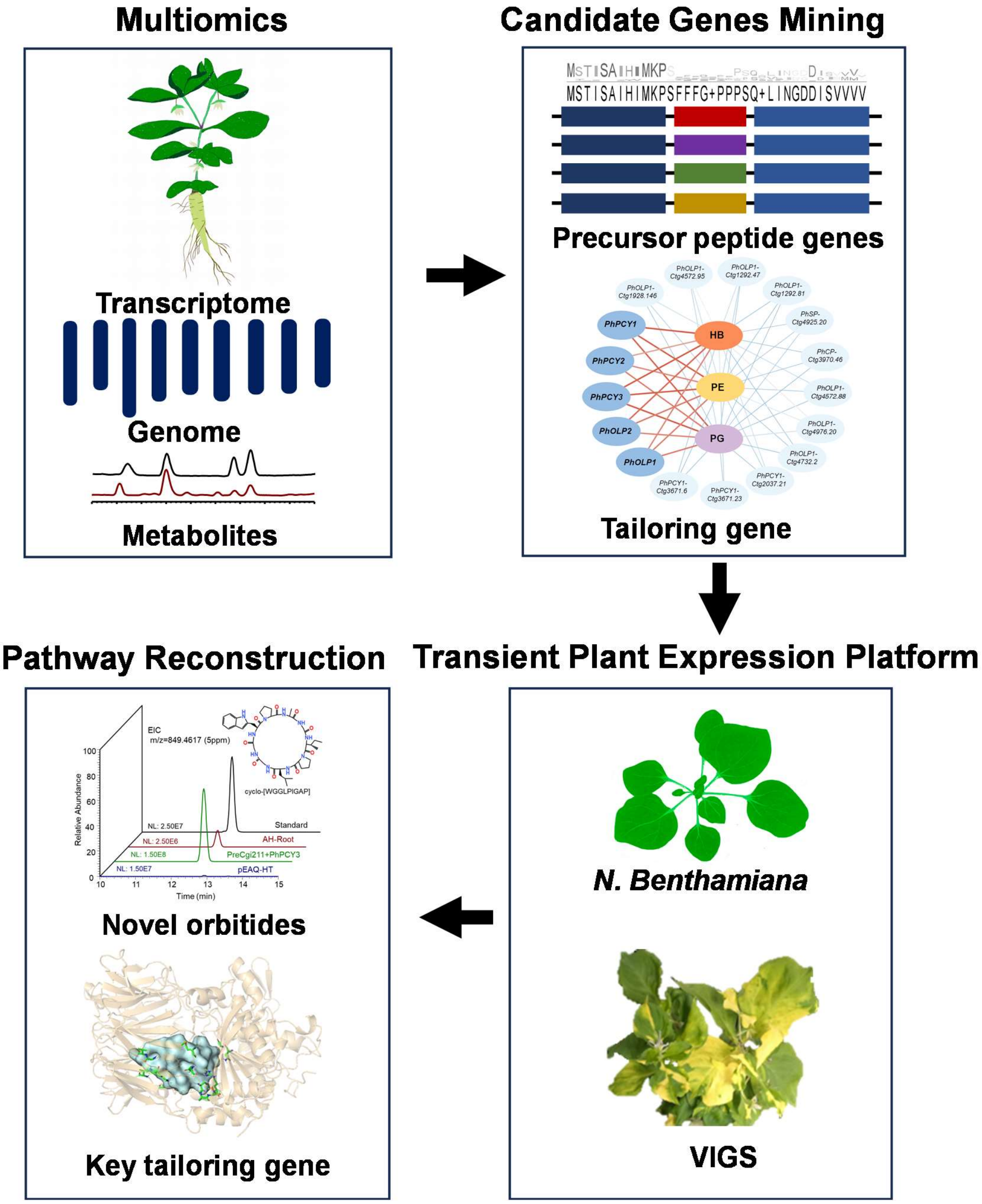

Supplemental Figure 17. Reverse genetics strategy for mining novel orbitides via a transient expression platform. Multiple omics data have been used to explore the candidate genes in the orbitide biosynthesis pathway. Their functions *in vivo* were elucidated using the heterologous expression system in *N. benthamiana* and the virus-induced gene silencing (VIGS) system in *P. heterophylla*. Novel orbitides were discovered through pathway reconstruction with the key tailoring gene.

Supplemental Table 1. Primers for all the candidate genes with pEAQ-HT and pMAL-c5x vectors. The primers were designed by CE Design V 1.04 software, the pEAQ-HT vector was linearized at *Age*I and *Xho*I sites and the pMAL-c5x vector was linearized at *Bam*HI and *Eco*RI sites.

Full Names	Primer Names	Primer Sequences (5'-3')
PhPreHB	pEAQ-PreHB-F	caaattcgcgaccggtATGTCTACTATTTCAGCCATCCACATTATGAAG
	pEAQ-PreHB-R	agttaaaggcctcgagTTACACCATGAGGGAAATATCATCTCCATTTATCAG
PhPrePE	pEAQ-PrePE-F	caaattcgcgaccggtATGTCTACTATTTCAGCCTTCCACATTATGAAG
	pEAQ-PrePE-R	agttaaaggcctcgagTTACACCATGACGGAAATATCATCCCCAT
PhPrePF	pEAQ-PrePF-F	caaattcgcgaccggtATGTCTACTATTTCAGCCATCCACATTATGAAG
	pEAQ-PrePF-R	agttaaaggcctcgagTTATACCATGACGGAAATATCATCTCCATTTATCAGC
PhPrePG	pEAQ-PrePG-F	caaattcgcgaccggtATGTCTACTATTTCAGCCATCCACATTATGAAG
	pEAQ-PrePG-R	agttaaaggcctcgagTTAGACCATGACGGAAATATCATCCCCATT
PhOLP1-	pEAQ-OLP1-F	caaattcgcgaccggtATGGAGAAATCTAGCAAGAATATGAACAAAATCATGG
Ctg4232.8	pEAQ-OLP1-R	agttaaaggcctcgagCTAACATATTGGTAAATCTTCTGGTGGAGACT
PhOLP1-	pEAQ-OLP2-F	caaattcgcgaccggtATGGCATTCAAAAAAACACTAGTACAAAGATTATTCAAC
Ctg5689.43	pEAQ-OLP2-R	agttaaaggcctcgagCTAATTAACCTTATGGTAATGATCATACTTGATCAACCTAG
PhPCY1-	pEAQ-PCY1-F	caaattcgcgaccggtATGACTACCTCCGCCTTCTCCA
Ctg883.47	pEAQ-PCY1-R	agttaaaggcctcgagTTAATCCGTCCAAGAGGCTTCAGCT
PhPCY1-	pEAQ-PCY2-F	caaattcgcgaccggtATGACTACCTCCGCCTTCTCCA
Ctg883.48	pEAQ-PCY2-R	agttaaaggcctcgagTCATAATGCCAGTTGAGAAATATACTCACTCTCTATGT
PhPCY1-	pEAQ-PCY3-F	caaattcgcgaccggtATGACTTCCTCCACCTTCTCCAAAC
Ctg883.50	pEAQ-PCY3-R	agttaaaggcctcgagTTAATCTGTCCAAGAGGCGTTGACCG
PhPCY1-	pEAQ-PCY4-F	caaattcgcgaccggtATGAGCACGCCTCTATTACGATTACG
Ctg2037.21	pEAQ-PCY4-R	agttaaaggcctcgagTTAGTCAATCCAAGAAGCATTCACCACT
PhPCY1-	pEAQ-PCY5-F	caaattcgcgaccggtATGATGTCTTCATCTGTCTCCGCC
Ctg3671.23	pEAQ-PCY5-R	agttaaaggcctcgagTTAATCAGTCCACGGGGAATTCACGAC
PhPCY1-	pEAQ-PCY6-F	caaattcgcgaccggtATGGGCGTTGAAGTGTCGTTG
Ctg3671.6	pEAQ-PCY6-R	agttaaaggcctcgagTTAGTTGATCCATGACACATCCATCATCTTAGC
pMAL-c5x-	pMAL-PCY3-F	CGAGATATCGTCGACGGATCCATGACTTCCTCCACCTTCTCCA
PhPCY3-	pMAL-PCY3-R	TTAATTACCTGCAGGGAATTCTTAATCTGTCCAAGAGGCGTTGA

Supplemental Table 2. Primers for the candidate genes with pTRV2 vector. VIGS Tool in Solanaceae Genomics Network (https://vigs.solgenomics.net) has been used to analyze the target sequences. The primers were designed by CE Design V 1.04 software and the vector was linearized at *Eco*RI and *Bam*HI sites.

Primer Names	Primer Sequences (5'-3')
pTRV2-*PhPreHB-F*	gtgagtaaggttaccgaattcATGTCTACTATTTCAGCCATCCACA
pTRV2-*PhPreHB-R*	cgtgagctcggtaccggatccTTACACCATGAGGGAAATATCATCTC
pTRV2-*PhPrePE-F*	gtgagtaaggttaccgaattcATGTCTACTATTTCAGCCTTCCACAT
pTRV2-*PhPrePE-R*	cgtgagctcggtaccggatccTTACACCATGACGGAAATATCATCC
pTRV2-*PhPrePG-F*	gtgagtaaggttaccgaattcATGTCTACTATTTCAGCCATCCACA
pTRV2-*PhPrePG-R*	cgtgagctcggtaccggatccTTAGACCATGACGGAAATATCATCC
pTRV2-*PhChlH 1-F*	gtgagtaaggttaccgaattcTGAACAGGCCAATACCCTGG
pTRV2-*PhChlH 1-R*	cgtgagctcggtaccggatccAAGTAGTGGCTCACATCAGTTAAAGAA
pTRV2-*PhPCY3-F*	gtgagtaaggttaccgaattcAAAGTACTCGCCGCTCCATAAC
pTRV2-*PhPCY3-R*	cgtgagctcggtaccggatccGCGTTGACCGCCTTTGCC

Supplemental Table 3. qRT-PCR primers for precursor peptide genes and *PhPCY3*. The primers were designed by Primer Premier 5.0 software.

Primer Names	Primer Sequences (5'-3')
Phβ-Actin-F	CTGTATTTACGCTCAGGTGG
Phβ-Actin-R	CATTGTGCTCAGTGGTGG
PhPreHB-F	CATCCACATTATGAAGCCGAGTA
PhPreHB-R	CCCAACGGTGACGGACAT
PhPrePE-F	ATGTCTACTATTTCAGCCTTCCAC
PhPrePE-R	TCATCCCCATTTATCATCACC
PhPrePG-F	CAGCCATTCACATTATGAAGCC
PhPrePG-R	CATCCCCATTTATCAGCACC
PhPCY3-F	TGGCACAAGGCAGGAATG
PhPCY3-R	CGTTATGGAGCGGCGAGTA

Supplemental Table 4. Primers for site-directed mutation at N500 and S502 site of PhPCY3. The primers were designed by CE Design V 1.04 software and the pEAQ-HT vector was linearized at *Age*I and *Xho*I sites.

Primer Names	Primer Sequences (5'-3')
PhPCY3(WT)-F1	ctgcccaaattcgcgaccggtATGACTTCCTCCACCTTCTCCA
PhPCY3(WT)-R2	accagagttaaaggcctcgagTTAATCTGTCCAAGAGGCGTTGA
PhPCY3-N500A-F2	GTTTAACATAAACTTGATGCCAGCTTTTTCTGCTAGTCGTATAGTG
PhPCY3-N500A-R1	CACTATACGACTAGCAGAAAAAGCTGGCATCAAGTTTATGTTAAAC
PhPCY3-N500S-F2	GTTTAACATAAACTTGATGCCATCTTTTTCTGCTAGTCGTATAGTG
PhPCY3-N500S-R1	CACTATACGACTAGCAGAAAAAGATGGCATCAAGTTTATGTTAAAC
PhPCY3-N500T-F2	GTTTAACATAAACTTGATGCCAACTTTTTCTGCTAGTCGTATAGTG
PhPCY3-N500T-R1	CACTATACGACTAGCAGAAAAAGTTGGCATCAAGTTTATGTTAAAC
PhPCY3-S502A-F2	ATAAACTTGATGCCAAATTTTGCTGCTAGTCGTATAGTGTGGCT
PhPCY3-S502A-R1	AGCCACACTATACGACTAGCAGCAAAATTTGGCATCAAGTTTAT
PhPCY3-S502T-F2	ATAAACTTGATGCCAAATTTTACTGCTAGTCGTATAGTGTGGCT
PhPCY3-S502T-R1	AGCCACACTATACGACTAGCAGTAAAATTTGGCATCAAGTTTAT
PhPCY3-N500A&S502A-F2	GTTTAACATAAACTTGATGCCAGCTTTTGCTGCTAGTCGTATAGTGTGGCTG
PhPCY3-N500A&S502A-R1	CAGCCACACTATACGACTAGCAGCAAAAGCTGGCATCAAGTTTATGTTAAAC

Supplemental Method 1. Supplementary methods for LC-MS analysis.

(a) HPLC/TSQ-MS (Thermo Scientific™)

Detection Method: LC-MS/MS---SRM

Instrument: UltiMate 3000 HPLC + TSQ Quantum Access Max

Mode: Negative

Column: XBridge BEH C18 Column (Waters, 130 Å, 2.5 μm, 2.1 mm × 100 mm)

Mobile phase: Solvent A: Acetonitrile;

Solvent B: Water with 0.1% (v/v) formic acid

Flow rate: 0.3 mL/min

Injection volume: 5.0 μl

Mobile phase elution gradient (See the table below for details)

Time	Solvent A (%)	Solvent B (%)
0	10	90
1.0	10	90
4.5	90	10
4.5	90	10
4.6	10	90
6.0	10	90
6.0	Stop Run	

Particle fragment information [M-H]-：

Parent M	Product M	CE	T lens
777.304	203.035	48	132
777.304	226.129	49	132
783.295	647.456	36	99
783.295	753.550	26	99
815.291	618.395	33	104
815.291	785.563	26	104
876.329	153.061	58	145
876.329	300.063	54	145

Mass spectrometer parameters:

Instrument	TSQ Quantum Access
Mode	ESI positive
Vaporizer Temperature	320°C
Capillary Temperature	340°C
Spray Voltage	3600 V
Sheath Gas Pressure	35 L/h
Aux Gas Pressure	10 L/h
Spare Gas Pressure	0.0 L/h
Scan Width	1.0 m/z
Scan Time	0.08

(b) HPLC/ Q Exactive HFX MS (Thermo Scientific™)

Detection Method: Full scan/ddMS2 mode

Instrument: UltiMate 3000 HPLC + Q Exactive™ HFX Mass Spectrometer

Mode: Negative and Positive

Column: ACQUITY UPLC BEH C18 VanGuard Pre-column

(Waters, 130 Å, 1.7 μm, 2.1 mm × 5 mm)

Mobile phase: Solvent A: Acetonitrile

Solvent B: Water with 0.1% (v/v) formic acid

Flow rate: 0.3 mL/min

Injection volume: 5.0 μL

Mobile phase elution gradient (See the table below for details)

Time	Solvent A (%)	Solvent B (%)
0	10	90
2.0	10	90
15.0	95	5
20.0	95	5
20.0	95	5
25.0	10	90
25.0	Stop Run	

Mass spectrometer parameters:

Instrument	Q Exactive™ HFX MS
Ion Source	HESI
Vaporizer Temperature	320°C
Capillary Temperature (+ or + -)	340°C
Capillary Temperature (-)	350°C
Spray Voltage (+)	3000 V
Spray Voltage (-)	3500 V
Sheath Gas Pressure (+ or + -)	35 L/h
Sheath Gas Pressure (-)	35 L/h
Aux Gas Pressure (+ or + -)	10 L/h
Aux Gas Pressure (-)	10 L/h
Spare Gas Pressure (+ or + -)	0.0 L/h
Spare Gas Pressure (-)	0.0 L/h
Probe Heater Temp (+ or + -)	340°C
Probe Heater Temp (-)	375°C
S-Lens RF Level	50.00

Supplemental Note 1. The CDS sequence of precursor peptide genes in *P. heterophylla*. The fasta file described the CDS sequences of the precursor peptide genes in different cultivated *P. heterophylla*.

Supplemental Note 2. Sequences of the candidate genes that may be involved in orbitide biosynthesis. The word file described the CDS sequences of PhSP, PhCP, PhOLPs and PhPCYs.

>PhPCY1-Ctg883.50 **(PhPCY3)**

ATGACTTCCTCCACCTTCTCCAAACCCTTGCACTACCCGCCTGTCCGCCGCGACGAT
TCCGTCGTCGACGACTATTTCGGTGTCAAAATCCCCGACCCATACCGTTGGCTGGAG
GATCCAGATTCGGAAGAGACGAAAGAGTTTGTACAGAATCAAATAAAGCTTGCGGATT
CAGTGCTCGAAGAGTGCGATTCAAGAGACAAAATCAAGAAGAAAATCACTGATTTTAT
CAATTTTCCGCGTTGTGGTGTTCCATTTAAGCGCGGTGATAAGTGTTTTCATTTTTATA
ATTCTGGCCTTCAGGCGCAAAATGTGCTTCATATTCAGGATGATGTGGAAGCAAAGC
CAGAGGTGCTACTTGATCCTAACCTTATTGTGAACGGAAAAGCTGGTTTGCACGTGC
ATTCTGTAAGCGAGGACGCCAAATATATTGCATACGGTCTGCCTTTAGGTTTGACTGA
ATGGGTGACTATCAAAGTAATGAGAATTGAAGACCGAGAAATTTTACCAGACACTTTAT
CGTGGGTGAAGTTTAGTGGTTGTTATTGGACCCATGACTGTAAAGGATTTTTCTATTG
CCCGTATCCACCCCGCAATGAAGCACAAGAAGAAGATTCTGAAACTAAGACTTCTAC

CTTCGATACTAGTTCTAGCTTGAATCAGACGGTAAGCTATCATTTTCTCGGCACGGAT
CAGTCTGAAGATATTCTGTGCTGGAGAGACCTTGAGAACCCTTTACAACACTTTAAGA
TAGATGTAACTGCTGACGGAAAGTATCTTCTTATCTATATTCATGTGAGCTCTGGTGTG
ATGAACAAAGTATACTATGTGGATTTAACAACGCTGCCTAATGGGCTTGAAGGTTATC
GTGGACGAGAAGACTTGCTTCCTTTCGTAAAGTTTATTGATGATTATGATGCAACGTAT
ACAGCCGTTGCTAATGATGACTCTGTGTTTATTTTCCTAACTAACAAGGACGCTCCCA
ATAATAAGTTGGTTCGTGTTGATTTGAATAATCCCGACATATGGACTGATGTGATTCCA
CATTCTGAGAAGGAAGTGCTTGAATCAGCAAATGCTGTTAATGGAAATCAGCTTCTTG
TCCGTTACCTAAGCGATGTCAAGCATGTTCTTGAAGTTAGAGATCTTGAGAGTGGCTC
CTTGCTGCATCGCATACCGTCAGGCATCGGATCTGTTGGTGGAGTTAATGCTCGACG
GGAAGATAGTGTCGTGTTTTTTAAATTCACAAGCTTCCTGACTCCTGGCATTATTTACC
AATGTGATTTGAAAGATGCTGTTCCACAGCTGAAGATTTTTCAAGAAAGTGTTGTCCC
TGAATTTGACCGTTCTGAGTTTGAGGTTAATCAGGTTTTTTTTCCCAGCAAAGATGGT
ACAAAGATACCAATGTTCATAGTGGCGAGAAAGGGAATTTCTTTGGACGGATCACAC
CCATGTGAACTGCATGGTTATGGCGGGTTTAACATAAACTTGATGCCAAATTTTTCTG
CTAGTCGTATAGTGTGGCTGAAACACCTTGGTGGAGTCTTCTGCTTGCCTAATATCCG
AGGTGGTGGTGAATATGGAGATGAATGGCACAAGGCAGGAATGCTTGATAAGAAGCA
GAATGTTTTTGATGACTTCATCTCTGCAGCTGAGTTTCTTATTTCTAATGGCTATACCG
CACCTACAAAATTGGGTATTGAAGGTGGAAGTAACGGTGGCCTTCTTGTTGCTACCT
GTATTAATCAGAGACCAGACCTTTTTGCTTGCGCTATGGCAAACTGTGGTGTAATGGA
CATGCTTCGATTCCATAAATTTACCATGGGTTATCTTTGGACGGCGGATTATGGTTGCT
CTGAAAAAGAGGAAGACTTCAACTGGCTTATAAAGTACTCGCCGCTCCATAACGTGA
GGCGGCCATGGGAGGAATCAGAGAATAAACAGTTACAGTACCCTGCTACAATGATATT
AGCAGCTGATCATGATGATCGTGTCGTGCCTCTGCACTCCTTTAAACTGTTGGCTACT
ATGCAGTACCTTCTTTGCACAACTTTGGAGGACAGCCCGCAGAAGAATCCGTTAATT
GCTCGAATTGAGTGCAAAGCTTCACACTTTGGACGTGCGACTATGTTGCAGATTGAG
GAAGTTACAGATCGGTATGCCTTTCTGGCAAAGGCGGTCAACGCCTCTTGGACAGAT
TAA
