# Supplementary material for: Identification of a key peptide cyclase for novel cyclic peptide discovery in Pseudostellaria heterophylla
Source: Plant Commun. 2025 Mar 13;6(5):101315. doi: 10.1016/j.xplc.2025.101315 (PMC12143145; doi:10.1016/j.xplc.2025.101315)
Supplement: Supplemental Note 2. Sequences of the candidate genes that may be involved in orbitide biosynthesis — The word file described the CDS sequences of PhSP, PhCP, PhOLPs, and PhPCYs. [file mmc3.pdf]

>PhPCY1-Ctg883.47 **(PhPCY1)**
ATGACTACCTCCGCCTTCTCCAAACCCTTGCACTACCCTCCCGCCCGCCGCGACGACTCTGTCA
TTGATGACTACTTCGGTGTCAAAATCGCTGACCCGTATCGTTGGATGGAGGATAATGATTCGGA
AGAGACGAAGGAGTTTGTACAGAGTCAAGCGAAGCTTGCAGATTCGGTGCTGGAAGAATGCGA
TTTGAGAGACAAAATCAGGCAGAAAATCACTGATATTGTCAATTTTCCGCGGTGTGGTGTGCCA
TATAGGCGTGGTGATAAGTGTTTTCACTTTTATAATTCTGGCCTTCAGGCGCAAAATGTGCTTTAT
GTTCAGGACGATATGGACGCAAAGCCAGAAGTGCTAGTTGATCCTAACCTTGTTGAGAATGGAA
GATATGGTATGAAAGTGCAAGCTGTTAGCAAGGATGCCAAATATCTTGCATATGGTATGGCTTAT
GGCTACACTGAATGGGAGACGATCAAAGTAATGAGAATTGAAGACCGGAAGATTGAACCCGAC
ACTTTATCTTGGGTGAAGTTTACTAGTATTAATTGGACTGATGACAATAAAGGATTCTTTTATTGC
CGGTATCCATCCCGCAATGAAGCACGAGTACAAGATTCCGGAACTAAGACTACTACCTTCGATA
CTAGTACTAGCATGAATCAAATGGCATGTTATCATTTTCTTGGGACCGACCAGTCTGAAGATATT
CTGTGCTGGAGTGACCCTGAGAACCCAGAACGGTTCTTTAAGTTCGATTTGACTTCTGATGGCA
AGTATATTCTCCTCTATACTCAAGAGAGCTGTGATTTTACGAACAAAGTATACTATTTGGATTTAA
CAACGCTGCCTAACGGGCTTGAAGGTTACCGCGGGGGAAAAGAGTTGCTTCCTTTCGTAAAGTT
TATTGATACTCTTGAAGCGACGTATACAGCTGTTGTTAATGATGGCTCTGTGTTTACTTTCCTAAC
TACTAAGGACGCTCCAAATAGTAAGTTAGTTCGTGTTGATTTGAATAACCCGGACATATGGACTG
ATGTGATTCCACATTCTGAGAAGGACGTGCTTGATTCAGTGCATGCTGTTAATGGAAACCAGCTT
ATTGCACGTTATGTTAGCGATGTCAAACATCTTCTCGAGATTAGAGATCTTGAGAGTGGCTCCCT
GCTGCATCGTTTACCCTTAGACATCGGATCTGTTGGTGGAATTTCTGCACGACGGGAAGATAGT
GTCTTCTTTTTCAAGTTTGCAAGCTTCCTGACTCCTGGCATTACTTATCAATGTGATTTGAAAGAT
GATGTTCCAAAGATGAAGATTTTCCGAGAAAGTGTTGTCCATGGATTTGATCGTTCCGAGTTTGA
GGTTAACCAGGTTTTTTTTTCCAGCAAAGATGGCACAAAGATACCAATGTTCATAGTGGCAAGA
AAGGGAATTACCTTGGACGGATCACACCCATGTGAACTTCATGGTTATGGCGGGTTTAAATTTA
ACTTGATGCCAAGTTTTGCCGCCAGTCGCATAGTGTGGCTGAAGCACCTTGGTGGGGTCTTCTG
CATGGCTAATATCAGAGGCGGTGGTGAATATGGAAAAGAATGGCACAAGGCAGGAATTCTTGA
TAAGAAGCAGAATGTTTTTGACGACTTCATCTCTGCAGCTGAGTTTCTTATTTCTAATGGCTATAC
CGCGCCTAAAAAATTGGGGATTGAAGGTGGAAGTAATGGTGCGCTTCTTGTTGGGACTGTTATT
AATCAGAGACCAGACCTTTTTGGTTGCGCTATGGCAAACTGCGGTCCAATGGATATGCTTCGAT
ACCATAAGTTTACCCAAGGTGAGGAACACACTCTAGATGAAGGAAGTTCCTCCGATAAAGAGG
AAGAGTTCAACTGGATTATAAAGTACTCACCACTCCATAACGTGAGGAGGCCTTGGGAGCAATC
ACAGACTAAAGAGTCACAATACCCTGCTACTATGTTATTGGTTGCTGATCACGATGATCGTGTCG
TGCCTATGCACTCCTATAAACTGATCGCTACTATGCAGTACGTTCTCTGCACTAGTTTGGAGGAC
AGCCCTCAGAAGAATCCATTAATAGCTCGAGTTGAGATCAACGCTTCACATGGTGGACGTGCGA
CTATGAAGAAGATTGAAGAAACTGCAGACCGGTATGGGTTTATGGCGAAGGCAGCTGAAGCCT
CTTGGACGGATTAA
>PhPCY1-Ctg883.48 **(PhPCY2)**
ATGACTACCTCCGCCTTCTCCAAACCCTTGCACTACCCTCCCGCCCGCCGCGACGACTCTGTCA
TTGATGACTACTTCGGTGTCAAAATCGCTGATCCGTATCGTTGGATGGAGGATCATGATTCTGAC
GAGACGAAGGAGTTTGTACAGAATCAAGTGAAGCTTGCAGATTCGGTGCTGGAAGAATGCGAT
TTAAGAGACAAAATAAGGCAGAAAATCACTGATGTTGTCAATTTTCCGCGCTGTGGTGTGCCAT
ATAAGCGCGGTGATAAGTGTTTTCACTTTTATAATTCTGGCCTTCAGGCGCAAAATGTGCTTTAT
GTTCAGGATGATATGAACGCAAAGCCAGAAGTGCTAGTTGATCCTAACCTTGTTGAGAACGGAA
GATATGGTTTGAAAGTGCACGCAGTAAGCAAGGATTACAAATATGTTGCGTATGGTATGGCTTA

TGGCTACACTGAATGGGAGACGATCAAAGTAATGAGAATTGAAGACCGGAAGATTGAACCCGA
CACTTTATCTTGGGTGAAGTTTACTGATATTCATTGGACTCGTGACAATAAAGGATTCTTTTATTG
CCGGTATCCACCCCGCAATGAAGCACGAGTACAAGATTCCGGTACTAAGACTACCACCTTAGAT
ACTAGTACTAGCATGAGTCATATGGCAAGCTATCATTTTCTTGGGACTGACCAGTCTGAAGATAT
TCTGTGCTGGAGTGACCCTGAGAACCCAAAACATTACTTTAAGTTCGATTTAACTTCTGATGGAA
AGTATATTCTTCTCTATACTCTAGTGAGCTGTCTTTGGACGAACAAAGTATACTATTTGGATTTAA
CAACGCTGCCTAATGGGCTTGAAGGTTACCGCGGGGGAAATGACTTGCTTCCTTTCGTAAAGTT
TATTGATACTTTTGACGCATCATATACAGCTGTTGTTAATGATGGCTCTGTGTTTACTTTCCTAACT
AGTAAGGATGCTCCGAATTATAAGTTGGTTCGTGTTGATTTGAATAATCCCAACATATGGACTGA
TGTGATTCCACATTCTGAGAAGGACGTGCTTGAATCAGTGCATGCTGTTAATGGAAACCAGCTT
ATTGCACGTTATATAAGTGATGTCAAACATGTTCTCGAGGTTAGAGATCTAGAGAGTGGTTCCCT
GCTGCATCGTTTACCCTTAGACATCGGATCTGTTGGTGGAATTTCTGCACGACGGGAAGATAGT
GTCTTCTTTTTCAAGTTTACAAGCTTCCTGACTCCTGGCATTACTTATCAATGTGATTTGAAAGAT
GATGTTCCAAAGATGAAGATTTTCCGAGAAAGTGTCGTCCATGGATTTGATCGTTCCGAGTTTGA
GGTTAACCAGGTTTTTTTTTCCAGCAAAGATGGTACAAAGATACCAATGTTCATAGTGGCAAGAA
AGGGAATTACCTTGGACGGATCACACCCATGTGAACTACATGGTTATGGTGGGTTTAAATTTAG
CTTGATGCCAAGTTTTGCCGCCAGTCGCATAGTGTGGCTGAAGCACCTTGGTGGGGTCTTCTGC
ATGGCTAATATCAGAGGCGGTGGTGAATATGGAAAAGAATGGCACAAGGCAGGAATTCTTGAT
AAGAAGCAGAATGTTTTTGACGACTTCATCTCTGCTGCTGAGTTTCTTATTTCTAACGGCTATACC
GCGCCTACAAAATTGGGGATTGAAGGTGGAAGTAATGGTGCGCTTCTTGTTGGGACTGTTATTA
ATCAGAGACCAGACCTTTTTGGTTGCGCTATGGCAAACTGCGGTCCAATGGATATGCTTCGATA
CCATAAGTTTACCCAAGGTTATCAATGTGTTGCGGATTTAGGTTCCTCCGATAAAGAGGAAGAG
TTCAACTGGATTATAAAGTACTCACCACTCCATAACGTGAGGAGGCCTTGGGAGCAATCACTGA
CTAAAGAGTCACAATACCCTGCTACAATGTTATTGGTAGCTGAGCACGATGATCGTGTCGTGCC
TATGCACTCCTATAAACTGATCGCTACTATGCAGTACGTTCTCTGCACAAGTTTGGAAGACAGCC
CTCAGAAGAATCCATTAATAGCTCGAGTTGAGTTCAAAGCTTCACACGGTGGACGTGCGACTAT
GCAGAGGGTCTGTAGTATTTCCTCCATCCCATTCAATCTTTACGTTTTTTTTTTTTTGCACAAGGTT
TTATGGGAGGACATAGAGAGTGAGTATATTTCTCAACTGGCATTATGA

>PhPCY1-Ctg883.50 **(PhPCY3)**

ATGACTTCCTCCACCTTCTCCAAACCCTTGCACTACCCGCCTGTCCGCCGCGACGATTCCGTCGT
CGACGACTATTTCGGTGTCAAAATCCCCGACCCATACCGTTGGCTGGAGGATCCAGATTCGGAA
GAGACGAAAGAGTTTGTACAGAATCAAATAAAGCTTGCGGATTCAGTGCTCGAAGAGTGCGATT
CAAGAGACAAAATCAAGAAGAAAATCACTGATTTTATCAATTTTCCGCGTTGTGGTGTTCCATTT
AAGCGCGGTGATAAGTGTTTTCATTTTTATAATTCTGGCCTTCAGGCGCAAAATGTGCTTCATATT
CAGGATGATGTGGAAGCAAAGCCAGAGGTGCTACTTGATCCTAACCTTATTGTGAACGGAAAA
GCTGGTTTGCACGTGCATTCTGTAAGCGAGGACGCCAAATATATTGCATACGGTCTGCCTTTAG
GTTTGACTGAATGGGTGACTATCAAAGTAATGAGAATTGAAGACCGAGAAATTTTACCAGACAC
TTTATCGTGGGTGAAGTTTAGTGGTTGTTATTGGACCCATGACTGTAAAGGATTTTTCTATTGCCC
GTATCCACCCCGCAATGAAGCACAAGAAGAAGATTCTGAAACTAAGACTTCTACCTTCGATACT
AGTTCTAGCTTGAATCAGACGGTAAGCTATCATTTTCTCGGCACGGATCAGTCTGAAGATATTCT
GTGCTGGAGAGACCTTGAGAACCCTTTACAACACTTTAAGATAGATGTAACTGCTGACGGAAAG
TATCTTCTTATCTATATTCATGTGAGCTCTGGTGTGATGAACAAAGTATACTATGTGGATTTAACA
ACGCTGCCTAATGGGCTTGAAGGTTATCGTGGACGAGAAGACTTGCTTCCTTTCGTAAAGTTTAT
TGATGATTATGATGCAACGTATACAGCCGTTGCTAATGATGACTCTGTGTTTATTTTCCTAACTAA

CAAGGACGCTCCCAATAATAAGTTGGTTCGTGTTGATTTGAATAATCCCGACATATGGACTGATG
TGATTCCACATTCTGAGAAGGAAGTGCTTGAATCAGCAAATGCTGTTAATGGAAATCAGCTTCTT
GTCCGTTACCTAAGCGATGTCAAGCATGTTCTTGAAGTTAGAGATCTTGAGAGTGGCTCCTTGCT
GCATCGCATACCGTCAGGCATCGGATCTGTTGGTGGAGTTAATGCTCGACGGGAAGATAGTGTC
GTGTTTTTTAAATTCACAAGCTTCCTGACTCCTGGCATTATTTACCAATGTGATTTGAAAGATGCT
GTTCCACAGCTGAAGATTTTTCAAGAAAGTGTTGTCCCTGAATTTGACCGTTCTGAGTTTGAGGT
TAATCAGGTTTTTTTTCCCAGCAAAGATGGTACAAAGATACCAATGTTCATAGTGGCGAGAAAG
GGAATTTCTTTGGACGGATCACACCCATGTGAACTGCATGGTTATGGCGGGTTTAACATAAACTT
GATGCCAAATTTTTCTGCTAGTCGTATAGTGTGGCTGAAACACCTTGGTGGAGTCTTCTGCTTGC
CTAATATCCGAGGTGGTGGTGAATATGGAGATGAATGGCACAAGGCAGGAATGCTTGATAAGA
AGCAGAATGTTTTTGATGACTTCATCTCTGCAGCTGAGTTTCTTATTTCTAATGGCTATACCGCAC
CTACAAAATTGGGTATTGAAGGTGGAAGTAACGGTGGCCTTCTTGTTGCTACCTGTATTAATCAG
AGACCAGACCTTTTTGCTTGCGCTATGGCAAACTGTGGTGTAATGGACATGCTTCGATTCCATAA
ATTTACCATGGGTTATCTTTGGACGGCGGATTATGGTTGCTCTGAAAAAGAGGAAGACTTCAACT
GGCTTATAAAGTACTCGCCGCTCCATAACGTGAGGCGGCCATGGGAGGAATCAGAGAATAAAC
AGTTACAGTACCCTGCTACAATGATATTAGCAGCTGATCATGATGATCGTGTCGTGCCTCTGCAC
TCCTTTAAACTGTTGGCTACTATGCAGTACCTTCTTTGCACAACTTTGGAGGACAGCCCGCAGAA
GAATCCGTTAATTGCTCGAATTGAGTGCAAAGCTTCACACTTTGGACGTGCGACTATGTTGCAGA
TTGAGGAAGTTACAGATCGGTATGCCTTTCTGGCAAAGGCGGTCAACGCCTCTTGGACAGATTA
A
>PhPCY1-Ctg2037.21 **(PhPCY4)**
ATGAGCACGCCTCTATTACGATTACGATTACGATTACTTCCCTCCTACGCCTATGCCTTCTCTACA
TCATTCTCTCTTCTCTCCCTATATAAACACAGAGACTTCCCCTATTCTTTTTCCTTGAAAACTAATA
CTTGTTCAACACGCTTCTCTACCTCAGCCACAGCCAAAATGTCTCCCTCCGCCTTCCATAAACCC
TTGCAATATCCCCTCGCCCCTCGAGACCATACCGTCGTCGACAATTACTTCGGCCTACAAGTCC
CCGACCCTTACCGCTGGCTTGAAGATCCGGATTCAGAAGAGACGAAGGAGTTTGTCGAGAATC
AAGTCAAGTTAACTGATACATTGCTTCAAGAATGCGAGTTGAGAGACAATCTTAAGGAAAAAAT
CACAGAGCTTTTTGATTATCCGCGATATTATGCGCCATTTAAGCGTTGTGATAAGTATTTTTACTT
TCATAATTCTGGTCTCCAGGCTCAAAATGTTCTCTATGTTCAGGATAACTTGGATACGAAGGCGG
AGGTCCTACTTGATCCTAACCAACTTAGTGATGACGGAACAGTGTCTTTGAACACCTACTCTGTG
AGTGAGGATGGCAATTATCTTGCGTATGGGTTGAGTTCAAGTGGAAGTGACTGGGTGACAATTA
AAGTAATGAAAATTGAAGACCGGAAAGTTGAACCAGACACTTTATCCTGGGTGAAGTTCAGCG
GCATTACATGGACGCATGACAATAAAGGGTTTTTTTATAACCGTTATCCACCACCAAATGAAGG
ACAAGTATTGGATGCTGGAACTGAGACAAATTCAAACCTCAATCAAGAGTTGTATTATCATGTTC
TTGGTACTGACCAGTCCGAAGATATTTTGTGTTGGAAAGACCCTGAGAATGCAAAGTATTTCTTT
GGGGCTGATGTAACTGATGACGGGAAGTATCTTCTCCTGTATACTTCAGAGAGTTGTGAAGTGG
TGAACAAAGTTTACTATTTAGATTTGACCATGCTGTCTAATGGGCTTGAAGGTTTCCGTGGGGGA
AAGGAGCTGCTACCTTTCACGAAGCTTGTTGATAATTTTGACGCTTCGTATTCTACTGTTGCTAAT
GATGATACAGTTTTCACTTTTCGAACTAATAAGGGTGCCCCAAAATATAAGCTAGTTCGTGTTGA
CTTGAAGTCGCCAAGCATATGGACCGACGTGATTCCTGAATCTGAGAAGGATGTTCTTGAATTTT
CTCTTGCTGTTAACAGAAACCAACTGATTGTCTGTTACTTAAGTGACGTGAAACACGTATTGGAG
GTGAGAGATCTTGAGACTGGTTCTCTGCTTCATCAATTACCCCTAGACATTGGATCAATTGATGA
CATCTCTGCACAGCGAGAAGACAGTGTCATGTTTTTTAGGTTTACAAGCTTCCTATCCCCTGGCA
TAATTTATCAATGTGATTTGAAAACTGATGCTCCAGAAATGACGATATTCAGAGAAATTGTTGTC

CCTAAATTTGACCGTTCCGAGTTCGAGGTTGATCAGGTATTTGTGCCTAGCAAAGATGGTACTAA
GATACCAATGTTCATAGTGGCAAGAAAGGGACTTTCTTTGGATGGATCACACCCATGTCTGTTAT
ACGGTTATGGTGGGTTTAACATAAGCATCACACCATCGTTTGGTGTCAGTCGTATAGTTCTTTTG
AAGCACCTAGGTGCTATCGTCTGCATAGCTAATATCCGGGGTGGTGGTGAATATGGAGAAGAGT
GGCATAAGGCTGGATCCGTTGATAAGAAGCAAAACTGTTTCGATGACTTCATTTCTTCGGCTGA
GTTTCTTGTTTCTAATGGTTATACCCAGCCTAAGAAGTTGTGTATTGAAGGTGGAAGTAACGGTG
GGCTCCTTGTTGGAGCTTGTATCAATCAGAGACCAGACATTTTCGGCTGCGCTCTGGCACATGTT
GGTGTTATGGACATGCTTCGGTTCCATAAATTTACCATTGGTCATGCTTGGACATCGGATTATGG
TTGCTCCGATAAAGAGGAAGAATTCCGTTCCCTTATCAAGTACTCACCTCTGCATAACGTAAGG
AGGCCATGGGAACAGACGTCCAATCTTTTGTCACAATATCCTGCTACCATGTTATTGACGGCAG
ATCATGATGATCGTGTGGTGCCTCTGCACTCATTAAAATTGCTTGCTACAATGCAGCACGTTCTT
GGTACAAGTTTAGAGAACAGCCCTCAGAAAAACCCAATCATTGGTCGGATTGAGTGCAAGGCT
GGACATGGCGCCGGACGTCCAACACAGAAGCAGATTAATGAAGCCGCAGATCGGTACAGCTTT
ATGGCCAAAGTGGTGAATGCTTCTTGGATTGACTAA

>PhPCY1-Ctg3671.23 **(PhPCY5)**

ATGATGTCTTCATCTGTCTCCGCCTTCCACAAACCCCTAAGTTACCCACTCGCTCGACGCGACGA
TTCTGTCGTCGACGATTACTTTGGTGTTCAAGTTCCCGATCCTTACCGGTGGCTAGAAGATCCGG
AATCAGAAGAGACGAAGGAGTTTGTGGAGAAACAAGTGAAATTAACAAACTCAGTGCTACAAA
AATGCGACTTAAGGGACACTCTTAAACAGAAATTAACCGATGTTTTCGACTATCCACGCTACAAT
GCGCCTTTTAAACGTGGTAACAAATATTTTTACTTTCACAATTCCGGTCTTCAATCCCAGGATGTT
CTCTATTCTCAGGATAACTTGGATGAGAAGGCAGAGGTTCTTCTTGATCCGAATCAACTTAGTGA
TGACGGAACAGTGTGCTTGATCACGTATTCTGTGAGCGAGGATGCCAATTATCTCGCTTATCAG
CTGAGTACAAGCGGGAGTGACTGGGTGACGATCAAAGTAATGAGAACTGAAGACCGTAAAGTT
GAACCAGATACTTTATCATGGGTGAAATTTAGTCAAATTACATGGACACATGACAACAAAGGAT
TTTTTTATTGCCGCTTTCCACCACCAAATGAGGGAGAAATATCGGATGCCGGAACGGAAACAAA
TATAAACCTGAACCAAGAGTTGTGTTATCATTTTCTTGGTACCAACCAGTCCGATGATATTTTGTG
CTGGAAAGATTCTGAGAACCCAGAACATATGTTTGAGACTCAAGTAACTGATGATGGGAAGTAT
CTTCTCCTCTATACTGTAGACGGCTGTGAAGTGGGTAACAAAGTATACTATATAGATTTGGCAAC
CCTGGCTAGTGGGCTTGAAGTCTTCCGTGGGGGGAAAGAGTTGCTTCCTTTCACGAAACATGTT
GATAACTTTGATGCCTCGTATAAGGCTGTTGCTAATGATGATACTGTGTTCACCTTTCTAACTAAT
AAGGATGCGCCAAATAATAAGTTAGTTCGTGTTGACTTGAAGACTCCCAGTATATGGATTGACG
TGATTTCTCAATCTGAGAAGGATGTGTTGCAATCAGCCCTTCCTGTTAACATGAACCAATTGCTT
GTTTGTTACTTAAGCAACGTAAAACACGTTTTGGAAGTGAGAGATCTCGAGACTGGTTCTCTACT
GCATCACTTACCCCTTGACATTGGATCTGTTGATGACATTTCTGCAAAGCGGAAAGACGATTTCG
TGTTTTTTAGGTTTACAAGCTTCCTATCCTGTGGCAAAATTTATCAATGTGATTTGAAAGCTGATG
CTCCAGAGATGAAGGTTTTCAGAGAGATTGTTATCCCTAAATTTGATAATTCTGAGTTTGAGGTT
GATCAGGTATTTGTGCCAAGCAAAGATGGTACTAAGATACCAATGTTCATAGTTTCAAGAAAAG
GGATTTCTTTGGACGGATCACACCCATGCCTACTATACGCTTATGGTGGCTTTAACATAAGCATG
ACACCATCTTTTGATGTCAAACATATAGTTCTTTTAAAGCACCTTGGTGCTATATTCTGCTTAGCT
AATATCCGGGGTGGTGGTGAATATGGAGAAGAGTGGCATCAGGGAGGTTCACTTGCTAAGAAG
CAAAACTGTTTTGATGACTTCATTTCTGCAGCCGAATTTCTTGTTTCTAAAGGTTATACTCAGCCT
AAGAAGTTGTGTATTGAAGGTGAAAGTAATGGTGGGCTTCTTGTTGGAGCTTGTATCAATCAGA
GACCAGAACTTTTTGGCTGCGCGCTGGCGCATGCTGGTGTTATGGATATGCTTCGGTTCCATCAA
TTTACCATTGGTCATGCTTGGACATCAGACTATGGTTGCTCGGACAAAGAGGAAGAATTCCACT

GGCTTATAAAGTACTCACCGCTGCATAATGTGAGGAGGCCATGGGAACAGTCACCAGATCATTC
ATCACAATACCCTGCTACCATGTTATTCACGGCTGACCATGATGATCGTGTGGTGCCTTCACATA
CATTAAAGTTGCTTGCTACTATGCAGTATGTTCTGTGTTCAAGTTTGGAGAACAGCCCTCAGACA
AATCCAGTCATTGGTCGTATTGATTGTAAATCTGGACATGGATACGGACGTCCGACCCAGAAGC
TGATTGATGAAGCTGCAGATAGCTATAGCTTTATGGCGAAAGTCGTGAATTCCCCGTGGACTGA
TTAA

>PhPCY1-Ctg3671.6 **(PhPCY6)**

ATGGGCGTTGAAGTGTCGTTGAAGTATCCTAATGCACGTCGTGATGACTCTGTCGTAGATTACTA
CCACGGCGTTCCTATTTCCGATCCTTATCGATGGTTGGAAGAAAGGGATTCAGAAGAAACAAAA
GAATTCGTAAAAAGGCAAGCAGAAGTGACAGAATCTTTATTGAAAACGTGCGAGACAAGGGAG
AAATTGGGGAAGAGACTGAAACAAGTATTTGAGTTTGAAAGGTACAATGTTCCTTTTAAACGCG
GGGACTTTTATTTCTACTTCCACAACAAGGGCCTCCAACCTCTTGATGTCCTCTTTTTTCAGGATG
ATCTGAAATCAGAGCCGAAAGTACTACTCGATCCTAATGAACTTAGCAAGGACGGAACGGTGT
CCCTGGGCTTATGCTCAGTGAGCGAGGATGCTAAGTACTTGGCTTACGGTTTACGTACTAGCGG
AAGTGACTGGAACACCATCAAAGTCCTCTCCATTCAGGACCGTGTTTTCCTTCCTGACACGATAT
CTTGGGTCAAGTTCAGTAACGTTACATGGACTAATGACAATAAAGGCTTTTTTTACGGTCGTTTC
CATGCTCCTAATGGAGAGACGACTGATACTGGTAAAGAAACAAATATCGATCTGAACCAGGAG
TTATACTTCCATGTTCTCGGTACTCATCAGTCTGAAGATGTTCTATGCTGGAAAGATCCTCAGCAT
CCTAAGTACAGATTTTCTACCTGTTTAACTGATGATGGAAAGTATCTTCTGTTGGACATTGGAGA
AACCTGTGATTCCTTGAACAAACTCTATTACCTCGATCTTTCATCCCTGGATAACGCCCTTCAAA
GTGCTCGAGATGGAAACACCCTTCTTCCATTCACCAAACTCGTGGATGAACTAGACGCACTTTAT
CAATACATCGCAAATGATGGCACAATCTTCACGTTCCAAACCAACAAGAATGCTCCAAAATGTA
AGCTGGTTCGTGTCGACTTGAATAAACCTGATGATTGGACCGATGTTCTTCCTGAGTCCGAAAA
GGATGTGCTTGTATCAGCTGATGCAGTTAACCAGAACCAACTGATCACATGCTATTTGAGTGAC
GTGAAACACGTCCTCCATATTAGGGACCTACATACTGGCTCTTTGTTGCATCATTTGCCTTTAGA
CATTGGCACGGTTAGGATTAGTGCTAGAAGGAAAGACACCATGGCTTTTATATCATTCACTAGTT
TTTTGACCTTCAATATAACATATCAGTGTAACTTAGCGCCTGAGGTTCCCGATGTGAAGATCTTC
AGGGAAATTTCGGTTCCTAATTTTGATCGCTTCGAATTCATGACTGATCAGGTTTTTGTGACTAGT
AAAGACGGCACCAAGATTCCGATGTTCATAGTGGCGAAAAAGGATGTTAAATTGGATAAATCAA
ACCCTTGTCTGTTATATGCATATGGTGGATTTAATAGTAGTCTTACGCCACATTTTAGTGTCATTC
GTTCGATACTTCTGAAACACTTGGGTGCCGTCTTCTGCCTTGCTAATATTCGAGGTGGTGGCGAG
TACGGTGAAGAATGGCATAAGGCAGGGACCTTTGAAAGGAAACAGAACTGCTTTGATGACTTTA
TTGCCTGCGCTGAGTTTCTCGTCTCGAATGGTGGTTATACACAGCCGTCTAAATTGTGCATTGAA
GGTGCTAGCAATGGTGGCCTTCTTATTGGTGCTTGCATAAATCAGAGACCAGATCTCTTTGGTTG
CGCTTTGGCTCACGTTGGTGTCATGGACATGCTTCGGTTTCACAAATTTACGATAGGTCATGCTT
GGACCTCTGATTTTGGTTGTTCTGACAAGGAAGAAGATTTCCATTGGCTAATCAAGTATTCGCCT
CTACACAATGTTAGAAGACCATGGGAGCATTCTAGTGGCCGATACATACAGTACCCGGCAACC
ATGTTATTGACCGCTGATCACGACAACAGAGTCGTACCGCTGCACTCTTTCAAGTTGTTAGCAAC
TATGCAACACACTCTTTGCACAAGCTCGACTAGGAGCCCACAAGTCAACCCAATAGTTGTACGG
ATCGAGAGCAAGGCTGGTCACTGTTGTGGTCGTCCAATACAAAAAGAGATTGATGCGGCAGCC
GACAGATACAGCTTCATGGCTAAGATGATGGATGTGTCATGGATCAACTAA

>PhOLP1-Ctg4232.8 **(PhOLP1)**

ATGGAGAAATCTAGCAAGAATATGAACAAAATCATGGCTTCTTACTTGTTAGGAGTTGTAATATT
GTTTAGTTTTGTGTTAGTATCCTTGGCAACTCCAAAAACTCATTACCATGAATTTGTCGTTCAATC
AAGACCAGTGAAGAGGCTGTGCAACACGGTAAATACTATAACCGTGAATGGCCAATTACCTGG
ACCAACTTTGGTAGTTACCGAAGGAGATGGCCTTGTTATCACTGTTGTTAATAGAGCCAAATACA
ACGTCACAATTCATTGGCACGGAGTGCGACAAATGAGGACAGGTTGGGCAGATGGACCCGAAT
TCATTACCCAATGCCCTATTCGACCCGGCGAGAGTTACACATATCGGTTCAGGATTCAAGGACA
GGAAGGAACTCTATGGTGGCATGCACATAGCTCATGGCTAAGAGCAACGGTCTATGGCGCTCT
CATAATTCTTCCTAGGGAAGGGACTCCTTATCATTTCCCTAAGCCTAACCGTGAAACACCCGTTA
TACTTGGAGAGTGGTGGAACCGTGATCCAGTTCGAGTGATAACACAAGCAACAAGGACCGGTG
CAGCCCCAAATGTATCGGACGCTTACACTATCAATGGTCAACCCGGAGATCTCTACAAATGCTC
TAGCCAAGATACAACAATTATTCCAATTGACGTAGGGGAAACACACCTACTAAGAGTAATCAAC
GCGGCACTAAACCAAGAACTATTTTTCTCAATAGCGAACCACATATTTACCGTAGTAGGAGCAG
ACGCATCATATCTTAAACCCTTCACAACAAATGTCATAATGCTCGGTCCCGGCCAGACTACCGA
TGTATTAATCTCTGGTAACCAACCACCTGGTCGCTATTACGTGGCCGGGAAACCATACCAAAGT
GCTCAAAACGCTGCGTTCGATAACACTACTACAACTGCCATCCTAGAGTATAAATCAGCACCTT
GTCCTAAGAAAGGTGTGCCTAAGGCCACCCCAATTTTGCCACTAATGCCCGCCTTTAACGACAC
CAACGCCGTTACTTCTTTCACTAGCAAGCTACGCAGCCCAAGGCAGGCCGAGGTGCCCGCAGA
AGTAGACGAGGACTTACATTTTACCGTAGGGTTAGGACTTAATAATTGCCCACCAAACTTTAGTA
AAAACCAATGTCAAGGACCTAACGGTACTAGATTTACTGCAAGCATGAACAATGTATCATTCATT
TTACCGTCCAATTGGTCATTACTTCAAGCCCACAAATACGGAATTCCCGGTGTATTTACCACCGA
TTTCCCGGCATCACCTCCGGTTAAATTTGACTACACCGGGAACGTTAGTCGGTCTCTCTGGCAAC
CGACGACAGGAACTAAGTTGTATAGGCTTACGTATGGGTCTAAGGTACAAGTTGTTATACAAGG
GACTAATATTGTTACGGGAGAAAATCATCCTATGCATTTGCATGGTTATGATTTTTATATTATTGC
CGAGGGATTTGGTAACTTTAATCCTAGAGTTGATTATAAGAAGTTTAATCTTGTTGATCCTCCTAT
GAGAAATACTGTTCCGATTCCGGTTAATGGATGGGCTGTTATTCGATTCGTCGCAGATAATCCAG
GGGTATGGATCATGCATTGTCATTTGGACGTACACATAAAATGGGGACTTGCTATGGCTTTCCTA
GTGGAGGATGGAATTGGAGAGTTCGAGACTTTAGAGTCTCCACCAGAAGATTTACCAATATGTT
AG

>PhOLP1-Ctg5689.43 **（PhOLP2）**

ATGGCATTCAAAAAAACACTAGTACAAAGATTATTCAACCTGTCTAGATTCTCAACTCCGTCATT
AACCAGAAACCCACCCGGAAAGCCAAACCCGACTCAACCCAACCCGACTCGCTCCCGGTCCAG
ACCGCTCCACCAATCAACCGAAACCATGCGGCTGTTCCCTTTGAAAGGACACGCATTGACCGA
GACGTTAAGAGGAATGGACATTACTAAAGGGAGGCTAACTCTAGACGGACTGATCGCGCAACC
AACTGAAACGACGAGTGTTGGCGACATGTTTTCGACTAATGAGGTTAGGAAAGTGTTGAGGGC
GACACAAGTTGAGATGTTGAGACGGAAATTGAATGCTGTTGGGAAAGATTGGGTCAGTTATGG
GGAATTTGTTGCTGTTATTCAGGATTTTTGTGGTGGAAATAGAGAAATTAGCCGTCAATTTATCA
AATTGTTGGATGATTCTGGTCTTGTTATTGTTTTTGGGAGTTCTGTTTGTTTGCGTCCCAGTCAGAT
AGCGAAAGTCATTCATAGCCTAGTCACACCACCCGTGTCGACGGACCCCAATGACCCGAGGAG
GAAGGAGTTGAAGGCGATGGAGGAGCAGAAAGCTGAGATTGACCTAAAAGCCGAGGCATTGG
TCCGTCGAGAATTGTGGGCCGGATTAGGGTACATGGTGGTCCAAACCGCGGCCTTTATGAGGCT
TACATTTTGGGAACTTTCATGGGATGTGATGGAGCCCATTTGCTTCTATGTGACTTCGGCGTATTT
CATGATTGGTTATACATTCTTTCTTAGAACATCGAAAGAACCTACTTTCGAAGGGTTCTACCTAA
GCCGCTTCAATGCAAAGCAGAAGCGGCTTATTGAGGCTCAAGAGTTTGATCTCGAGAGGTATAA

TGAGCTTCGGAGAATGTTTTACCCCGATTGTTCTTCGTCATTGGATGATGCTAGGTTGATCAAGT
ATGATCATTACCATAAGGTTAATTAG
>PhOLP1-Ctg1292.81
ATGGCGGAGAAATTAGAGCCGTTAATTAGTGTAAAAGCTTATTTATTAAAAGATGGATTAAATTG
GTGGAATGATATTAATAAATCCCTAATTTGGCAAGATCGAATTTTTCATGTTTTAGCTCTTTTCTAT
GGAATTGTTTCTTTTGTTGCTTTGGTCCAATTGGTGAGAATACAGCTCAGAGTTCCGGACTATGG
ATGGACTACACAGAAGGTTTTTCATTTTCTAAATTTCCTGGTTAATGGGGTTAGGTGCGTAGTGTT
CGTATTTCGTCGTAATGTGCAACACTTGCATCCTGAGATTGTCCAACATGTCTTGCTTGATATGCC
AAGTCTTGCTTTCTTCACTACCTACGCGCTTTTGGTTTTGTTTTGGGCAGAGATATACTATCAGGC
GCGTGCTGTATCAACTGATGGTCTCAGGCCTAGTTTCTATACAATTAATGCAGTGGTGTACATTA
TCCAGACTGTTTTGTGGTTGATCTTGTGGTGGAAGCCAATCCATGTTCTGCTTGTTTTGTCAAAGA
TGTTCTTTGCAGGCGTGTCTTTGTTTGCAGCTCTTGGATTTCTTCTTTATGGCGGAAGGCTTTTCAT
AATGTTGCAACGGTTTCCAGTAGAATCAAAGGGGCGGCGTAAGAAGCTTCAAGAGGTTGGCTA
TGTCACCACGATATGTTTCACATGCTTCCTCATAAGATGTGTTATGGTAAGTGTTGCATATCTTTC
AAGCCACCCTACTTGA
>PhOLP1-Ctg1292.47
ATGACTCCTGATGATCAACATATTTTACTTCTGGAGGACACTCTTCTGCAGAGAGTGAACAATAC
GAATGAACCGTGTACTGGAGATGGTTCTGTTGATTTCCATGGGCAGCCTGTCCTTAGGAGCAAA
ACTGGAAACTGGAAAGCCTGTCAATTCATTCTCGGTACTGAATGCTGTGAGCGTCTGGCCTTTCA
CGGGTTGGATACAAACCTCGTCAATTATCTCACAGGAAAGTTGCACGAAGGAAACGTCTCTGCT
GCTAGAATTGTTACTACATGGACAGGCACGTGTTCCCTTACACCTCTTATTGGAGCCTTCCTAGC
TGATGCATATTGGGGAAAATATTGGACCATTGCTGTTTTCTCCATGACTTACTTCATCGGAATGT
GCATACTGATACTTTCCGCTTCAGTTCCTGCACTTAAGCCTGCTGAATGCACGGATTCAGGTTGC
CCATCGGCTACTCCAGGACAATACGCAATATTATTCACAGGGCTTTACTTAACAGCACTAGGGT
CAGGAGGGATTAAGCCCTGTGTACCGGCGTTTGGTGCGGATCAGTTTGATGAGACTGACCCTGA
TGAAAGGGTAGATAAAGTATCTTTTTTCAATTGGTATTATTTCTTTGTTAGCGCTGGTTCTCTTTTA
GCTAGCACTCTAGTTGTTTGGATACAAGAAAATGTCGGATGGGGTATAGGCTTCAGTGTACCTG
CATTTTTTATGGGCATTGCTATCGCCAGTTTCTTCTCTGGGACTCATCTGTATAGGCTTCAAAGAC
CCGCTGGAAGCCCATTAACGAGAATATACCATGACATAATTGCATCAATCCGTAAGGACAAATT
AGAGGTTCCAAATGAGAGCAAAATACTGTGTGAAACACCAGAAAAGAATTCCCCGATTGAAGG
AAGTCTGAAGATGGACCAGAGTGAGGTTTTAAAATCGCGTGATATAGTAGCTGTTCCATCAGAT
GCTCAGATGAATCTCGGTGACATCGACAACCCAAAGAAGGTGTGTACAGCAACTCATGTAAAT
GAACTGACCATCTTGATAAGAATGTTCCCTATATGGATAACCGGGATAGTCTATTCCGCTGTTTA
TTCACAGACATCGACAATGTTTGTTGAGCAAGGCATGGTCATGAACCGTACAGTCGGATCATTC
ACAATCCCAGCTGCCTCCCTATCAATGTTCATTATGATCACCATAATGTCTTGGGTACCAATTTAC
GACAAACTTCTGATGCCCTTAGCTAGAAAGTTCACTGGCAACGAAAAAGGCGTCACAGAACTA
CAACGAATAGGCATCGGCTTATTTCTCTCCATCCTTCCTATGTTATACGCTGCTTTTCTGGAAATC
AAACGCTTGAAGCTTGCAGAGGAACACGGGTTAGTTGACAAAAATACCGCTGTTCCTTTGAGCA
TTACGTGGCAAATTCCACAGTATCTGTTAATGGGCATTGCTGATGGAATTACATATGTCGGAAAG
CTGGAGTTTTTCTATGATCAGTCAGGCGATAACATGCGCAGTTTGTCTACTGCTTTCTGTGTGCTA
ATCACCGCCATGGGGAATTACTTAAGCTCTCTGATTCTAACTATAGTGACAGAAGTTACGACTTC
TGGGGGAAATCTCGGGTGGATACCGGATAATCTGAACAAGGGACAACTTCACAAGTACTTCTTC
CTATGGGCAAGTCTCAGCTTTGTGAACTTGTTGCTCTTTGTTTTCTTTGCCATCAGGTATAAAGAC
AAGAAAATTTCATAG

>PhOLP1-Ctg4572.95
ATGAATAGAGGTGGTGTAAACTCAAGACGGCGACATAAACCTGTTGTTGATTCTCCTCCTAATC
ATCACCAAACCCCTGTACAAGCTGATGCCTCTTCTGGTGGTATTGTCGCAAGTGCTTCTGGTGAA
AAATCACATAGTTCAGGAAAAACAGTGGGACATACTTCTGGAGGTAGTGTTTATCACAATGAAA
CTCTGGCTGGTCGCCAGGGACGACGATGGTTTACATGTAGCAGTGGGAGTAGCTCTCGTGGAA
GTAGTATTTATCACAATGAAACACCGGCTGGCTGCCAGGGACGGGGATGGTCTACATATGGCA
GTGGGAGTAGCTCTCGTGGAGGTAGCACAAGACAATGGACTAAATCTTCCAAGGCTAGCAATG
TTTCTGATTTCCACCGTGATGAGCTTTTTGGATCGAATTCTCGTGAGAAAGATGTCAACAAACAA
TCAAGTGATACTCTAAAAGCTTCGCGTGAAGGAGATAGTGAATCTTTGCCTATAAATCTTATTGG
CACATTCACGGAATGTCATGTCCATCACCCTGATGCTGCCCAATTGTCTGGGGAGTCTATGCTTC
CAGAAAGTATTGCCAGTATACGAGAAAATGAAAATTCTGAATGCCGCACAGTGAAATTTGATCT
CTGCCCGCCTCGAAAATCAAATTTGGTCAAATTGAACCCACCCTTGATGTTAAAAAACAAAGAA
AGGCGTAAAGAAAATGCACGGTCCGTGGATGGGCCAAAAGGAGAAGTATTGAGACCTGGGAT
GGTCCTGCTGAAAAGCTATCTTTCAACTTCTGAACAGACCAGTATTTTAAACATTTGCCGAAACC
ATGGTCTTGAAACGGTAGGTTTCTGCCAGCCGGAATTTGGTGATGGAGGGAAGATGCATCTGAA
GTTGATGTGTTTTGGAATGTTTTGGAATCCTGAGACTAACCAATATGGGGATGTTCGATCTGATG
GTGTAAAACCACCACCTATTCCTCAGAAATTTCTGGAGTTGGTTGATAGAGCAATTAAGGACTCT
AGGTCTCTTATTTCTGAGGATCCCATGGTTAAAAATGTGGAGTCCGTACTTCCGTCAGTCAAGCC
TGACATATGCCTGGTTAATTATTATTCGAATACAGGACGTCTTGGGCTACATCAGGACAAAGAC
GAGAGTCGAGAAAGTCTCATTAAAGGAATTCCGATTGTATCTTTCTCCATCGGCGACTCTGCAG
AATTCGTATACAGTGATAAGAGAGATGTTGAAAACGCAGACCAAGTGACACTAGAATCAGGAG
ATGTCTTGATTTTTGGTGGAGATTCTAGAAACATATTTCATGGTGTTCCCGCAATTAAAAAAGAA
ACCGCGCCTAAACAGTTGCTTGCAGAAACAAACTTCCGCTCTGGTCGTTTGAATCTAACCTTCCG
ACAGCGTTTCTAG
>PhOLP1-Ctg1928.146
ATGTTTGAGGCTGCTTTGAAAGATTGCAAATATGTGTTTCATCTTGCCACACCTTATTTGCATACC
CACAACTCGAAATTTAAGGACACGACAGAGGCAGCAGTTGAAGGGTTAAAGAGTATTGCTATG
TCCTGTATTCAATCAGGGACCGTGACACGGCTAATCTATACCGCTAGTGTAACATCTGCTTCACC
TCTCAACAATGACAGTACTTCTTTCGCGGAGCTCATGGATGAGGATTGTTGGACTCCTCTTACGT
TCCCATTTAGCTTCGCACGAGAAAATGAAATGCATTATGTGAAGTCGAAAACAGAATCAGAGAG
GGAGGTACTGAAACTGGGAGATGACGGAAATTTGGAGGTGGTAAGTCTAGCATGCGGGCTGGT
AGGAGGGGAGACGGTTCTCCCTTATGTACCGGTTTCTGTGTTAGTTTTAGTATCTCAGTTAAAAC
AGGAGGAATCTGTAGCATATCAACAATTGAGATTCATAGAAGAACTATGTGGAAAGATTCCTAT
TATACATATTGAAGATACTTGTGATGCTCATATATTCTGCATTGAGAAAGATTCTATCAAAGGGA
GATTCTTATGTGCCTCGAACTTTGTTTCCTCGACTGAAATCGCGGAGTATTATAAGGAGACATAC
CCGGAATTCCCTGTCAAACTAGTGTGCACAGATGTGCCGAGGAGGAATGTGAAGTGGGGATCG
ACAAAGCTAATAGATGAAGGATTCGAGTATAAGTGTGACTTGAAGATGATATTGGATGATTCTG
TTCAATGTGCTCGGAGGTTTGATGTGATTTAG
>PhOLP1-Ctg4572.88
ATGCAAAATCAACGATTTAAGCAGCAGCAGCAGCAACAACAAGAAGCATTAATGCAACAAGCT
GCTCTTCTTCAACAACAATCTATCTATCATCCTGGTCTTCTCGCTCCTCCTCAGATTGAGCCGTAC
CCTAGTGGCAATCTTCCTCCTGGCTTTGATCCCTCTTCATGCCGCAGTGTGTATGTGGGCAATGT
TCATCCTCAAGTTACAGAACCATTGCTCCAAGAGGTTTTCGCTAGTGCCGGGCCTGTTGAAAGTT
GCAAACTCATTAGGAAGGAACAGTCATCCTATGGCTTTGTTCACTACTATGATCGCAGATTTGCT

AGTATGGCTATAATGACACTTAGTGGGAGGCATTTGTTTGGGCAGCCCATTAAGGTTAATTGGG
CGTATACTAGCGGTCAGCGTGAGGATACTTCAAACCATTATAATGTGTTCGTTGGCGATCTGAG
CCCTGAGGTTACTGATGCTATGCTGTTTGCTTGTTTTTCTGTTTATCCTACTTGTTCAGATGCCAGA
GTTATGTGGGATCAGAAAACTGGACGTTCAAGGGGGTTTGGATTTGTGTCTTTCCGCGATCAGC
AGGAGGCCCAAGGTGCAATAAATGATCTTACTGGAAAATGGCTTGGCAGTAGACAAATAAGAT
GCAATTGGGCAACTAAGGGAGCTACTAGCGGTGATGATAAACAGATTACTGATGCTAAAAGCG
TGGTAGAATTGACAAATGGCTCATCAGAAGATGGAAAGGAGACTACAAACAGCGAGGCACCTG
AAAACAATCCTCAATATACAACAGTATATGTTGGAAATCTCGCTCCCGAGATAACCCAGCTTGA
TCTGCACCGCCACTTTCATCAGTTAGGTGCCGGGGCAATTGAAGAAGTAAGGGTGCAGCGTGA
CAAGGGGTTCGGTTTTGTCAGATACACAACACATGGTGAGGCTGCCTTGGCTATTCAGTTTGGT
AATAATCCCCAGAATGTGCTTTTTGGCAAACAAATTAAGTGTTCATGGGGTAGCAAGCCGACAC
CTCCTGGCACGGTCTCAAATCCACTCCCACCTCCTGTGGCACCTTTTGGATTTTCGAATACAGAT
CTCCTCTCCTATGAGAGGCAACTAGCAATGAGCAAAATGGGTGGAATGCATGCACTCATGAAC
GCACAAGGAGCACACCCTTTCAACAAGGCTAATATGGGAATGACGGCCGCTGGAGCCAGCCAA
GCTATTTATGACGGCGGGTTTCAGAATCTTGCAGCTGCCCAGCAAATGATGTACTACCATTAA
>PhOLP1-Ctg4976.20
ATGGCGGTGGTATCACCATTAGCGAAATATAAACTTGTATTTCTCGGCGATCAATCCGTCGGTAA
AACTAGCATTATTACACGTTTCATGTACGATAAATTCGACACCACTTATCAGGCTACAATTGGTA
TAGACTTCTTGTCAAAGACTATGTATCTTGAAGATCGAACAGTACGGTTGCAACTTTGGGATACT
GCTGGACAGGAAAGGTTTCGGAGTTTGATTCCTAGCTACATCAGAGATTCTTCAGTTGCTGTAAT
TGTTTATGACGTTGCTACTAGGCAGTCATTTTTGAACACGTCGAAGTGGATTGAGGAAGTACGA
ACAGAACGTGGCAGTGATGTGATCATTGTTCTTGTCGGGAACAAAACTGATCTAGTTGAAAAAA
GGCAAGTCTCTATAGAGGAAGGAGATAGCAAGGCCCGTGAATCTGGGGTGATGTTCATTGAGA
CTAGTGCAAAAGCAGGATTCAACATCAAGCCTTTATTTCGTAAGATTGCCTCGGCATTGCCAGG
GATGGAAACTCTTTCATCAACAAAACAAGAGGACATGGTGGATGTGAACTTGAAGCCCCACAG
TAACTCATACCAATCAGAACAACCGGGAGGTGGTTGTATGTGCTAA
>PhOLP1-Ctg4732.2
ATGGTAAATGAAGAAGATAATCCAATATTGTGGGATTTCACTTTCCCCCCTTATCATCTCATCCA
ACCCAACCATATTATCTTTGGGATTCCATCTCTCTTTACCCAACTCGAAGTAGAGTTAGCTGAAC
TAGAGAACAAGGTTGAGCCAAAATGGGGTAAGTTGGTAGTTCCATTGGAGAGAATTGCAGATA
GATTAAATGTGGTGTGGGGTGTTGTTGATCATCTTTGTTCCGTTAAGGATTCGCCTCAACTTCGTG
CTGCGGTTGAGCTTGTTCAGCCACAAAAAGTAGCTTTTCAGCTTAGATTGAGCCAGAGCAAAAG
AATTTACAAAGCGTTCAAAGAAATTCGTGAATCTTCAGATTGGGACACTCTAAGCAGCTCCCAT
AAACGTGTGGTTGAAGGTCAATTGAAGATAGCAATTCTCGGCGGTGTCTCTCTTGAAGGTCATG
AAAGAGAGAGATTTAATGAAATTCAGCAGGAGTTGGCAAAACTAGCCCTGAAATTTGACGCCA
ATGTTCTAGATGCCACTAAAAATTATGAGAAACTTATCACTGACAGAGTGTATGTTGAAGGTTTG
CCAGCCACAACACTTGCTTTGGCAGCAAAGGCAGCGTCATCTAAGGGCCATGCGGAGGCTACA
GCTGAAAATGGACCATGGGTTATCACATTGGATCCTCCAATCTATCGCTCAGTAATGCAGCATG
CCAAGAAGAGAGAGTTACGTGAGGAAGTTTACCGTGCCTACATCTCACTTGCTTCTGCAGGTGA
TCTTAATAATACACCAATAATAGAGGAAATTCTTAAGCTTAGGTTGGAGAAAGCTAAGCTTCTTG
GTTACAGCAACTACGCTGAGGTGAGCATGGTGACAAAAATGGCCACTGTTGATCAGGCCAAAG
AACTAATAGAAACACTTCGTGTTTCATCGTGGGATGCTGCAGTTCATGAACTGGAAGATCTCAA
AAGTTTTGCTGGATCTAAAGGTGCTGTGGAAGCCACTAATATAAACCATTGGGACATCAACTTCT
GGAGTGAGAGAATACGTGAATCGAGCTATGACATCAAGGAGGAAGAATTGCGTCCTTATTTTTC

ACTCTCTAATGTAATGGATGGCCTTTTTTCACTAACAAAGATGCTCTTTGATGTTGAGATTTTCGC
TGCAGATGGGGAAGCTCCCGTGTGGAATGATGATGTAAGGTTCTACTGTGTCAAGAATCCTAAG
GGGAATCCAATCGCCTACTTCTATTTAGATCCTTATTCTCGGCCATCAGAAAAGCGAGGTGGGG
CATGGATGTCTCTTGTTGCTGGTTGTAGTCGTTCTCTTTCTCGTGATGGTACATCATGTAGACTAC
CTATTGTTAATGTGGTGTGCAATCAAACGCCTCCACTGGGAGACAAGCCAAGCCTAATGACGTT
TCGTGAGGTTGAGACTATCTTCCATGAGTTTGGGCATGCCCTTCAAATTATGTTAACCAAAGAAG
AAGAGGTTTTTGTTTCCGGTAATCGGGGTATAGAGTGGGATGCAGTTGAGTTGCCTTCATCATTC
ATGGAAAATTGGTGTTACCAAAAGTCCTTGTTGACAAACATTGCCAAACACTATGAAACGGGGG
AGCATCTGTCAGAAGAAGTCTGTACCAAACTTCAATCTACAAAGACTTTCCGTGCTGCTTCAACG
ATGCTTCGTCAGATGCGATATGCAGGTGTTGATCTTGAGCTACACTCGGAGTATATTCCCAGAG
GGACAGAATCAATTTATGATGTTGATCAAAGAATAGGAAGAAGAACAAGTATTATCCCTCTTCTC
CCTGAAGACAAATTTCTTTGTATTTTCAGTCATATTTTTGCAGATGATTATGCTGCCGGATACTAC
AGCTATCAGTGGGCAGAAGTGATGTCTTATGACGCATTTTCAGCATTTGAGGAGGCTGGGCTGG
ATAATCCGAAGGCTCTCAAAGAAATCGGTCACAAGTTTCGAGACACAATACTCGCTTTAGGCGG
AGGAAAATCACCCTTGGATGTATTCGTGGAGTTCCGAGGGCGTGAACCTTCACCACGGGCATTT
TTACGGCACCTTGGAATGTCACCAGCAGCTAGTACCTAA
>PhCP-Ctg3970.46
ATGGATGCCAATCATATGATACTAACAATACTATTAATCTCCTGCATCGCAACTCAATCCTTATC
GTCGACCTTCAATGACGAAAATCCAATCAGATTAATATCCGACGGTCTACATCACATCCAATCTT
CCGTTCTTTCTGCTCTCGGCCACACTCGCCACTCCCTCTCTTTTGCTCGTTTTATTCACAGGCATG
GAAAGAGGTATGAAAGTGTGGGAGAAATGAAGATGAGATTTGAAGAATATAAGAAGAGTTTGG
ATTTGATTAGATCAACTAATTCACAAAACTTGCCTTACAAACTTCATCTTAATAAGTATGCAGATT
GGACGTGGGAAGAGTTCCGTTCGCACCAATTAGGTGCTGCTCAAAACTGCTCCGCAACTCTCAA
GGGTAGTCACAAGTTGTCTCATGTGACTGCTCCTGAAGAGAAAGATTGGAGAGCTGAGGGTATA
GTGAGCCCAGTCAAGAATCAAGGTCACTGTGGATCTTGCTGGACATTCAGTACCACTGGTGCTC
TGGAAGCAGCTTATGCACAGGCACATGGGAAGAACATCTCTTTGTCCGAGCAACAACTCGTGG
ATTGTGCTGGCTCATTCAACAACTTTGGTTGCAATGGTGGTTTGCCATCACAGGCATTTGAGTAC
ATCAAGTTCAATGGGGGTCTAGACACTGAAGAGGCTTATCCTTATGTAGGGAAGAACGGCCTAT
GCCGTTTCTCGTCTCAGAACATCGGTGTTCAAGTTCTTAACTCAGTTAACATCACTCTGGTAAGTT
ATCTAATCATTCGTCAATGTGACTTGCTGGTGCAAATCTAA
>PhSP-Ctg4925.20
ATGCAGTTTGTGAATGCTCAAGAAAATGGAGAAGTAGAAAGAAAAACAGCACGTCCATTTCTCG
ACAAGATGTACGTTTTCTACCACTTAAATCAAATGAAAGAAGGAAATATATTCCCATTTTACTTTG
GAAGTCCATTTTCTACACAATACCCTAATTTTCTTCCTAAGGAAAAAGCCGATAAAATTCCCTTCT
CAACTACTAAACTCCCTTATATACTCGAATATTTCGGCCTTGGCCAGGGTTCGAAACAAGCCAA
GGCCATAGAATATGTACTCAAACATTGCGAGTACGAAAAAAAACACAAAGTAATCAAATTTTGT
TCTACCTCCTATGAGTCGATGATTGAAAATGTGTGGAAAAATGTTGGACCAAATACAAAATCAAT
TAAACCATTAAGTACTAGTCCACTTCCGAAAACAACCAAAAAATTACAAAATTATAAAGTAATTA
GTGAACCAAAAGAAGTGGAAGGAAGTGAAAATATGTTGGGATGTCACTTAATGACATACCCTTA
TGCCGTATACTATTGTCATCGACCGATCGGTACTACTAAAGTTTTTGAGATTCAATTAATAGGCG
ACGATGGTACCAAAATGCATTCGATTGCTACTTGTCATTTGGATACTTTGAAATGGGATACAAGT
CACACTGCGTTTCGGGTTTTGGGGACTAAACCGGGTGAACCCGTTTGCCATGTCCATTCACCGG
ATGATCTTGTTTGGGTGCCTGTTGTTGATAGTTCTTCTCATGTTATGTAA
