# Supplementary material for: Identification of a key peptide cyclase for novel cyclic peptide discovery in Pseudostellaria heterophylla
Source: Plant Commun. 2025 Mar 13;6(5):101315. doi: 10.1016/j.xplc.2025.101315 (PMC12143145; doi:10.1016/j.xplc.2025.101315)
Supplement: Document S2. Article plus supplemental information [file mmc4.pdf]

Plant Communications
Research article

CellPress Partner Journal

Identification of a key peptide cyclase for novel cyclic peptide discovery in *Pseudostellaria heterophylla*

Xianjin Qin[1], Fengjiao Wang[1], Dejin Xie[1], Qi Zhou[1], Sheng Lin[2], Wenxiong Lin[2,*] and Wei Li[1,3,*]

[1]Shenzhen Branch, Guangdong Laboratory of Lingnan Modern Agriculture, Key Laboratory of Synthetic Biology, Ministry of Agriculture and Rural Affairs, Agricultural Genomics Institute at Shenzhen, Chinese Academy of Agricultural Sciences, Shenzhen 518000, China

[2]Fujian Provincial Key Laboratory of Agroecological Processing and Safety Monitoring, College of Juncao Science and Ecology, Fujian Agriculture and Forestry University, Fuzhou 350002, China

[3]Bama Yao Autonomous County Rural Revitalization Research Institute, Bama 547500, China

***Correspondence: Wenxiong Lin (lwx@fafu.edu.cn), Wei Li (liwei11@caas.cn)**

https://doi.org/10.1016/j.xplc.2025.101315

ABSTRACT

Orbitides, also known as Caryophyllaceae-type cyclic peptides, from the Traditional Chinese Medicine plant *Pseudostellaria heterophylla* (Miq.) Pax, exhibit great potential for improving memory and treating diabetes. Orbitides are ribosomally encoded and post-translationally modified peptides; however, the key biosynthetic enzyme mediating this process remains unknown in *P. heterophylla*. In this study, we investigated the distribution of orbitides in *P. heterophylla* and mined novel precursor peptide genes and peptide cyclases from multiple omics datasets. The function of *PhPCY3*, a gene encoding a key tailoring enzyme, was elucidated using transient heterologous expression and virus-induced gene silencing systems. Our findings suggest that PhPCY3 specifically cyclizes linear precursor peptides *in planta*. Molecular docking and multiple sequence alignment, followed by site-directed mutagenesis, identified N500 and S502 as critical amino acid residues for PhPCY3 function. We identified gene sequences for over 100 precursor peptides and successfully biosynthesized known active orbitides, such as heterophyllin B and pseudostellarin E/F/G. Additionally, four novel orbitides, cyclo-[LDGPPPYF], cyclo-[WGSSTPHT], cyclo-[GLPIGAPWG], and cyclo-[FGDVGPVI], were synthesized using a heterologous expression platform. This study introduces a gene-guided approach for elucidating the biosynthesis pathway and discovering novel orbitides, providing a strategy for mining and biosynthesizing novel orbitides in *P. heterophylla* and other plants to further investigate their activities.

Key words: orbitides, tailoring gene, RiPPs, VIGS, synthetic biology, *Pseudostellaria heterophylla*, heterologous expression

Qin X., Wang F., Xie D., Zhou Q., Lin S., Lin W., and Li W. (2025). Identification of a key peptide cyclase for novel cyclic peptide discovery in *Pseudostellaria heterophylla*. Plant Comm. **6**, 101315.

INTRODUCTION

Pseudostellaria heterophylla (Miq.) Pax (family Caryophyllaceae) is utilized in Chinese herbal medicine and possess significant pharmacodynamic value (Hua et al., 2016; Wu et al., 2019; Yang et al., 2020; Xiao et al., 2022). Its roots are traditionally used to treat spleen deficiency, anorexia, post-illness weakness, and spontaneous perspiration (Qin et al., 2017). It is the main component of several commercial products, including indigestion tablets (Ma et al., 2023) and liver rehabilitation pills, generating hundreds of millions of dollars in economic value.

Specifically, heterophyllin B (HB), extracted from the roots of cultivated *P. heterophylla*, is a key quality control indicator required by the Chinese Pharmacopoeia (Commission, 2020). This compound enhances cognitive function through neurite outgrowth and synaptic plasticity and alleviates amyloid-β-induced memory deficits (Yang et al., 2021; Deng et al., 2022). Moreover, HB and its derivatives act as dipeptidyl peptidase IV inhibitors and GLP-1 receptor agonists which have been considered therapeutic alternatives for treating type 2 diabetes (Liao and Tzen, 2022a, 2022b).

Cyclic peptide drugs exhibit valuable pharmacological characteristics, including stable conformation, strong affinity, high metabolic stability, and high oral bioavailability (Muttenthaler et al., 2021; Li et al., 2022; Zhang and Chen, 2022). Drugs based on cyclic peptides, including those with anticancer and antibiotic

© 2025 The Author(s). Published by Elsevier Inc. on behalf of CAS Center for Excellence in Molecular Plant Sciences, Chinese Academy of Sciences, and Chinese Society for Plant Biology.
This is an open access article under the CC BY license (http://creativecommons.org/licenses/by/4.0/).

properties and those used to treat obesity, such as lanreotide and setmelanotide, generate billions of dollars in global sales (Caplin et al., 2014; Ryan, 2020; Zhang and Chen, 2022).

Plant cyclopeptides (CPs) are ribosomally synthesized and post-translationally modified peptides (RiPPs) containing 4–37 amino acids and exhibiting diverse chemical structures (Tan and Zhou, 2006; de Veer et al., 2019; Daly and Wilson, 2021; Chekan et al., 2024). A distinctive characteristic of plant CPs is their ability to undergo macrocyclization, which is typically categorized into three forms: head-to-tail, side-chain-to-side-chain, and side-chain-to-backbone (Kersten and Weng, 2018; de Veer et al., 2019; Slazak et al., 2020; Montalbán-López et al., 2021; Chekan et al., 2024; Kandy et al., 2025). Head-to-tail cyclized CPs, including orbitides and cyclotides, exhibit diverse sequences and bioactivities (Tan and Zhou, 2006; Montalbán-López et al., 2021).

Orbitides containing 5–16 residues and lacking disulfide bonds or non-natural amino acids were originally referred to as Caryophyllaceae-type CPs (Fisher et al., 2019, 2020; Daly and Wilson, 2021) and have demonstrated potential anticancer or antitumor properties (Daly and Wilson, 2021; Tehrani et al., 2021). Evolidine (cyclo-[SFLPVNL]), an orbitide, was the first isolated plant cyclic peptide, and its sequence was confirmed by Fisher et al. (2020). An increasing number of orbitides have been discovered in *P*. *heterophylla*, *Vaccaria segetalis*, and *Stellaria dichotoma* (Morita et al., 1994; Tan and Zhou, 2006; Zhao et al., 2020; Dahiya et al., 2021). Several orbitides, including heterophyllin A–H/J and pseudostellarin A–H/K, have been isolated and purified using traditional phytochemical methods (Tan and Zhou, 2006; Zhao et al., 2020); however, it is unclear whether additional low-abundance orbitides remain undiscovered in *P. heterophylla* using traditional methods.

The precursor peptide genes of CPs are translated by the ribosome as linear peptides and then modified by tailoring and cyclase enzymes (Condie et al., 2011; Barber et al., 2013; Craik and Malik, 2013; Chekan et al., 2017; Montalbán-López et al., 2021). However, the enzymes involved in the biosynthesis of plant cyclic peptides are not conserved across different CPs and do not belong to the same protein family (Chekan et al., 2017; Daly and Wilson, 2021; Kersten et al., 2022). Asparaginyl endoprotease (AEP), a cysteine protease, cleaves Asn or Asp-Xaa bonds to generate an acyl-enzyme intermediate and cyclizes the intermediate into SFTI-like CPs (Bernath-Levin et al., 2015; James et al., 2018; Hemu et al., 2019). Crude oligopeptidase 1 (OLP1) cleaves the linear peptide at the N-terminus to produce a linear intermediate (presegetalin A1[14-32]), which contains an N-terminal glycine (Barber et al., 2013; Chekan et al., 2017). Subsequently, peptide cyclase 1 (PCY1), a serine protease of the S9 family, removes the C-terminal flanking sequence and cyclizes core peptide residues, forming segetalin A (Barber et al., 2013; Chekan et al., 2017). Heterophyllin B, encoded by the *PhPreHB* gene, can be synthesized with crude enzymes in phloem (Zheng et al., 2019), but the role of OLP1 and PCY1 in cyclization is still unclear.

Here, we employed multiple omics datasets to target precursor peptide and tailoring genes. More than 100 precursor peptide genes were identified in the transcriptome, and *PCY1* and *OLP1* were discovered in the genome and transcriptome through Pearson correlation analysis. The key tailoring gene for orbitide formation, *PhPCY3,* was verified using *Nicotiana benthamiana* (*N. benthamiana)* transient expression and *in vivo* virus-induced gene silencing (VIGS) system, and the functional roles of its key active site residues were subsequently examined. We also used this transient expression platform to identify and synthesize new orbitides from the transcriptome. This research not only facilitates the discovery of novel orbitides from the plant kingdom but also provides potential targets for CP drugs.

RESULTS

Tissue-specific orbitide accumulation in *P. heterophylla*

To determine orbitide accumulation patterns, we collected different *P. heterophylla* tissues (Figure 1A) and analyzed their orbitide content using liquid chromatography-mass spectrometry (LC-MS) (Figure 1B and 1C; Supplemental Figure 1). Total ion chromatograms (TICs) and extracted ion chromatograms (EICs) generated using a Q Exactive™ HFX Mass Spectrometer showed that HB and pseudostellarin E/F/G (PE/PF/PG) were present in the roots (Figure 1C; Supplemental Figure 1). The particle fragment information was confirmed through secondary mass spectrometry compared to the standards (Supplemental Figure 2). Subsequently, LC-MS (TSQ Quantum Access MAX) was used to quantify orbitide content across different tissues. HB and PE/PG contents were significantly higher in roots than in other tissues (Figure 1D). Additionally, HB content in roots reached approximately 38.4 μg/g (fresh weight [FW]), which was several times higher than PE (4.4 μg/g FW) and PF (7.6 μg/g FW) (Figure 1D).

Potential tailoring genes in the orbitide biosynthesis pathway

Cyclic peptides are thought to be synthesized from precursor peptide genes that encode linear precursor peptide sequences (Supplemental Figure 3). OLP1 and PCY1 may be involved in excising and cyclizing linear precursor peptides (Supplemental Figure 3). To analyze the gene expression patterns of candidate genes, RNA-sequencing was performed on the same tissues that were used to detect orbitides. *PhPCY1/2/3* and *PhOLP1/2* genes were highly expressed in roots (Figure 2A), whereas other homologs were primarily expressed in flowers or leaves (Figure 2A). Correlation analysis between cyclic peptide content and candidate gene expression showed that these five genes exhibited a strong positive correlation (Figure 2B and 2C), with *PhPCY1-Ctg883.50* (*PhPCY3*) and *PhPCY1-Ctg883.47 (PhPCY1)* showing Pearson correlation coefficients greater than 0.90 (Figure 2B).

Phylogenetic analysis of *PhPCY* genes and other known cyclase genes involved in cyclic peptide biosynthesis was performed. Specifically, *SvPCY1* could excise the C -terminus of presegetalin A1[14-32] and cyclize the N -terminus, resulting in the production of segetalin A (Chekan et al., 2017). The results showed that *PhPCY3/2/1* clustered with *SvPCY1*, the first cloned plant cyclase with a confirmed role in peptide cyclization (Barber et al., 2013; Chekan et al., 2017). *PhPCY3* was more closely related to *SvPCY1* than to other homologs (Supplemental Figure 4). In contrast, *PhPCY4*, *PhPCY5*, and *PhPCY6* formed a

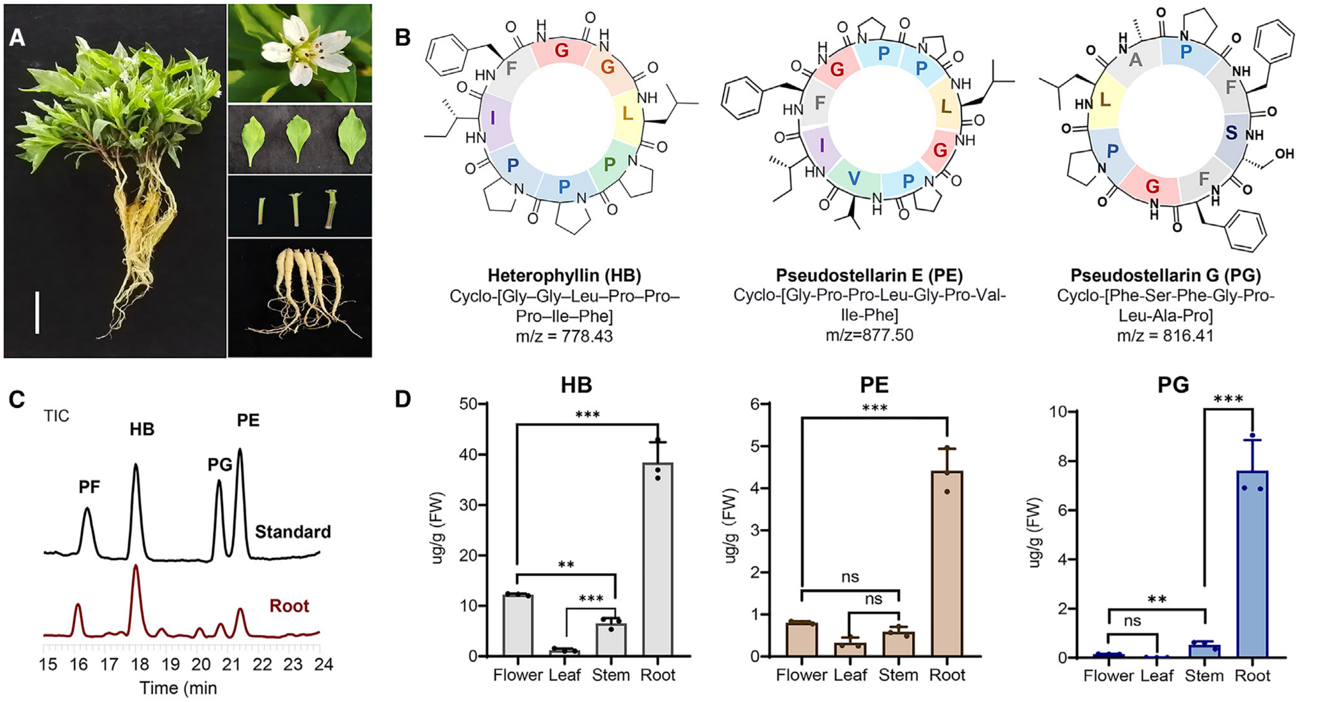

Figure 1. Tissue-specific orbitide accumulation in *P. heterophylla*.

(A) Whole plant and tissues sampled from *P. heterophylla in this study.* The scale bar represents 2 cm.

(B) Chemical structure of heterophyllin B and pseudostellarin E/G.

(C) Total ion chromatogram (TIC) of root extracts from the main cultivar "Zheseng No.2" from Fujian province, showing the known cyclopeptides, including heterophyllin B and pseudostellarin E/F/G, based on Q Exactive HFX LC-MS/MS.

(D) Quantified contents of heterophyllin B and pseudostellarin E/G in different tissues using LC-MS (TSQ Quantum Access MAX). The least significant difference method was used for statistical analysis after one-way ANOVA in (completely randomized design) in DPS software (n = 3). $^{**}p < 0.01$, $^{***}p < 0.001$, and “ns” refers to no statistical difference.

cluster with *BvPOP*, which encodes a serine protease belonging to the prolyl oligopeptidase subfamily. These results suggest *PhPCY3* is a strong candidate gene in the orbitide biosynthesis pathway.

PhPCY3 dominates orbitide cyclization

The *N. benthamiana* transient expression platform has been widely used to elucidate biosynthesis pathways (Kersten and Weng, 2018; Xu et al., 2019; Chigumba et al., 2022; De La Peña et al., 2023; Reed et al., 2023; Zhang et al., 2023; Jiang et al., 2024; Zhou et al., 2024). To identify enzymes responsible for CP cyclization, we constructed transient expression vectors for precursor peptide (*PhPreCPs*), *PhPCY1*, and *PhOLP1* genes in pEAQ-HT using homologous recombination (Supplemental Table 1). To narrow down the target genes, these genes were divided into three modules based on their potential functions (Table 1). A wide variety of *PhPreCPs*, which are essential substrates for orbitide synthesis, were placed in module 1. Modules 2 and 3 contained *PhOLP* and *PhPCY* genes, respectively (Table 1).

Different modules were co-infiltrated into *N. benthamiana* leaves with precursor peptide genes. HB was detected using two daughter ion fragments (m/z = 203.035 and 226.129) in selective reaction monitoring (SRM) mode. The *PhPCY* module included the core enzyme(s) responsible for the HB biosynthetic pathway (Figure 3A; Supplemental Figure 5A, 5C, and 5D). The minor peak detected in the PhOLPs module was not attributable to HB, as evidenced by the distinct MS2 fragment profiles (Figure 3A; Supplemental Figure 5B). Furthermore, chromatogram results showed that among PhPCYs, only PhPCY3 catalyzed the linear precursor peptide to HB, and it could function without the help of PhOLPs (Figure 3B; Supplemental Figure 6). Given that *PhOLP1* and *PhOLP2* had a strong positive correlation with orbitide accumulation and were specifically highly expressed in the roots (Figure 2B and 2C), we expressed *PhOLP1/2* in *N. benthamiana* leaves to confirm their activities. Neither could function independently in orbitide biosynthesis (Supplemental Figure 6). *PhPCY3* also cyclized PrePE, PrePF, and PrePG to PE, PF, and PG, respectively (Supplemental Figure 7). These results are highly consistent with the tissue-specific expression and evolutionary relationships of the *PhPCY3* gene (Figure 2B and 2C). Furthermore, an *in vitro* enzymatic activity assay was performed on PhPCY3 using two distinct substrates: HB [14-35], a linear peptide encompassing the IFGGLPPPSQELINGDDISLMV sequence, and HB [14-21], a truncated variant with the IFGGLPPP sequence. PhPCY3 excised the C-terminal region of HB [14-35] and cyclized the propeptide to form HB (Figure 3C and 3D; Supplemental Figures 8 and 9A). Additionally, the linear peptide HB [22-35] (SQELINGDDISLMV) was detected as a byproduct (Figure 3D; Supplemental Figure 9B). However, PhPCY3 was unable to directly catalyze the conversion of the shorter substrates, HB [14-21], into HB

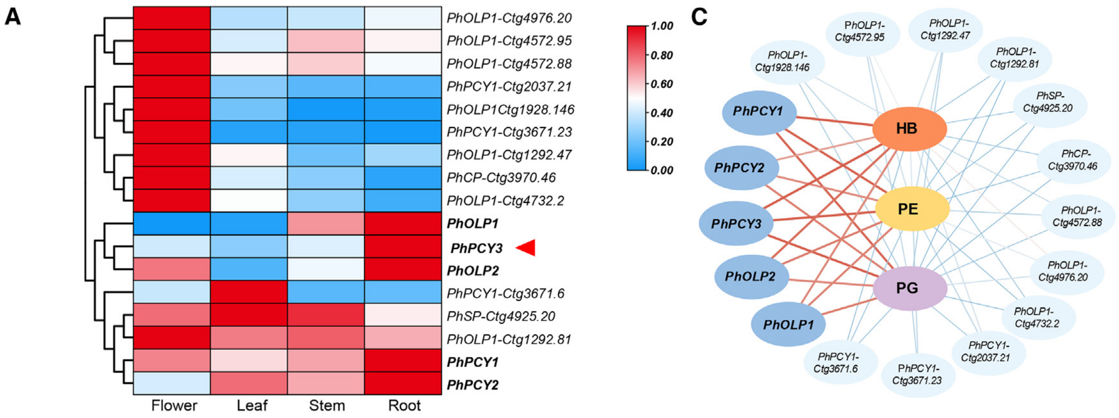

B

Candidate Genes	Pearson correlation coefficient		
	HB	PE	PG
PhPCY1 (PhPCY1-Ctg883.47)	0.992	0.957	0.930
PhPCY3 PhPCY1-Ctg883.50	0.985	0.987	0.983
PhOLP2 (PhOLP1-Ctg5689.43)	0.886	0.791	0.736
PhOLP1 (PhOLP1-Ctg4232.8)	0.714	0.752	0.791
PhPCY2 (PhPCY1-Ctg883.48)	0.568	0.702	0.765
PhOLP1-Ctg4976.20	0.062	-0.099	-0.197
PhOLP1-Ctg4572.95	-0.041	-0.214	-0.303
PhPCY1-Ctg3671.23	-0.131	-0.286	-0.380
PhPCY1-Ctg2037.21	-0.184	-0.331	-0.423
PhOLP1-Ctg1928.146	-0.192	-0.330	-0.421
PhOLP1-Ctg1292.47	-0.207	-0.322	-0.408
PhOLP1-Ctg4572.88	-0.227	-0.387	-0.476
PhCP-Ctg3970.46	-0.433	-0.559	-0.638
PhOLP1-Ctg4732.2	-0.452	-0.569	-0.646
PhOLP1-Ctg1292.81	-0.473	-0.617	-0.689
PhPCY1-Ctg3671.6	-0.583	-0.494	-0.474
PhSP-Ctg4925.20	-0.985	-0.941	-0.905

Figure 2. Potential key tailoring genes identified in *P. heterophylla*.

(A) Tissue-specific expression of the candidate genes in sampled tissues (flower, leaf, stem, and root).

(B) Pearson correlation coefficient between cyclic peptide content and candidate gene expression.

(C) Visual network interaction diagram between orbitides and candidate genes. The brown line indicates a Pearson correlation coefficient greater than 0.50.

(Figure 3E). These results suggest that PhPCY3 is the primary enzyme for orbitide biosynthesis.

Verification of PhPCY3 activity via VIGS in *P. heterophylla*

To further investigate the function of *PhPCY3 in planta*, a VIGS platform was employed. *ChlH* encodes the H subunit of magnesium protoporphyrin chelatase, an enzyme that plays a crucial role in chlorophyll biosynthesis. Silencing *ChlH* results in a yellow leaf phenotype in both *N. benthamiana* and *Gossypium arboreum* (Hiriart et al., 2002; Fan et al., 2023), making this gene a suitable control for our VIGS experiments. We silenced the *PhChlH1* gene in *P. heterophylla* using *Agrobacterium*-mediated transformation, resulting in a yellow leaf phenotype for about 4 weeks (Figure 4A and 4B; Supplemental Table 2).

Subsequently, we interfered with *PhPCY3* expression in the leaves and collected tissue samples to determine the expression of the target genes using quantitative real-time PCR (qRT-PCR) (Supplemental Table 3) and orbitide content using LC-MS. Quantitative real-time PCR results showed that *PhPCY3* was significantly downregulated compared with the negative control group (Figure 4C). Moreover, the HB, PE, and PG contents in these tissues decreased significantly when *PhPCY3* was silenced (Figure 4D). *PhPreHB*, *PhPrePE*, and *PhPrePG* were downregulated in leaves when these genes were silenced (Supplemental Figure 10A) which corresponded to the significantly decreased HB, PE, and PG levels (Supplemental Figure 10B). These results indicate that *PhPCY3* is essential for orbitide cyclization *in planta* and that *PreCPs* encode the necessary substrates for orbitide biosynthesis.

Key catalytic sites affect cyclization activity

Molecular docking between PhPCY3 and its substrates (PreCPs [14-35/56]) was performed to elucidate the potential cyclization mechanism. The core peptides and the C-terminal follower peptides are known to be substrates catalyzed by peptide cyclase 1 (Chekan et al., 2017). Molecular docking between the substrate PreCPs [14-35/56] and PhPCY3 in 4 Å revealed that amino acid residues in the pocket of PhPCY3 could form hydrogen bonds with the substrate amino acids (Figure 5A; Supplemental Figures 13–15). These catalytic sites include N500, which was identified across different substrates, suggesting its importance for tailoring various cyclic peptides (Supplemental Figure 12).

Group name	Module 1 (PreCPs)	Module 2 (PhOLPs)	Module 3 (PhPCYs)
pEAQ-HT (negative control)	–	–	–
PhPreHB	+	–	–
PhPreHB + PhOLPs	+	+	–
PhPreHB + PhPCYs	+	–	+
PhPreHB + PhOLPs + PhPCYs	+	+	+

Table 1. Modules of candidate genes in different groups.
PreCP refers to PreHB. + (–) refers to with (without) the candidate genes in this module. PhOLPs contain *PhOLP1/2*; PhPCYs include *PhPCY1/2/3*.

Multiple sequence alignment and amino acid domain analysis of PhPCY3/2/1 and the functionally validated *SvPCY1* gene revealed that N500 and S502 are sites that differ between functional and non-functional PCY1s. Notably, N500 is the first amino acid residue in the prolyl oligopeptidase domain (Pfam PF00326) of PhPCY3 (Figure 5B; Supplemental Figure 11). These results suggest that N500 and S502 may be key catalytic sites that affect cyclization activities.

To assess the roles of these residues, mutations N500 A/S/T, S502 A/T, and the double mutation N500A with S502A were introduced into PhPCY3 using site-directed mutagenesis PCR (Figure 5B; Supplemental Table 4). The catalytic activity of the mutated enzyme and HB content were measured using the transient *N. benthamiana* expression platform through LC-MS (Figure 5C; Supplemental Figure 16). The HB content decreased significantly when the N500 site was mutated to N500A or N500 S/T compared to the wild type (Figure 5C). Similar results were obtained when the S502 site was mutated to A or T. Moreover, the HB content was significantly reduced in tobacco leaves when both sites were mutated to A (Figure 5C; Supplemental Figure 16).

Identification of novel orbitides through the transient expression platform

Numerous precursor peptide genes of orbitides were identified by mining transcriptome data. These genes not only contained known orbitides but were also abundant with potential novel orbitides (Figure 6A and 6B). Several precursor peptide genes and known orbitides, including *PhPreHB*, *PhPrePE*, *PhPrePF*, and *PhPrePG,* were used to identify key tailoring genes (Figure 3). Analysis of amino acid sequences showed that the leader peptides at the N-terminal and the C-terminal ends of the follower peptide were highly conserved, whereas the core peptides displayed diverse cyclic peptide structures (Figure 6B). Novel orbitides, namely cyclo-[GLPIGAPWG], cyclo-[LDGPPPYF], cyclo-[WGSSTPHT], and cyclo-[FGDVG PVI], were identified using the *PhPCY3* heterologous expression platform. These compounds were characterized through comparison with established standards and the source plant (Figure 6C, 6E, 6G, and 6I). Furthermore, the chemical structures and partial fragmentation patterns of the orbitides were utilized to elucidate the peptide sequences. The ion fragment information from secondary mass spectrometry of the novel orbitides was consistent with the standards (Figure 6D, 6F, 6H, and 6J). In summary, we adopted reverse genetics to screen precursor peptide genes using multiple omics datasets, and a transient expression platform was utilized to identify key tailoring genes and orbitides (Supplemental Figure 17).

DISCUSSION

Natural products have long been explored through bioactivity-guided approaches. However, in recent years, the explosion of multi-omics data has accelerated the gene-guided discovery of novel natural products (Scherlach and Hertweck, 2021; Chigumba et al., 2022; Kersten et al., 2022; Pei et al., 2023; Mydy et al., 2024). Notably, non-squalene triterpenes have been identified by mining fungal chimeric class I triterpene synthases using a yeast-based genome mining platform (Chen et al., 2021; Tao et al., 2022). Cyclic peptides, with their diverse chemical structures and biological activities, are widespread throughout the plant kingdom and are well-suited for discovery via gene-guided approaches. For example, Fisher et al. used *de novo* transcriptomics and tandem mass spectrometry to rediscover evolidine and six novel orbitides in *Melicope xanthoxyloides* (Fisher et al., 2020). Furthermore, Song et al. developed sophisticated mining strategies to identify specific sequences within the *Linum usitatissimum* genome and used these data to investigate the genetic diversity of orbitides (Song et al., 2022). These identified sequences encompass multiple core peptide regions (CPRs), which contrasts the single CPR observed in orbitides from *P. heterophylla* and *M. xanthoxyloides*. However, various precursor peptide genes of potential orbitides were identified in the *P. heterophylla* transcriptome in this study, indicating that CPs exist *in planta* that have not yet been isolated.

Orbitides are the second largest identified group of plant cyclic peptides that do not contain disulfide bonds or non-natural amino acids. Numerous precursor peptide sequences have also been identified in several genera within the Rutaceae family by mining RNA-sequencing and whole genome sequencing data (Fisher et al., 2020). These peptides feature diverse residues at the C-terminus of CPRs, such as Phe, Leu, Ser, or Lys, which are not recognized by asparaginyl endopeptidase (Asn or Asp) or prolyl oligopeptidase (Pro or Ala), suggesting that one or more novel tailoring enzymes have yet to be discovered in Rutaceae. CPs discovered in the tuberous roots of *P. heterophylla* were isolated and identified in the 1990s using traditional phytochemical methods (Tan and Zhou, 2006). However, pseudostellarin K, the only orbitide discovered in the fibrous roots of *P. heterophylla*, was identified more recently (Zhao et al., 2020). This indicates that traditional methods may encounter bottlenecks in isolating low abundance natural products.

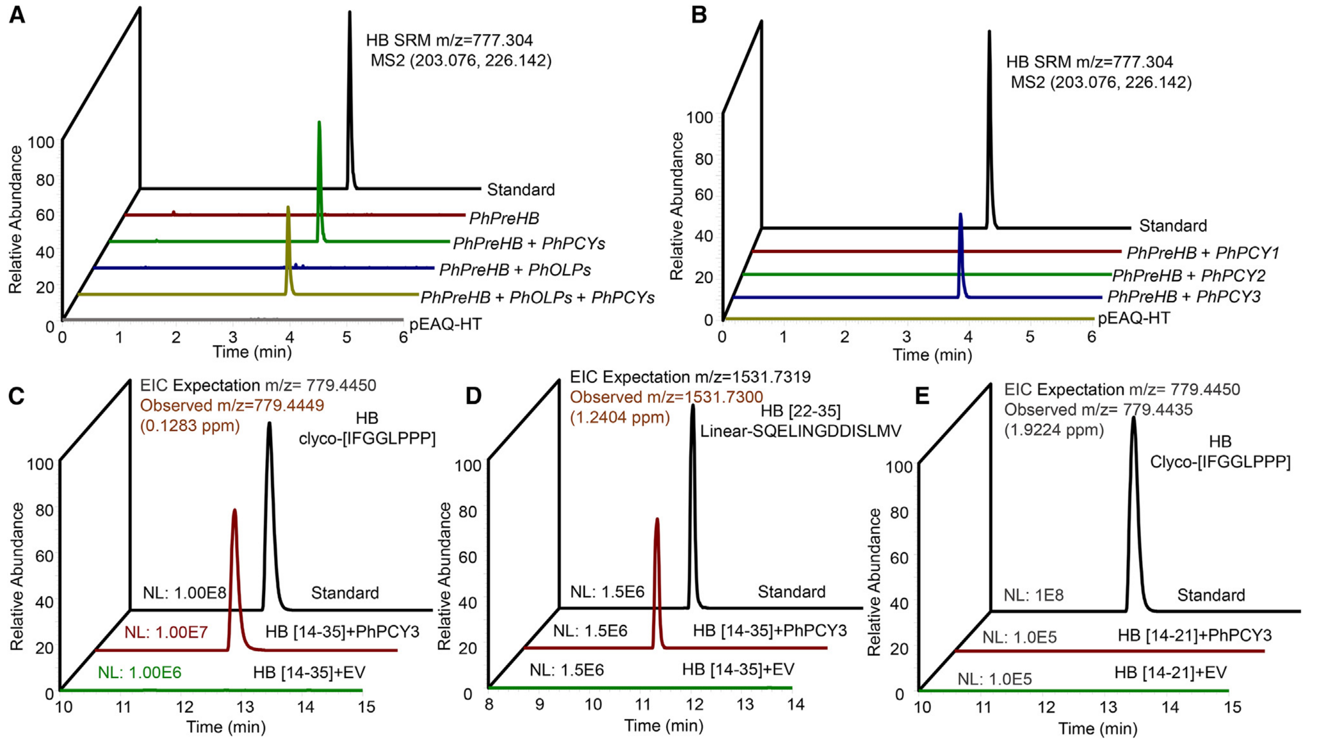

Figure 3. PhPCY3 dominates HB biosynthesis *in vivo* and *in vitro*.

(A) PhPCYs containing core genes in the heterophyllin B biosynthesis pathway. The *Nicotiana benthamiana* heterologous expression platform was used to verify the functions of candidate genes. All candidate genes were divided into three modules. Module 1 is the precursor protein gene *PhPreHB* necessary for the pathway. Module 2 includes two OLP genes encoding enzymes that may excise precursor peptides at the N-terminus. Module 3 contains three *PCY* genes encoding enzymes that may break intermediate products at the C-terminus and cyclize to form orbitides. *N. benthamiana* leaf samples were detected using LC-MS in the selective reaction monitoring (SRM) mode.

(B) Co-expressing *PhPreHB* and *PhPCY3* in *N. benthamiana* leaves can cyclize PreHB to produce HB.

(C and D) *In vitro* activity analysis of PhPCY3 with HB [14-35] as the substrate. PhPCY3 was expressed in the pMAL-c5x vector with an MBP tag and the purified protein was used to elucidate the catalytic mechanism *in vitro*. Extracted ion chromatograms of HB (dark red, observed m/z = 779.4449, z = 1, 0.1283 ppm) are shown with the standard (C). Extracted ion chromatograms of HB [22-35] (dark red, observed m/z = 1531.7300, z = 1, 1.2402 ppm) are shown with the standard. HB [22-35] refers to a linear peptide with the sequence SQELINGDDISLMV (D).

(E) *In vitro* activity of PhPCY3 with HB [14-21] as the substrate. Extracted ion chromatograms of HB (black, m/z = 779.4435, z = 1, 1.9224 ppm) are shown with the standard.

Multiple omics datasets have been used to explore candidate genes in the orbitide biosynthesis pathway. Many precursor genes were identified from our transcriptome data (Figure 6A). The amino acid sequences of the core peptide showed remarkable diversity compared with the flanking sequences on both sides, but the residues at the C-terminus of the core peptide were always Pro or Ala (Figure 6B), which can be recognized by SvPCY1 (Barber et al., 2013). A serine protease (SvOLP1) cleaves the linear peptide precursor presegetalin A1 [1-32] to presegetalin A1 [14–32], SvPCY1 then excises the intermediate at the C-terminal presegetalin A1 [20–32] and cyclizes the core peptide into segetalin A (Barber et al., 2013; Chekan et al., 2017). Genes encoding these enzymes in the *P. heterophylla* genome are hypothesized to be involved in orbitide biosynthesis pathways (Figure 2; Supplemental Figure 3). PhPCY3 has been demonstrated to participate in orbitide biosynthesis without the help of OLPs using a plant transient expression platform (Figure 3A and 3B). Meanwhile, PhPCY3 excises the HB [14-35] substrate at the C-terminus and cyclizes the propeptide into HB, as shown in an *in vitro* enzyme activity assay. Although the S9 family of proteases is found ubiquitously in nature, those with peptide ligase activity are much rarer (Nguyen et al., 2014). Furthermore, PhPCY3 is the second orbitide cyclase validated outside the original SvPCY1.

Previous studies have shown that some RiPP-modifying enzymes, such as those in thiazole/oxazole–modified microcin (TOMM) biosynthesis, recognize and bind the conserved N-terminal leader of precursor peptides (Burkhart et al., 2015; Koehnke et al., 2015). In addition, aspartate endopeptidases (AEPs) are cysteinyl enzymes that catalyze the hydrolysis and transpeptidation reaction and play a crucial role in the cleavage and cyclization of plant cyclic peptides. Karen et al. identified *OaAEP1*$_b$ from *Oldenlandia affinis* and found that its encoded enzyme couples the C-terminus cleavage of propeptide substrates with backbone cyclization, however, it cannot cleave the N-terminal site of cyclotide precursors (Harris et al., 2015). Similarly, CeAEP1 contains cleavage-coupled macrocyclization activity at the C-terminus to form SFTI-1 via intramolecular transpeptidation (Bernath-Levin et al., 2015). Interestingly, MCoAEP2 mediates both the N-terminal excision and the C-terminal cyclization of cyclotide

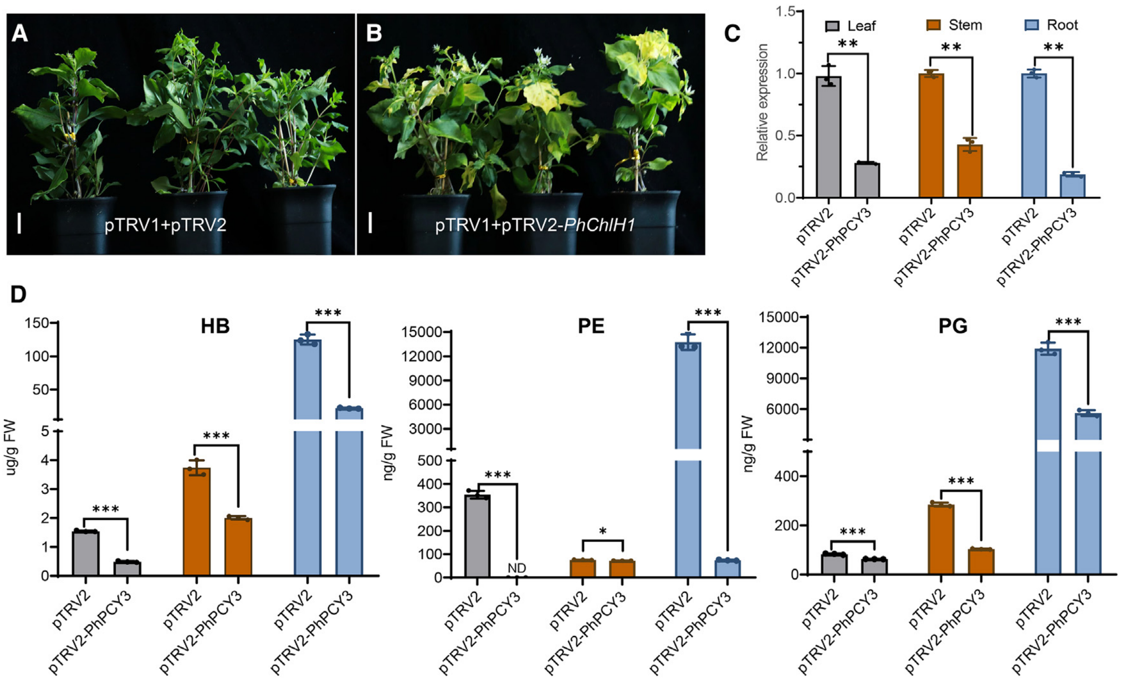

Figure 4. Silencing of the *PhPCY3* gene using the VIGS system.

(A) The negative control group was infiltrated with mixed strains containing pTRV1 and pTRV2 empty plasmids separately. The scale bar represents 2 cm.

(B) The positive control group was infiltrated with a mixture of strains containing pTRV1 and pTRV2-*PhChlH1* plasmids, showing a yellow leaf phenotype after 4 weeks of treatment. The scale bar represents 2 cm.

(C) Relative expression of the *PhPCY3* gene in different tissues in the pTRV2-PhPCY3 group compared to the negative control group.

(D) HB, PE, and PG contents in different tissues in the pTRV2-PhPCY3 group and the negative control group. The least significant difference method was used for statistical analysis after one-way ANOVA (completely randomized design) in DPS software ($n = 3$). *$p < 0.05$, **$p < 0.01$, ***$p < 0.001$, ND = not detected.

precursors (Du et al., 2020). However, SvPCY1, which belongs to the S9 class of the protease family, generates macrocyclic products by catalyzing the hydrolytic cleavage of peptide substrates at the C-terminus (Luo et al., 2014; Chekan et al., 2017). In the present study, hydrogen-bonding interactions were predicted between PhPCY3 residues and the follower peptide of PreCPs [14-35/36] at the C-terminus in 4 Å (Supplemental Figure 12).

The C-terminus follower peptides bind to SvPCY1 with hydrogen bond interactions via the triad amino acids (Ser562, Asp653, and His695), indicating that the triad plays a critical role in the cyclase during segetalin A biosynthesis (Chekan et al., 2017). The molecular basis of recognition and catalysis for SvPCY1 has been revealed, expanding the substrate scope of this enzyme (Ludewig et al., 2018). In the present study, multiple sequence alignment showed that the triad amino acids were conserved in PhPCY1/2/3 and SvPCY1, although PhPCY1/2 had no catalytic activity to produce orbitides. Amino acid sequence comparison between PhPCY3 and PhPCY1/2 and the molecular docking of PhPCY3 showed that N500 and S502 in the prolyl oligopeptidase domain were important for the catalytic activities of PhPCY3 via hydrogen bond interactions. However, our activity assays demonstrated that mutations at these two sites reduced but did not completely abolish catalytic activity. The phenotypic effects of the Ser502 mutation in PhPCY3 were consistent with those observed for the analogous Ser493 mutation in SvPCY1 (Ludewig et al., 2018). The cyclization mechanisms and processes vary among plants. Orbitides in the Caryophyllaceae and Rutaceae families likely involve distinct protease classes, underscoring the need for diverse approaches to uncover novel cyclization mechanisms. While the *PhPCY3* gene has proven effective in discovering orbitides in *P. heterophylla*, further evidence is still needed to confirm whether it can facilitate mining orbitides in other species.

RiPP CPs are a rich source of chemical and structural diversity, and macrocyclic peptides have emerged as excellent targets for drug discovery over the past decade (Passioura et al., 2014; Hosseinzadeh et al., 2017). Disulfide-rich CPs, in particular, have garnered increased attention in drug development, thanks to a yeast-based protocol that enables their biosynthesis via AEP *in vitro* (Yap et al., 2021). Here, we established an approach using the plant transient expression platform to discover and synthesize novel orbitides by mining transcriptome data (Supplemental Figure 17). These novel CPs could be screened for their anti-nematode activity using an open-source platform to analyze and share worm behavior (Javer et al., 2018a, 2018b; Barlow et al., 2022). This work presents a novel strategy for mining orbitides in plants with diverse cyclic peptide origins and structures and offers a feasible platform for elucidating the cyclization mechanisms of cyclic peptides in plants.

METHODS

Chemicals and reagents

All chemicals and reagents were purchased from commercial vendors. The authentic standard of HB (CAS:145 459-19-4) was obtained

A

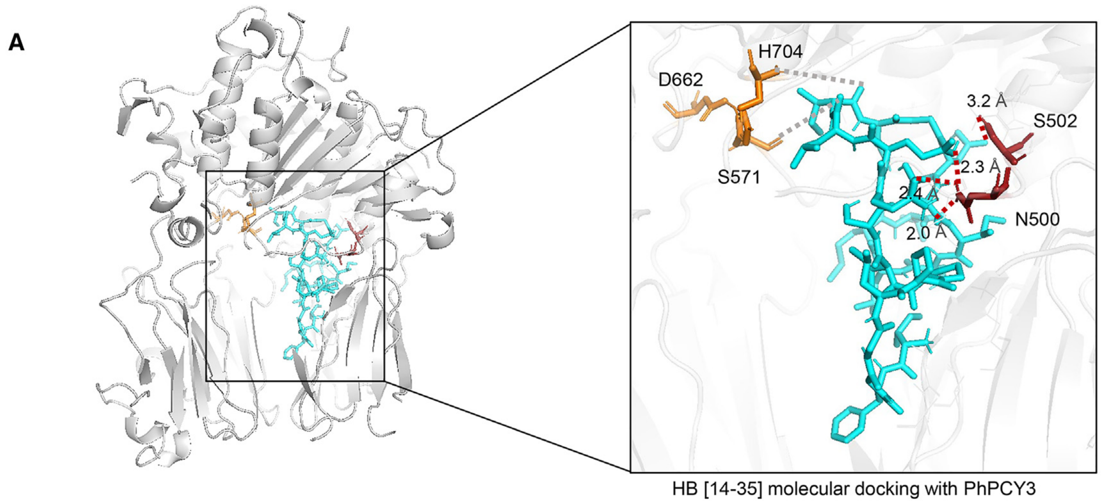

B

	Prolyl Oligopeptidase Domain (500-512)													Traid (S/D/H)		
Sites	500	501	502	503	504	505	506	507	508	509	510	511	512	571	662	704
SvPCY1	T	F	S	A	S	R	I	V	F	L	K	H	L	S (562)	D (653)	H (695)
PhPCY1	S	F	A	A	S	R	I	V	W	L	K	H	L	S	D	H
PhPCY2	S	F	A	A	S	R	I	V	W	L	K	H	L	S	D	H
PhPCY3	N	F	S	A	S	R	I	V	W	L	K	H	L	S (571)	D (662)	H(704)
	*		*													

C

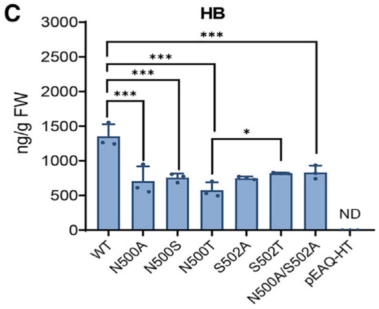

Figure 5. Key catalytic sites identified via molecular docking and multiple sequence alignment.

(A) Molecular docking of substrate HB [14-35] (cyan) with PhPCY3, showing hydrogen bonding interactions within 4 Å at the N500 and S502 sites, highlighted by dark red dashed lines. The catalytic triad amino acids (S571, D662 and H704) are shown in the molecular docking, with the residues nearest to the oxygen atom in the substrate highlighted by gray dashed lines.

(B) Key catalytic sites (N500 and S502) identified in the prolyl oligopeptidase domain (PF00326) of PCYs. The catalytic triad of amino acids in SvPCY1 and PhPCY3, including S571, D662, and H704, is also shown within the prolyl oligopeptidase domain.

(C) Quantitative analysis of HB content in tobacco leaves infiltrated with site-directed mutated PhPCY3 at the N500 and S502 sites by LC-MS in selected reaction monitoring (SRM) mode. The least significant difference method was used for statistical analysis after one-way ANOVA (completely randomized design) in DPS software ($n = 3$). $^{*}p < 0.05$ and $^{***}p < 0.001$.

commercially from Yuanye (Shanghai, China). Other authentic standards, including PE, PF, PG, and four novel orbitides, cyclo-[GLPIGAPWG], cyclo-[LDGPPPYF], cyclo-[WGSSTPHT], and cyclo-[FGDVGPVI], were chemically synthesized by ZPC (Zhejiang, China) and verified by LC-MS.

Collection of plant materials

Pseudostellaria heterophylla ("Kangbing No.1" cultivar, Linyi, Shandong province, China) seeds were soaked overnight in water. The moisture on the seed surface was maintained for one week, and the seeds were cultured in an incubator at 15°C for germination. The germinated seeds were sown in soil and grown in a phytotron at 25°C for 2 months. These seedlings are used for subsequent VIGS experiments. Flowers, leaves, stems, and roots of the "Zheseng No.2" cultivar of *P. heterophylla* (Zherong, Fujian province, China) were collected and quickly frozen in liquid nitrogen for orbitide content analysis and RNA-sequencing. Tuberous roots with buds from different cultivars were collected and planted in field plots at the Agricultural Genomics Institute at Shenzhen (Shenzhen, Guangdong, China) during the winter and collected the following summer. Tuber roots were used to mine precursor peptide genes and determine the orbitide content.

Identification of PreCPs, PCYs, and OLPs in *P. heterophylla*

Previously reported precursor peptide genes (PreCPs) of *S. vaccaria* and *P. heterophylla* were used as query sequences, and the PreCP sequences were extracted by BLASTP comparison with the transcript library. Considering that the coding sequence (CDS) of PreCP was extremely short (~108 base pairs [bp]), novel PreCP sequences were screened from the transcript libraries, which were assembled with transcriptome data from different cultivars using Trinity software (version 2.5.1) (Grabherr et al., 2011). PreCP sequences are listed in Supplemental Note 1. Given that PCY1 and OLP1 may be involved in the excision and cyclization of precursor peptide sequences (Craik and Malik, 2013), sequences of a serine protease, a cysteine protease, nine *OLP1* genes, and six *PCY1* genes that belong to the protease family in the *P. heterophylla* genome data are listed in Supplemental Note 2.

RNA-sequencing and correlation analysis

Total RNA was extracted from tissues of different *P. heterophylla* cultivars, as described above, using the FastPure® Plant Total RNA Isolation Kit (Vazyme). The Micropoly (A) Purist mRNA Purification Kit was used to purify mRNA from total RNA. Sequencing libraries were created and sequenced on the Illumina HiSeq 2500 platform to generate paired-end 125-bp reads. De novo and genome-guided transcriptome assembly was performed using Trinity (Version 2.5.1) with default parameters (Grabherr et al., 2011). Expression abundance was estimated with Topmast and Cufflinks using HTSeq (Version 0.6.1) (Trapnell et al., 2012). Pearson correlation coefficients between cyclic peptide contents and candidate gene expression were calculated using

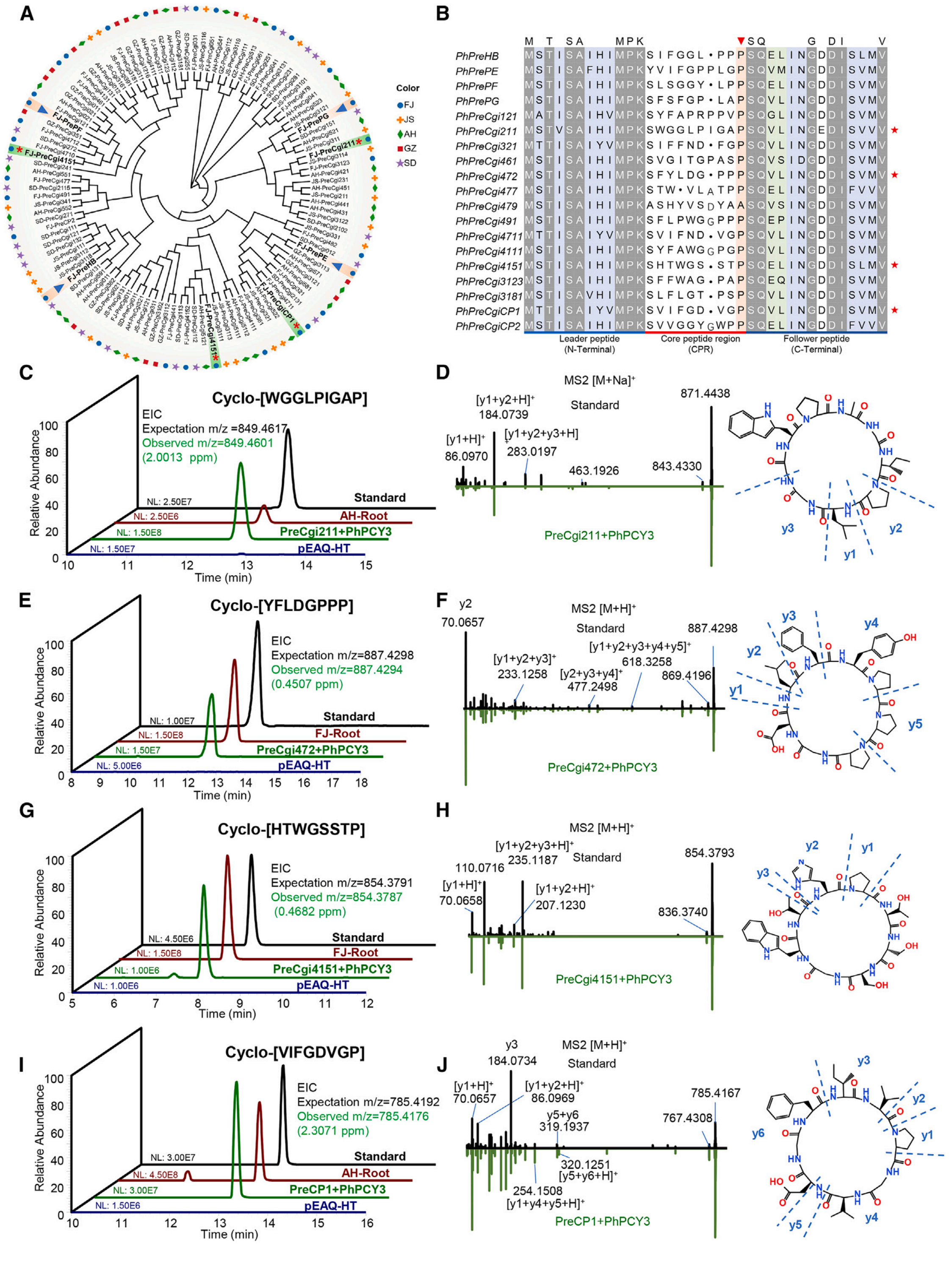

Figure 6. *PreCP*-guided discovery of novel orbitides.

(A) Phylogenetic analysis of precursor peptide sequences *in Pseudostellaria heterophylla*. A maximum likelihood tree was constructed using Mega software. Bootstrap values are indicated at the nodes (based on 1000 replicates). FJ, GZ, SD, AH, and JS refer to different *P. heterophylla* cultivars from Fujian, Guizhou, Shandong, Anhui, and Jiangsu provinces, respectively. Blue triangles represent precursor peptide sequences of known orbitides. Red asterisks indicate sequences of selected precursor peptides for novel orbitide discovery.

(B) Sequence alignment and conserved structure analysis of precursor peptide sequences in *P. heterophylla* (FJ). Amino acid sequences of the leader peptide (N-terminus) and the follower peptide (C-terminus) are conserved, while the core peptide regions (CPRs) show remarkable diversity. The last

(legend continued on next page)

Microsoft Excel, and Cytoscape software (Version 3.9.1) was used to visualize the data.

Plasmid construction

The target fragment of the gene of interest was amplified using KOD One PCR Master Mix (Toyobo) and gel purified with a DNA purification kit (Gel and PCR Clean-up, NucleoSpin). The pEAQ-HT vector was linearized using *Age*I and *Xho*I restriction enzymes (NEB), and the pTRV vector was linearized using *Eco*RI and *Bam*HI (NEB). All target fragments were introduced into vectors using the ClonExpress II One Step Cloning Kit (Vazyme). The primers are listed in Supplemental Tables 1 and 2. Assembled plasmids were transformed into JM109 (pEAQ-HT vector) and DH5α (pTRV vector) chemically competent cells and cultured overnight on selective Luria–Bertani (LB) plates containing 50 μg mL^{-1} kanamycin. Subsequently, positive transformants were screened by PCR, used to inoculate 5 mL of liquid LB, and cultured at 37°C with shaking (200 rpm). Plasmid extraction was performed using a TIANprep Mini Plasmid Kit (TIANGEN®), and the insert sequences were confirmed using Sanger sequencing (Shangya Biotechnology Co., Ltd., China). The verified plasmids were stored at −20°C for subsequent chemical transformation into the *Agrobacterium tumefaciens* strain GV3101.

Agrobacterium-mediated transient expression in *Nicotiana benthamiana*

Plasmids containing the target fragment of the gene of interest were transformed into *Agrobacterium tumefaciens* GV3101 (Rif^R, AC1001L, WEIDI) using the freeze-thaw method and selected on LB agar plates containing 50 μg mL^{-1} kanamycin and 25 μg mL^{-1} rifampicin at 28°C. Positive transformants were confirmed by PCR, used to inoculate 5 mL of liquid LB, and cultured at 28°C for 2 days. Cell culture (100 μL) was plated on previously-described double antibiotic resistance plates and incubated at 28°C overnight. Subsequently, these bacteria were collected and resuspended in induction buffer (10 mM MES buffer, pH 5.6, 10 mM $MgCl_2$, and 100 μM AS). Strain mixtures for co-expressing several candidate genes were maintained at an OD_{600} infiltration constant of 1.0. Cell suspensions were incubated at 28°C for 2 h before infiltration into *N. benthamiana* leaves.

N. benthamiana was seeded onto cylindrical rock wool blocks (20 mm × 27 mm, Grodan) and covered with vermiculite immersed in Hoagland's nutrient solution (Coolaber). Thereafter, seedlings were thinned twice over the next 2–3 weeks to leave a single plant on each rockwool block. These cylindrical rock wool blocks were then transferred to square rock wool blocks (50 mm × 50 mm × 40 mm, Grodan) for further rapid growth under a 16/8-h light/dark cycle for another 2–3 weeks. Three leaves were chosen for infiltration into the previously mixed suspensions to minimize any batch effects among leaves in different positions on the plants. Qualitative experiments were repeated three times on different tobacco plants. Quantitative experiments were performed on more than three plants for each combination. Infiltrated *N. benthamiana plants* were grown without light for 24 h and then under light conditions for 5 days before samples were collected. All samples were flash frozen using liquid nitrogen.

Metabolite extraction

Different tissues and heterogeneously expressed *N. benthamiana* leaves were ground into powder at 60 Hz for 45 s with a Tissuelyser tissue grinder (Tissuelyser-48L, Shanghai Jingxin) under liquid nitrogen quick-freezing conditions. We weighed 100 mg (when extracting tobacco leaf samples, we need accurately weigh 500 mg powder) of *P. heterophylla* powder into a 2 mL (15 mL) centrifuge tube and added 1 mL (5 mL) of chromatographic-grade methanol. Subsequently, these samples were thoroughly mixed using a vortex mixer (Vortex 3, IKA) and extracted at 40 kHz for 30 min at room temperature using an ultrasonic cleaner (SB25-12DT, SCIENTZ). These samples were then centrifuged at 4000 rpm for 15 min, and the supernatant was transferred to a new centrifuge tube. Tobacco samples were concentrated to 1 mL using a vacuum freezing centrifugal concentrator (CV600, JM) at 4°C and 1300 rpm, and all samples were treated with OASIS PRIME HLB (Waters) to remove pigments and impurities. These samples were filtered with an organic phase filter membrane (0.22 μm) using a blunt-end syringe and stored in brown sample vials for later analysis.

Enzyme activity assay for PhPCY3 *in vitro*

Two potential substrates, HB [14-35] (linear IFGGLPPPSQELINGDDIS LMV) and HB [14-21] (linear IFGGLPPP), as well as a standard for HB [22-35] (linear SQELINGDDISLMV), were synthesized by Sangon Biotech (Shanghai) Co., Ltd., and used to determine the catalytic activity of

amino acid of the core peptide is always Pro or Ala, marked by red triangles. The red star indicates the selected precursor peptide sequence for novel orbitide discovery.

(C) Discovery of novel orbitide cyclo-[WGGLPIGAP] by co-expressing *PhPreCgi211* and *PhPCY3* in tobacco leaves. The EIC map of cyclo-[GLPIGAPWG] in the source plant (AH-Root) and tobacco leaves compared with the synthetic standard. The theoretical value (accurate molecular weight) for cyclo-[GLPIGAPWG] was 849.4618. The observed m/z values were: standard sample (black), 849.4598 (2.3544 ppm); source plant sample (dark red), 849.4611 (0.8240 ppm); and tobacco leaf sample (green), 849.4601 (2.0013 ppm).

(D) MS2 particle fragment information for novel cyclo-[WGGLPIGAP] biosynthesized in *Nicotiana benthamiana* leaves compared with the synthetic standard. The chemical structure and partial fragmentation patterns of cyclo-[GLPIGAPWG] are illustrated.

(E) Discovery of novel orbitide cyclo-[YFLDGPPP] by co-expressing *PhPreCgi472* and *PhPCY3* in tobacco leaves. EIC map of cyclo-[YFLDGPPP] in the source plant (FJ-Root) and tobacco leaves compared with the synthetic standard. The theoretical value (accurate molecular weight) for cyclo-[YFLDGPPP] was 887.4298. The observed m/z values were: standard sample (black), 887.4282 (1.8030 ppm); source plant sample (dark red), 887.4286 (1.3522 ppm); and tobacco leaf sample (green), 887.4294 (0.4057 ppm).

(F) MS2 particle fragment information for novel cyclo-[YFLDGPPP] biosynthesized in *N. benthamiana* leaves compared with the synthetic standard. The chemical structure and partial fragmentation patterns of cyclo-[YFLDGPPP] are illustrated.

(G) Discovery of novel orbitide cyclo-[HTWGSSTP] by co-expressing *PhPreCgi4151* and *PhPCY3* in tobacco leaves. EIC map of cyclo-[HTWGSSTP] in the source plant (FJ-Root) and tobacco leaves compared with the synthetic standard. The theoretical value (accurate molecular weight) for cyclo-[HTWGSSTP] was 854.3791. The observed m/z values were: standard sample (black), 854.3788 (0.3511 ppm); source plant sample (dark red), 854.3773 (2.1068 ppm); and tobacco leaf sample (green), 854.3787 (0.4682 ppm).

(H) MS2 particle fragment information for novel cyclo-[HTWGSSTP] biosynthesized in *N. benthamiana* leaves compared with the synthetic standard. The chemical structure and partial fragmentation patterns of cyclo-[HTWGSSTP] are illustrated.

(I) Discovery of novel orbitide cyclo-[VIFGDVGP] by co-expressing *PhPreCP1* and *PhPCY3* in tobacco leaves. EIC map of cyclo-[VIFGDVGP] in the source plant (AH-Root) and tobacco leaves compared with the synthetic standard. The theoretical value (accurate molecular weight) for cyclo-[VIFGDVGP] was 785.4192. The observed m/z values were: standard sample (black), 785.4178 (1.7825 ppm); source plant sample (dark red), 785.4177 (1.9098 ppm); and tobacco leaf sample (green), 785.4176 (2.3071 ppm).

(J) MS2 particle fragment information for novel cyclo-[VIFGDVGP] biosynthesized in *N. benthamiana* leaves compared to the synthetic standard. The chemical structure and partial fragmentation patterns of cyclo-[VIFGDVGP] are illustrated.

PhPCY3 *in vitro*. We constructed the prokaryotic expression vector for this gene using pMAL-c5x with an MBP tag, while the negative control contained the empty plasmid (pMAL-c5x). Briefly, the pMAL-c5x vector was linearized through double digestion with *Bam*HI and *Eco*RI restriction enzymes (NEB). The primer sequences are listed in Supplemental Table 1. As previously described in the plasmid construction section, the vector was also assembled via homologous recombination. The resultant plasmid was introduced into the *Escherichia coli* strain BL21, and a positive clone was selected for induced expression and purification experiments. Expression and purification followed the protocols described in the NEBExpress MBP Fusion and Purification System manual (NEB #E8200S). The purified proteins were quantified using the BCA method and analyzed using SDS-PAGE. The induced expression and purification of proteins (250 ng/μL) were performed for subsequent enzyme activity experiments using 30 μM substrates, 20 mM Tris-HCl (pH 8.5), 5 mM dithiothreitol, and 100 mM NaCl in a 200 μL reaction system at 30°C for 1 h. Subsequently, an equal volume of methanol was used to extract the reaction products. After centrifugation (4°C, 5000 *g*, 5 min), the supernatant was analyzed using a Q Exactive HFX (LC-MS).

Quantitative and qualitative LC-MS analysis

The quantitative and qualitative analyses of the samples were conducted using two different LC-MS instrument setups: (1) Various *P. heterophylla* tissues and transiently expressing *N. benthamiana* leaves were evaluated using an UltiMate 3000 HPLC and Vanquish TSQ Quantum Access Max (Thermo) using electrospray ionization (ESI) in negative ionization mode with authentic standards. Quantitative analysis was performed by diluting the standard into different concentrations to precisely quantify the known orbitides. The detection method for HB, PE, PF, and PG was established in selective reaction monitoring (SRM) mode using an XBridge BEH C18 Column (Waters, 130 Å, 2.5 μm, 2.1 mm × 100 mm). The mobile phases consisted of acetonitrile (A) and 0.1% formic acid water (B), with a flow rate of 0.3 mL/min. The column temperature was maintained at 40°C, and the sample injection volume was fixed at 5 μL. Specific mobile phase elution gradients and mass spectrometry fragment ion information are provided in Supplemental Method 1. (2) Heterogeneously expressed novel orbitides in *N. benthamiana* leaves and the *in vitro* enzymatic products were detected using an UltiMate 3000 HPLC and Q Exactive HFX Mass Spectrometer (Thermo Scientific) in the full scan/ddMS2 mode. These tobacco samples underwent qualitative analysis using an ACQUITY UPLC BEH C18 VanGuard Pre-column (Waters, 130 Å, 1.7 μm, 2.1 mm × 5 mm) to determine the precise molecular weights of newly identified CPs. The mobile phase and flow velocity were consistent with those previously described. The LC-MS methods are detailed in Supplemental Method 1. Raw data were analyzed using Thermo Xcalibur software (Version 4.1) with either the Qual Browser or the Quan Browser app.

In vivo activity verification via the VIGS platform

The VIGS Tool (https://vigs.solgenomics.net/) was used to design silenced target sequences. Target gene segments were assembled into the pRTV2 vector by homologous recombination, as described above. The positive plasmids were transformed into the *Agrobacterium strain* GV3101, and the screening, activation, culturing, and resuspension of strains followed the same procedures detailed in *Agrobacterium*-mediated transient expression. Cell suspensions were mixed with a strain containing the pTRV1 vector and inoculated into the leaves of 3-week-old plants. Each treatment was applied to three pots of plants. *PhChlH1* was used as a positive control and exhibited a leaf-yellowing phenotype. Four weeks after *Agrobacterium* infiltration, the newly grown leaves at the top of the plants showed a stable yellow leaf phenotype, and different tissues were then collected for subsequent metabolic detection and gene expression analysis.

Quantitative real-time PCR analyses

VIGS samples were ground into powder using a Tissuelyser tissue grinder and total RNA was extracted using a FastPure Plant Total RNA Isolation Kit (Vazyme). To synthesize the first-strand cDNA, 1 μg of mRNA was mixed with TransScript All-in-One First-Strand cDNA Synthesis SuperMix (AT341-01, TRAN) to generate the cDNA template. The PCR reaction system (15 μL) included 0.6 μL of cDNA template, 7.5 μL of 2× TransStart Green qPCR SuperMix, and 0.45 μL of 10 mM upstream and downstream primers. The qRT-PCR primers are listed in Supplemental Table 3. Quantitative real-time PCR was performed using the Bio-Rad CFX Opus 96 Real-Time System, and the relative expression level of each gene was calculated using the $2^{(-\Delta\Delta Ct)}$ method compared with an internal control gene (β-actin) (Livak and Schmittgen, 2001). Three biological replicates were analyzed to determine the average value.

Molecular docking and sequence alignment

The protein structures of PhPCYs were predicted using AlphaFold (https://www.alphafold.ebi.ac.uk/). The chemical structure of the target substance was drawn using ChemDraw 20.0 software and converted to a MOL2 format file using Chem3D software. AutoDock Tools (Version 1.5.7) was used to prepare protein models and substrates, namely PreHB [14-35], PrePE [14-36], PrePF [14-35], and PrePG [14-35]. The molecular docking of PreCPs [14-35/36] with PhPCY3 was performed using AutoDock Vina (Forli et al., 2016). Subsequently, the interactions between the substrate ligand and receptor protein in 4 Å were visualized using PyMOL software. The key catalytic sites were identified using multiple sequence alignment (Multailin 5.4.1) and protein structure analysis, combined with information on molecular docking residues. The residues regarded as key sites of PhCPY3 involved in orbitide cyclization were mutated to their counterparts in PhPCY1/2. The plasmid pEAQ-HT-PhPCY3 (WT) was used as the template, and the primers for site-directed mutagenesis are listed in Supplemental Table 4. Site-directed mutagenesis was carried out using PCR and assembled with a multi-fragment homologous recombination kit (ClonExpress Ultra One Step Cloning Kit, C115, Vazyme). The resultant strains harboring the *PhPCY3* mutants were co-expressed with *PhPreHB* and assayed in the *N. benthamiana* system, as described above.

DATA AVAILABILITY

The data that support the findings of this study are available in the supplemental information of this article. We have uploaded raw sequencing reads (RNA-sequencing) to the NCBI SRA database under accession number SUB14839290.

FUNDING

This work was supported by the National Key R&D Program of China (Grant No. 2020YFA0907900 and 2017YFE0121800), and the National Natural Science Foundation of China (Grant No. 32170264, Grant No. 81573530, and Grant No. 31401950).

ACKNOWLEDGMENTS

We thank Dr. Ran Du for his support with chemical analysis, and Yanchun Peng and Meifeng Su from the Metabolic Platform at the AGIS for their assistance with metabolism analysis. We also thank Zhexue Sun, Hailang Xu, Fancheng Zeng, Congrong Jiang, Wenwen Zhang, Kui Wang, and Mingze Ma for their help in collecting and grinding samples. There are no conflicts of interest among the participants.

AUTHOR CONTRIBUTIONS

X.Q. and W.Li designed the research; X.Q. performed the research; F.W. and D.X. provided the analytical tools; X.Q. and Q.Z. collected VIGS samples; S.L. and W.Lin provided genome and transcriptome data; X.Q. and W.Li wrote and revised the paper.

SUPPLEMENTAL INFORMATION

Supplemental information is available at *Plant Communications Online*.

Received: July 11, 2024
Revised: January 22, 2025
Accepted: March 9, 2025
Published: March 13, 2025

REFERENCES

Barber, C.J.S., Pujara, P.T., Reed, D.W., Chiwocha, S., Zhang, H., and Covello, P.S. (2013). The two-step biosynthesis of cyclic peptides from linear precursors in a member of the plant family Caryophyllaceae involves cyclization by a serine protease-like enzyme. J. Biol. Chem. **288**:12500–12510.

Barlow, I.L., Feriani, L., Minga, E., McDermott-Rouse, A., O'Brien, T.J., Liu, Z., Hofbauer, M., Stowers, J.R., Andersen, E.C., Ding, S.S., and Brown, A.E.X. (2022). Megapixel camera arrays enable high-resolution animal tracking in multiwell plates. Commun. Biol. **5**:253.

Bernath-Levin, K., Nelson, C., Elliott, A.G., Jayasena, A.S., Millar, A.H., Craik, D.J., and Mylne, J.S. (2015). Peptide macrocyclization by a bifunctional endoprotease. Chem. Biol. **22**:571–582.

Burkhart, B.J., Hudson, G.A., Dunbar, K.L., and Mitchell, D.A. (2015). A prevalent peptide-binding domain guides ribosomal natural product biosynthesis. Nat. Chem. Biol. **11**:564–570.

Caplin, M.E., Pavel, M., Ćwikła, J.B., Phan, A.T., Raderer, M., Sedláčková, E., Cadiot, G., Wolin, E.M., Capdevila, J., Wall, L., et al. (2014). Lanreotide in metastatic enteropancreatic neuroendocrine tumors. N. Engl. J. Med. **371**:224–233.

Chekan, J.R., Estrada, P., Covello, P.S., and Nair, S.K. (2017). Characterization of the macrocyclase involved in the biosynthesis of RiPP cyclic peptides in plants. Proc. Natl. Acad. Sci. USA **114**:6551–6556.

Chekan, J.R., Mydy, L.S., Pasquale, M.A., and Kersten, R.D. (2024). Plant peptides – redefining an area of ribosomally synthesized and post-translationally modified peptides. Nat. Prod. Rep. **41**:1020–1059.

Chen, R., Jia, Q., Mu, X., Hu, B., Sun, X., Deng, Z., Chen, F., Bian, G., and Liu, T. (2021). Systematic mining of fungal chimeric terpene synthases using an efficient precursor-providing yeast chassis. Proc. Natl. Acad. Sci. USA **118**:e2023247118.

Chigumba, D.N., Mydy, L.S., de Waal, F., Li, W., Shafiq, K., Wotring, J.W., Mohamed, O.G., Mladenovic, T., Tripathi, A., Sexton, J.Z., et al. (2022). Discovery and biosynthesis of cyclic plant peptides via autocatalytic cyclases. Nat. Chem. Biol. **18**:18–28.

Commission, C.P. (2020). Chinese Pharmacopoeia (China Medical Science Press).

Condie, J.A., Nowak, G., Reed, D.W., Balsevich, J.J., Reaney, M.J.T., Arnison, P.G., and Covello, P.S. (2011). The biosynthesis of Caryophyllaceae-like cyclic peptides in *Saponaria vaccaria L.* from DNA-encoded precursors. Plant J. **67**:682–690.

Craik, D.J., and Malik, U. (2013). Cyclotide biosynthesis. Curr. Opin. Chem. Biol. **17**:546–554.

Dahiya, R., Dahiya, S., Shrivastava, J., Fuloria, N.K., Gautam, H., Mourya, R., and Fuloria, S. (2021). Natural cyclic polypeptides as vital phytochemical constituents from seeds of selected medicinal plants. Arch. Pharm. **354**:e2000446.

Daly, N.L., and Wilson, D.T. (2021). Plant derived cyclic peptides. Biochem. Soc. Trans. **49**:1279–1285.

De La Peña, R., Hodgson, H., Liu, J.C.-T., Stephenson, M.J., Martin, A.C., Owen, C., Harkess, A., Leebens-Mack, J., Jimenez, L.E., Osbourn, A., and Sattely, E.S. (2023). Complex scaffold remodeling in plant triterpene biosynthesis. Science **379**:361–368.

de Veer, S.J., Kan, M.W., and Craik, D.J. (2019). Cyclotides: From Structure to Function. Chem. Rev. **119**:12375–12421.

Deng, J., Feng, X., Zhou, L., He, C., Li, H., Xia, J., Ge, Y., Zhao, Y., Song, C., Chen, L., and Yang, Z. (2022). Heterophyllin B, a cyclopeptide from *Pseudostellaria heterophylla*, improves memory via immunomodulation and neurite regeneration in i.c.v.Aβ-induced mice. Food Res. Int. **158**:111576.

Du, J., Yap, K., Chan, L.Y., Rehm, F.B.H., Looi, F.Y., Poth, A.G., Gilding, E.K., Kaas, Q., Durek, T., and Craik, D.J. (2020). A bifunctional asparaginyl endopeptidase efficiently catalyzes both cleavage and cyclization of cyclic trypsin inhibitors. Nat. Commun. **11**:1575.

Fan, L., Hou, Y., Zheng, L., Shi, H., Liu, Z., Wang, Y., Li, S., Liu, L., Guo, M., Yang, Z., and Liu, J. (2023). Characterization and fine mapping of a yellow leaf gene regulating chlorophyll biosynthesis and chloroplast development in cotton (*Gossypium arboreum*). Gene **885**:147712.

Fisher, M.F., Payne, C.D., Rosengren, K.J., and Mylne, J.S. (2019). An Orbitide from *Ratibida columnifera* Seed Containing 16 Amino Acid Residues. J. Nat. Prod. **82**:2152–2158.

Fisher, M.F., Payne, C.D., Chetty, T., Crayn, D., Berkowitz, O., Whelan, J., Rosengren, K.J., and Mylne, J.S. (2020). The genetic origin of evolidine, the first cyclopeptide discovered in plants, and related orbitides. J. Biol. Chem. **295**:14510–14521.

Forli, S., Huey, R., Pique, M.E., Sanner, M.F., Goodsell, D.S., and Olson, A.J. (2016). Computational protein–ligand docking and virtual drug screening with the AutoDock suite. Nat. Protoc. **11**:905–919.

Grabherr, M.G., Haas, B.J., Yassour, M., Levin, J.Z., Thompson, D.A., Amit, I., Adiconis, X., Fan, L., Raychowdhury, R., Zeng, Q., et al. (2011). Full-length transcriptome assembly from RNA-Seq data without a reference genome. Nat. Biotechnol. **29**:644–652.

Harris, K.S., Durek, T., Kaas, Q., Poth, A.G., Gilding, E.K., Conlan, B.F., Saska, I., Daly, N.L., van der Weerden, N.L., Craik, D.J., and Anderson, M.A. (2015). Efficient backbone cyclization of linear peptides by a recombinant asparaginyl endopeptidase. Nat. Commun. **6**:10199.

Hemu, X., El Sahili, A., Hu, S., Wong, K., Chen, Y., Wong, Y.H., Zhang, X., Serra, A., Goh, B.C., Darwis, D.A., et al. (2019). Structural determinants for peptide-bond formation by asparaginyl ligases. Proc. Natl. Acad. Sci. USA **116**:11737–11746.

Hiriart, J.-B., Lehto, K., Tyystjärvi, E., Junttila, T., and Aro, E.-M. (2002). Suppression of a key gene involved in chlorophyll biosynthesis by means of virus-inducing gene silencing. Plant Mol. Biol. **50**:213–224.

Hosseinzadeh, P., Bhardwaj, G., Mulligan, V.K., Shortridge, M.D., Craven, T.W., Pardo-Avila, F., Rettie, S.A., Kim, D.E., Silva, D.A., Ibrahim, Y.M., et al. (2017). Comprehensive computational design of ordered peptide macrocycles. Science **358**:1461–1466.

Hua, Y., Wang, S., Liu, Z., Liu, X., Zou, L., Gu, W., Hou, Y., Ma, Y., Luo, Y., and Liu, J. (2016). iTRAQ-based quantitative proteomic analysis of cultivated *Pseudostellaria heterophylla* and its wild-type. J. Proteonomics **139**:13–25.

James, A.M., Haywood, J., and Mylne, J.S. (2018). Macrocyclization by asparaginyl endopeptidases. New Phytol. **218**:923–928.

Javer, A., Ripoll-Sánchez, L., and Brown, A.E.X. (2018a). Powerful and interpretable behavioural features for quantitative phenotyping of Caenorhabditis elegans. Philos. Trans. R. Soc. Lond. B Biol. Sci. **373**:20170375.

Javer, A., Currie, M., Lee, C.W., Hokanson, J., Li, K., Martineau, C.N., Yemini, E., Grundy, L.J., Li, C., Ch'ng, Q., et al. (2018b). An open-source platform for analyzing and sharing worm-behavior data. Nat. Methods **15**:645–646.

Jiang, B., Gao, L., Wang, H., Sun, Y., Zhang, X., Ke, H., Liu, S., Ma, P., Liao, Q., Wang, Y., et al. (2024). Characterization and heterologous reconstitution of Taxus biosynthetic enzymes leading to baccatin III. Science **383**:622–629.

Kandy, S.K., Pasquale, M.A., and Chekan, J.R. (2025). Aromatic side-chain crosslinking in RiPP biosynthesis. Nat. Chem. Biol. **21**:168–181.

Kersten, R.D., and Weng, J.K. (2018). Gene-guided discovery and engineering of branched cyclic peptides in plants. Proc. Natl. Acad. Sci. USA **115**:E10961–E10969.

Kersten, R.D., Mydy, L.S., Fallon, T.R., de Waal, F., Shafiq, K., Wotring, J.W., Sexton, J.Z., and Weng, J.K. (2022). Gene-Guided Discovery and Ribosomal Biosynthesis of Moroidin Peptides. J. Am. Chem. Soc. **144**:7686–7692.

Koehnke, J., Mann, G., Bent, A.F., Ludewig, H., Shirran, S., Botting, C., Lebl, T., Houssen, W., Jaspars, M., and Naismith, J.H. (2015). Structural analysis of leader peptide binding enables leader-free cyanobactin processing. Nat. Chem. Biol. **11**:558–563.

Li, X., Craven, T.W., and Levine, P.M. (2022). Cyclic Peptide Screening Methods for Preclinical Drug Discovery. J. Med. Chem. **65**:11913–11926.

Liao, H.J., and Tzen, J.T.C. (2022a). Investigating Potential GLP-1 Receptor Agonists in Cyclopeptides from *Pseudostellaria heterophylla, Linum usitatissimum*, and *Drymaria diandra*, and Peptides Derived from Heterophyllin B for the Treatment of Type 2 Diabetes: An In Silico Study. Metabolites **12**:549.

Liao, H.J., and Tzen, J.T.C. (2022b). The Potential Role of Cyclopeptides from *Pseudostellaria heterophylla, Linum usitatissimum* and *Drymaria diandra*, and Peptides Derived from Heterophyllin B as Dipeptidyl Peptidase IV Inhibitors for the Treatment of Type 2 Diabetes: An In Silico Study. Metabolites **12**:387.

Livak, K.J., and Schmittgen, T.D. (2001). Analysis of Relative Gene Expression Data Using Real-Time Quantitative PCR and the $2^{-\Delta\Delta CT}$ Method. Methods **25**:402–408.

Ludewig, H., Czekster, C.M., Oueis, E., Munday, E.S., Arshad, M., Synowsky, S.A., Bent, A.F., and Naismith, J.H. (2018). Characterization of the Fast and Promiscuous Macrocyclase from Plant PCY1 Enables the Use of Simple Substrates. ACS Chem. Biol. **13**:801–811.

Luo, H., Hong, S.Y., Sgambelluri, R.M., Angelos, E., Li, X., and Walton, J.D. (2014). Peptide macrocyclization catalyzed by a prolyl oligopeptidase involved in α-amanitin biosynthesis. Chem. Biol. **21**:1610–1617.

Ma, J., Yang, S., Liu, H., Shu, L., Yao, Y., Chen, S., Xu, Y., and Li, Y. (2023). Analysis of chemical components of Jianwei Xiaoshi Tablets based on UPLC-Q-Orbitrap-MS technology. Nat. Prod. Res. **11**:1–8.

Montalbán-López, M., Scott, T.A., Ramesh, S., Rahman, I.R., van Heel, A.J., Viel, J.H., Bandarian, V., Dittmann, E., Genilloud, O., Goto, Y., et al. (2021). New developments in RiPP discovery, enzymology and engineering. Nat. Prod. Rep. **38**:130–239.

Morita, H., Kayashita, T., Kobata, H., Gonda, A., Takeya, K., and Itokawa, H. (1994). Pseudostellarins D - F, new tyrosinase inhibitory cyclic peptides from *Pseudostellaria heterophylla*. Tetrahedron **50**:9975–9982.

Muttenthaler, M., King, G.F., Adams, D.J., and Alewood, P.F. (2021). Trends in peptide drug discovery. Nat. Rev. Drug Discov. **20**:309–325.

Mydy, L.S., Hungerford, J., Chigumba, D.N., Konwerski, J.R., Jantzi, S.C., Wang, D., Smith, J.L., and Kersten, R.D. (2024). An intramolecular macrocyclase in plant ribosomal peptide biosynthesis. Nat. Chem. Biol. **20**:530–540.

Nguyen, G.K.T., Wang, S., Qiu, Y., Hemu, X., Lian, Y., and Tam, J.P. (2014). Butelase 1 is an Asx-specific ligase enabling peptide macrocyclization and synthesis. Nat. Chem. Biol. **10**:732–738.

Passioura, T., Katoh, T., Goto, Y., and Suga, H. (2014). Selection-based discovery of druglike macrocyclic peptides. Annu. Rev. Biochem. **83**:727–752.

Pei, Z.-F., Zhu, L., and Nair, S.K. (2023). Core-dependent post-translational modifications guide the biosynthesis of a new class of hypermodified peptides. Nat. Commun. **14**:7734.

Qin, X., Wu, H., Chen, J., Wu, L., Lin, S., Khan, M.U., Boorboori, M.R., and Lin, W. (2017). Transcriptome analysis of *Pseudostellaria heterophylla* in response to the infection of pathogenic *Fusarium oxysporum*. BMC Plant Biol. **17**:155.

Reed, J., Orme, A., El-Demerdash, A., Owen, C., Martin, L.B.B., Misra, R.C., Kikuchi, S., Rejzek, M., Martin, A.C., Harkess, A., et al. (2023). Elucidation of the pathway for biosynthesis of saponin adjuvants from the soapbark tree. Science **379**:1252–1264.

Ryan, D.H. (2020). Setmelanotide: what does it mean for clinical care of patients with obesity? Lancet Diabetes Endocrinol. **8**:933–935.

Scherlach, K., and Hertweck, C. (2021). Mining and unearthing hidden biosynthetic potential. Nat. Commun. **12**:3864.

Slazak, B., Haugmo, T., Badyra, B., and Göransson, U. (2020). The life cycle of cyclotides: biosynthesis and turnover in plant cells. Plant Cell Rep. **39**:1359–1367.

Song, Z., Burbridge, C., Schneider, D.J., Sharbel, T.F., and Reaney, M.J.T. (2022). The flax genome reveals orbitide diversity. BMC Genom. **23**:534.

Tan, N.H., and Zhou, J. (2006). Plant Cyclopeptides. Chem. Rev. **106**:840–895.

Tao, H., Lauterbach, L., Bian, G., Chen, R., Hou, A., Mori, T., Cheng, S., Hu, B., Lu, L., Mu, X., et al. (2022). Discovery of non-squalene triterpenes. Nature **606**:414–419.

Tehrani, M., Gholibeikian, M., Bamoniri, A., and Bi, B. (2021). Cancer Treatment by Caryophyllaceae-Type Cyclopeptides. Front. Endocrinol. **11**:600856.

Trapnell, C., Roberts, A., Goff, L., Pertea, G., Kim, D., Kelley, D.R., Pimentel, H., Salzberg, S.L., Rinn, J.L., and Pachter, L. (2012). Differential gene and transcript expression analysis of RNA-seq experiments with TopHat and Cufflinks. Nat. Protoc. **7**:562–578.

Wu, H., Qin, X., Wang, J., Wu, L., Chen, J., Fan, J., Zheng, L., Tangtai, H., Arafat, Y., Lin, W., et al. (2019). Rhizosphere responses to environmental conditions in *Radix pseudostellariae* under continuous monoculture regimes. Agric. Ecosyst. Environ. **270–271**:19–31.

Xiao, Q., Zhao, L., Jiang, C., Zhu, Y., Zhang, J., Hu, J., and Wang, G. (2022). Polysaccharides from *Pseudostellaria heterophylla* modulate gut microbiota and alleviate syndrome of spleen deficiency in rats. Sci. Rep. **12**:20217.

Xu, H., Li, W., Schilmiller, A.L., van Eekelen, H., de Vos, R.C.H., Jongsma, M.A., and Pichersky, E. (2019). Pyrethric acid of natural pyrethrin insecticide: complete pathway elucidation and reconstitution in *Nicotiana benthamiana*. New Phytol. **223**:751–765.

Yang, Q., Cai, X., Huang, M., Chen, X., Tian, Y., Chen, G., Wang, M., Wang, S., and Xiao, J. (2020). Isolation, Identification, and Immunomodulatory Effect of a Peptide from *Pseudostellaria heterophylla* Protein Hydrolysate. J. Agric. Food Chem. **68**:12259–12270.

Yang, Z., Zhang, C., Li, X., Ma, Z., Ge, Y., Qian, Z., and Song, C. (2021). Heterophyllin B, a cyclopeptide from *Pseudostellaria heterophylla*, enhances cognitive function via neurite outgrowth and synaptic plasticity. Phytother Res. **35**:5318–5329.

Yap, K., Du, J., Rehm, F.B.H., Tang, S.R., Zhou, Y., Xie, J., Wang, C.K., de Veer, S.J., Lua, L.H.L., Durek, T., and Craik, D.J. (2021). Yeast-based

bioproduction of disulfide-rich peptides and their cyclization via asparaginyl endopeptidases. Nat. Protoc. **16**:1740–1760.

Zhang, H., and Chen, S. (2022). Cyclic peptide drugs approved in the last two decades (2001–2021). RSC Chem. Biol. **3**:18–31.

Zhang, Y., Wiese, L., Fang, H., Alseekh, S., Perez de Souza, L., Scossa, F., Molloy, J., Christmann, M., and Fernie, A.R. (2023). Synthetic biology identifies the minimal gene set required for paclitaxel biosynthesis in a plant chassis. Mol. Plant **16**:1951–1961.

Zhao, X.-f., Zhang, Q., Zhao, H.-t., Zhang, Y.-d., Liu, H.-r., Yuan, L.-j., and Chen, Q.-f. (2022). A new cyclic peptide from the fibrous root of *Pseudostellaria heterophylla*. Nat. Prod. Res. **36**:3368–3374.

Zheng, W., Zhou, T., Li, J., Jiang, W., Zhang, J., Xiao, C., Wei, D., Yang, C., Xu, R., Gong, A., et al. (2019). The Biosynthesis of Heterophyllin B in *Pseudostellaria heterophylla* From prePhHB-Encoded Precursor. Front. Plant Sci. **10**:1259.

Zhou, Q., Sun, P., Xiong, H.M., Xie, J., Zhu, G.Y., Tantillo, D.J., and Huang, A.C. (2024). Insight into neofunctionalization of 2,3-oxidosqualene cyclases in B,C-ring-opened triterpene biosynthesis in quinoa. New Phytol. **241**:764–778.

Plant Communications, Volume *6*

Supplemental information

Identification of a key peptide cyclase for novel cyclic peptide discovery in *Pseudostellaria heterophylla*

Xianjin Qin, Fengjiao Wang, Dejin Xie, Qi Zhou, Sheng Lin, Wenxiong Lin, and Wei Li

Supplemental information

Identification of a key peptide cyclase for novel cyclic peptide discovery in *Pseudostellaria heterophylla*

Xianjin Qin, Fengjiao Wang, Dejin Xie, Qi Zhou, Sheng Lin, Wenxiong Lin and Wei Li

***Correspondence:** **Wenxiong Lin** (lwx@fafu.edu.cn), **Wei Li** (liwei11@caas.cn).

Supplemental information

Article title: **Identification of a key peptide cyclase for novel cyclic peptide discovery in *Pseudostellaria heterophylla***

Authors: Xianjin Qin, Fengjiao Wang, Dejin Xie, Qi Zhou, Sheng Lin, Wenxiong Lin and Wei Li

This PDF file includes:

Supplemental Figures 1 to 17
Supplemental Tables 1 to 4
Supplemental Method 1
Legends for Supplemental Note 1 to 2

Other supporting materials for this manuscript include the following:

Supplemental Note 1 to 2

Supplemental Figure 1. Chemical structure and chromatograms of CPs in *P. heterophylla.*

Supplemental Figure 2. Particle fragment information of HB and PE/PF/PG in secondary mass spectrometry.

Supplemental Figure 3. Potential biosynthesis pathway of orbitides in *P. heterophylla*.

Supplemental Figure 4. Phylogenetic analysis of the PhPCYs genes with other known cyclase gene *SvPCY1* and *HaAEP*.

Supplemental Figure 5. Two specific daughter ion fragment information of peaks in different groups in the selected Ion monitoring (SRM) mode.

Supplemental Figure 6. *PhOLP1* and *PhOLP2* were not involved in the cyclization of Heterophyllin B.

Supplemental Figure 7. *PhPCY3* cyclizes different *PhPreCPs* into orbitides in the transient expression platform.

Supplemental Figure 8. The SDS-PAGE image shows the purification of pMAL-MBP-tag and PhPCY3 proteins for the *in vitro* assay.

Supplemental Figure 9. MS2 particle fragment information of HB and HB [22-35] in the enzyme activity assay of Ph PCY3 with HB [14-35] *in vitro*.

Supplemental Figure 10. VIGS the *PhPreCP* genes involved in orbitides biosynthesis *in planta*.

Supplemental Figure 11. Amino acid sequence alignment and mutation site analysis of *PhPCY1/2/3* and *SvPCY1*.

Supplemental Figure 12. Key catalytic sites identified via molecular docking.

Supplemental Figure 13. Molecular docking of the substrate PE [14-36] with PhPCY3 within 4 Å and triad amino acids.

Supplemental Figure 14. Molecular docking of the substrate PF [14-35] with PhPCY3 within 4 Å and triad amino acids.

Supplemental Figure 15. Molecular docking of the substrate PG [14-35] with PhPCY3 within 4 Å and triad amino acids.

Supplemental Figure 16. The ion chromatograms (SRM) of heterologous

expression mutations of PhPCY3 with PhPreHB in *N. benthamiana* leaves.

Supplemental Figure 17. Reverse genetics strategy for mining novel orbitides via a transient expression platform.

Supplemental Table 1. Primers for all the candidate genes with pEAQ-HT and pMal-c5x vectors.

Supplemental Table 2. Primers for the candidate genes with pTRV2 vector.

Supplemental Table 3. qRT-PCR primers for precursor peptide genes and PhPCY3.

Supplemental Table 4. Primers for site-directed mutation at N500 and S502 site of PhPCY3.

Supplemental Method 1. Supplementary methods for LC-MS analysis.

Supplemental Note 1. The CDS sequence of precursor peptide genes in *P. heterophylla*.

Supplemental Note 2. Sequences of the candidate genes that may be involved in orbitide biosynthesis.

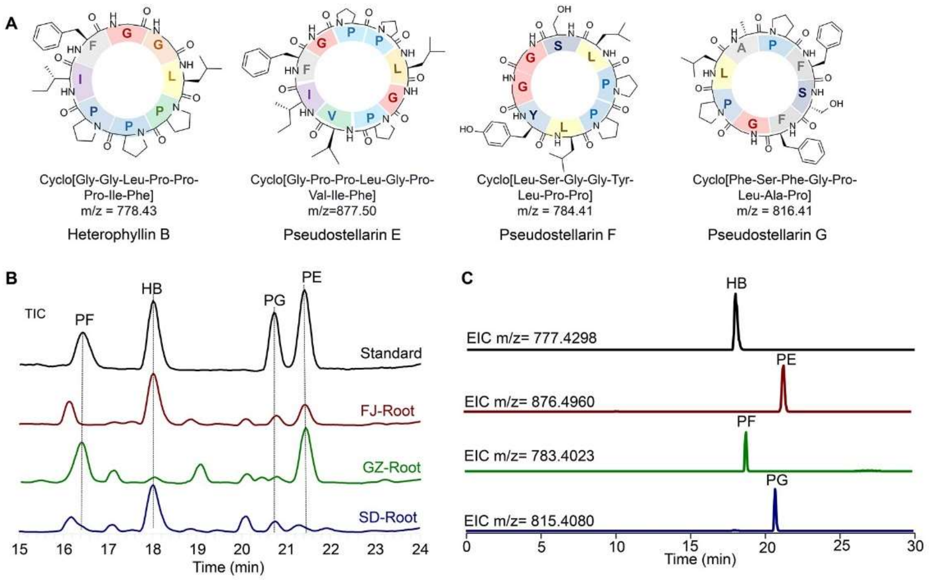

Supplemental Figure 1. Chemical structure and chromatograms of orbitides in *P. heterophylla.*

(A) Chemical structure of heterophyllin B and pseudostellarin E/F/G.

(B) The total ion chromatograms (TICs) of the root extracts of the cultivars (FJ-ZS II: Main cultivar cultivated in Fujian province, GZ-ST: Main cultivar cultivated in Guizhou province, SD-KB: Main cultivar cultivated in Guizhou province) with the known orbitides (including heterophyllin B and pseudostellarin E/F/G) using Q Exactive^{TM} HFX LC-MS/MS.

(C) Extracted ion chromatograms (EICs) of HB, PE, PF, and PG.

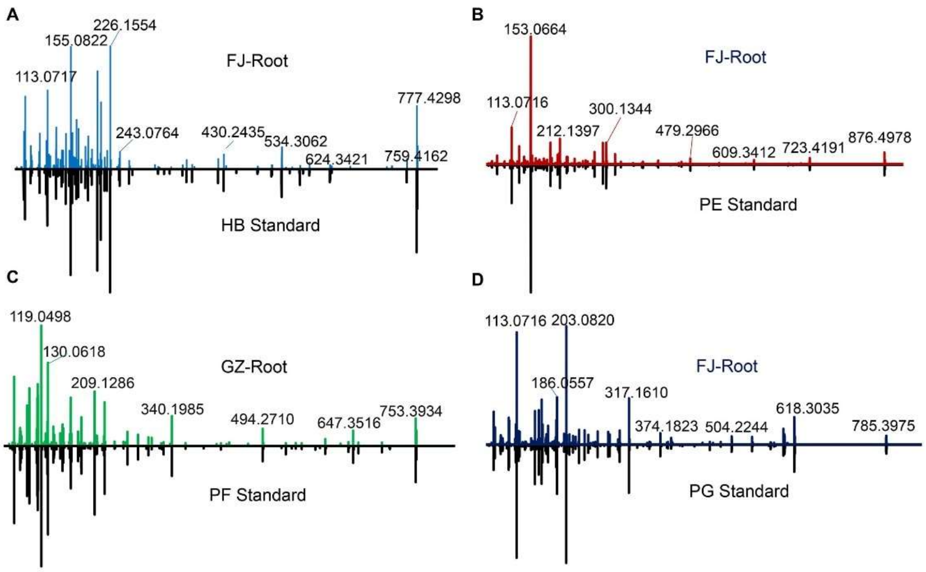

Supplemental Figure 2. Particle fragment information of HB and PE/PF/PG in secondary mass spectrometry.

(A) Particle fragment information of HB in secondary mass spectrometry (MS2).

(B) Particle fragment information of PE in secondary mass spectrometry (MS2).

(C) Particle fragment information of PF in secondary mass spectrometry (MS2).

(D) Particle fragment information of PG in secondary mass spectrometry (MS2).

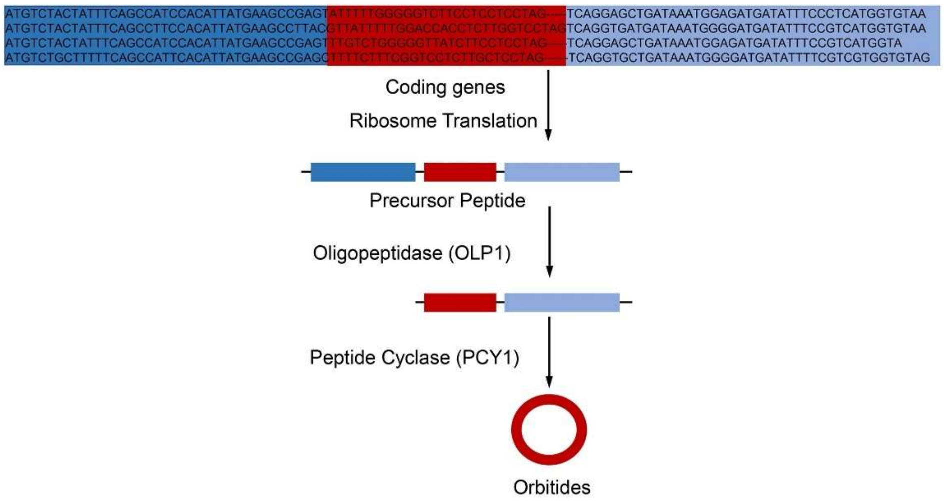

Supplemental Figure 3. Potential biosynthesis pathway of orbitides in *P. heterophylla*.

The precursor peptide genes were translated into linear precursor peptides. Oligopeptidase (OLP1) and peptide cyclase (PCY1) may be involved in excise at the N-terminal and the cyclize at the C-terminal.

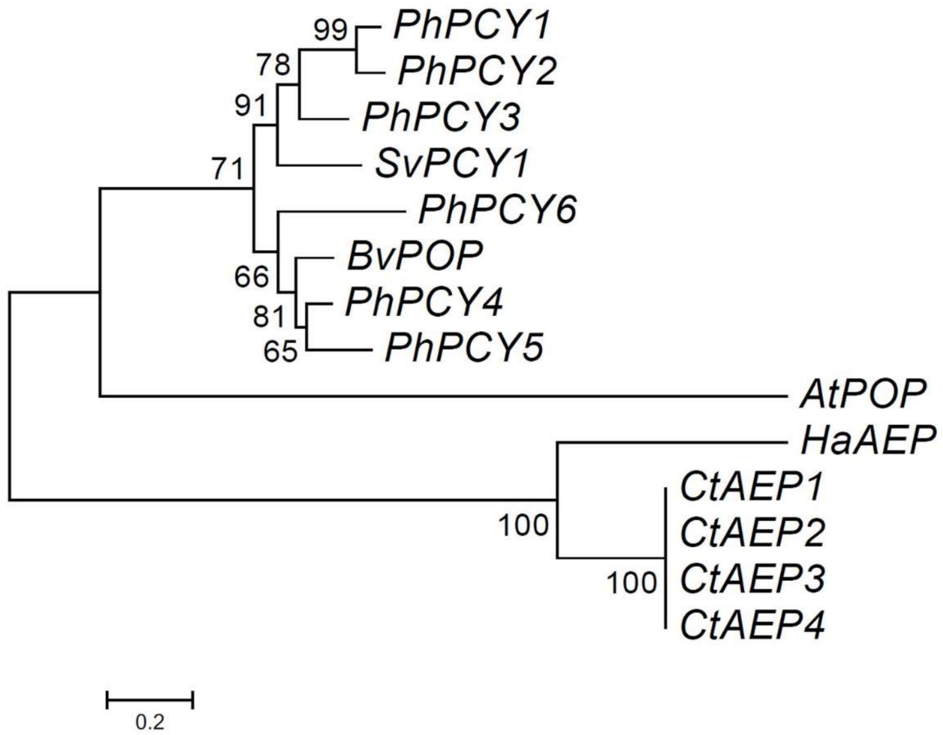

Supplemental Figure 4. Phylogenetic analysis of the PhPCYs genes with other known cyclase gene *SvPCY1* and *HaAEP*.

The maximum likelihood tree was constructed by Mega software. Bootstrap values are indicated in the nodes (based on 1000 replicates). *SvPCY1*: *PCY1* gene from *Saponaria vaccaria*, *BvPOP* and *AtPOP* refers to the prolyl oligopeptidase gene from *Beta vulgaris* and *Arabidopsis thaliana,* respectively. *HaAEP* and *CtAEP1/2/3/4* refer to asparaginyl endopeptidase gene from *Helianthus annuus* and *Clitoria ternatea*. PhPCYs refer to *PhPCY1*: *PhPCY1-Ctg883.47*, *PhPCY2*: *PhPCY1-Ctg883.48*, *PhPCY3*: *PhPCY1-Ctg883.50, PhPCY4*: *PhPCY1-Ctg2037.21*, *PhPCY5*: *PhPCY1-Ctg3671.23*, *PhPCY6*: *PhPCY1-Ctg3671.6*, respectively.

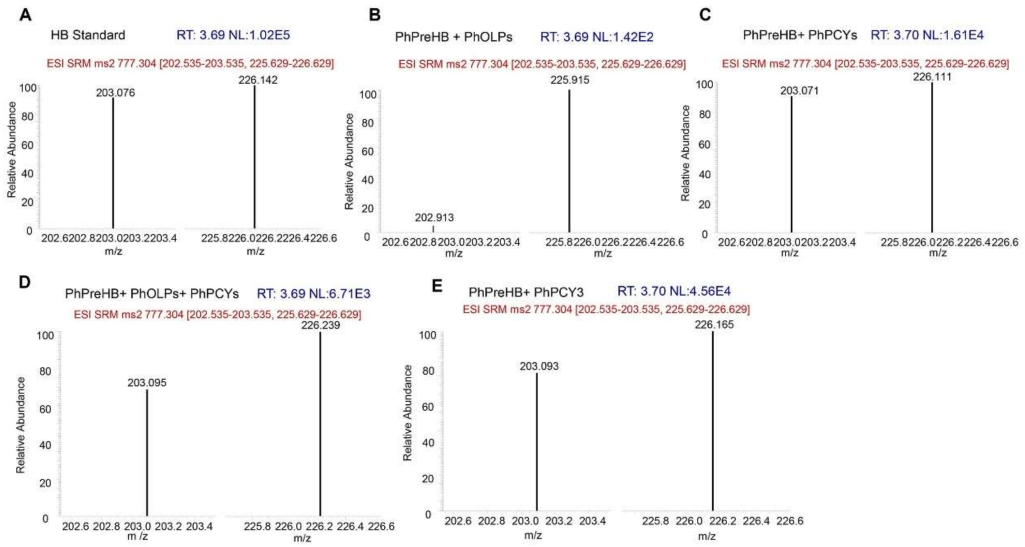

Supplemental Figure 5. Two specific daughter ion fragment information of peaks in different groups in the selected Ion monitoring (SRM) mode.

(A) Two specific daughter ion fragment information of the HB peak in SRM mode (the peak was HB standard).

(B) Two specific daughter ion fragment information of the PhPreHB + PhOLPs group peak in SRM mode (the tiny peak was not HB).

(C) Two specific daughter ion fragment information of the PhPreHB + PhPCYs group peak in SRM mode.

(D) Two specific daughter ion fragment information of the PhPreHB +PhOLPs + PhPCYs group peak in SRM mode.

(E) Two specific daughter ion fragment information of the PhPreHB +PhPCY3 group peak in SRM mode.

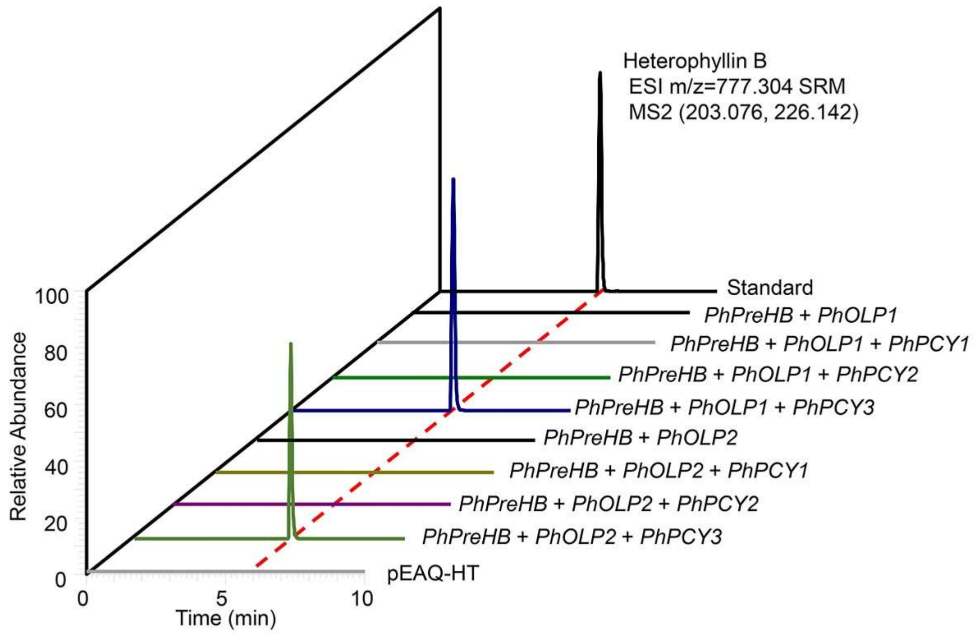

Supplemental Figure 6. *PhOLP1* and *PhOLP2* were not involved in the cyclization of Heterophyllin B.

The ion chromatograms of *N. benthamiana* leaf samples which combine *PhPreHB* with *PhOLP1/2* and *PhPCY1/2/3* were detected using LC-MS in the selected reaction monitoring (SRM) mode. *PhOLP1* refers to *PhOLP1-Ctg4232.8* and *PhOLP2* refers to *PhOLP1-Ctg5689.43.*

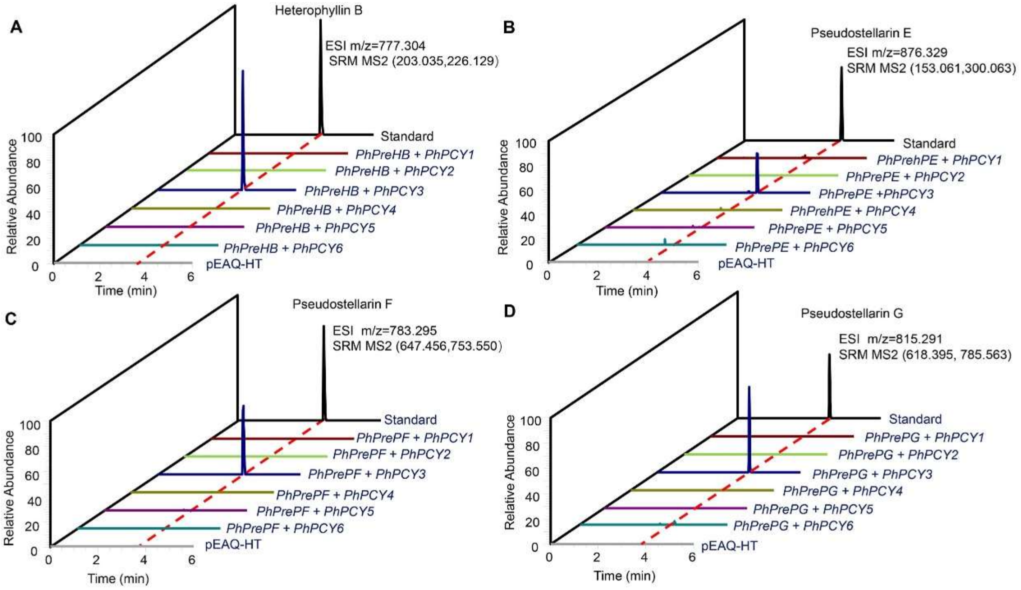

Supplemental Figure 7. PhPCY3 cyclizes *PhPreCPs* into orbitides in transient expression platform.

(A) *PhPCY3* can cyclize *PhPreHB* to heterophyllin B.

(B) *PhPCY3* cyclizes *PhPrePE* to pseudostellarin E.

(C) *PhPCY3* can cyclize *PhPrePF* to pseudostellarin F.

(D) *PhPCY3* can cyclize *PhPrePG* to pseudostellarin G.

The ion chromatograms of *N.benthamiana* leaf samples which combine *PhPreHB*, *PhPrePE*, *PhPrePF,* and *PhPrePG* with *PhPCY1/2/3/4/5/6* were detected using LC-MS in the selected reaction monitoring (SRM) mode, respectively.

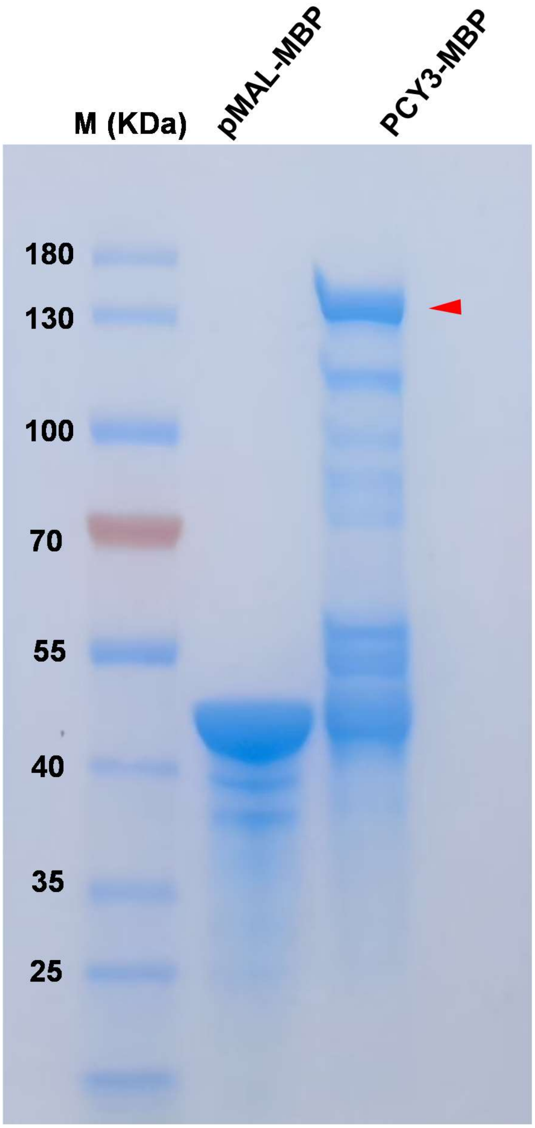

Supplemental Figure 8. The SDS-PAGE image shows the purification of pMAL-MBP-tag and PhPCY3 proteins for the *in vitro* assay. The MBP tag ia about 42.5 KDa, and PhPCY3 is about 80.7 KDa. Thus, the size of the fusion expression protein with MBP tag after induction is about 123.3 KDa (The red arrows indicate fusion expressed proteins).

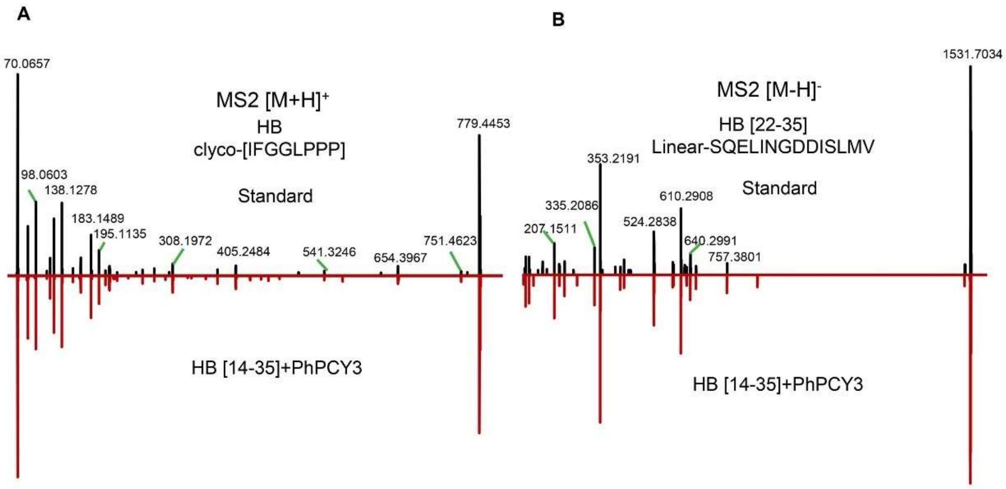

Supplemental Figure 9. MS2 particle fragment information of HB and HB [22-35] in the enzyme activity assay of PhPCY3 with HB [14-35] *in vitro*.

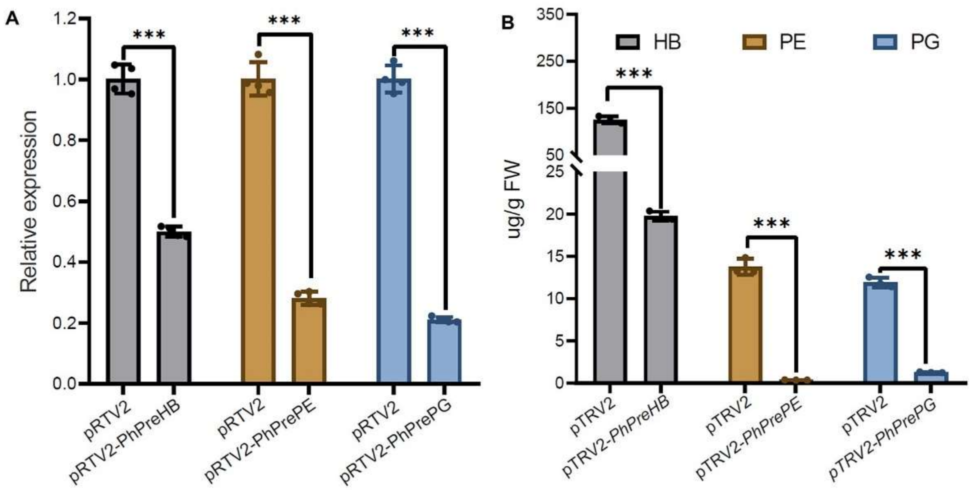

Supplemental Figure 10. VIGS the *PhPreCP* genes involved in orbitides biosynthesis *in planta*.

(A) The relative expression of the *PhPreHB*, *PhPrePE,* and *PhPrePG* gene *in P. heterophylla* leaves when these genes were silenced *in planta*.

(B) The HB, PE, and PG contents *in P. heterophylla* roots when these genes were silenced *in planta*.

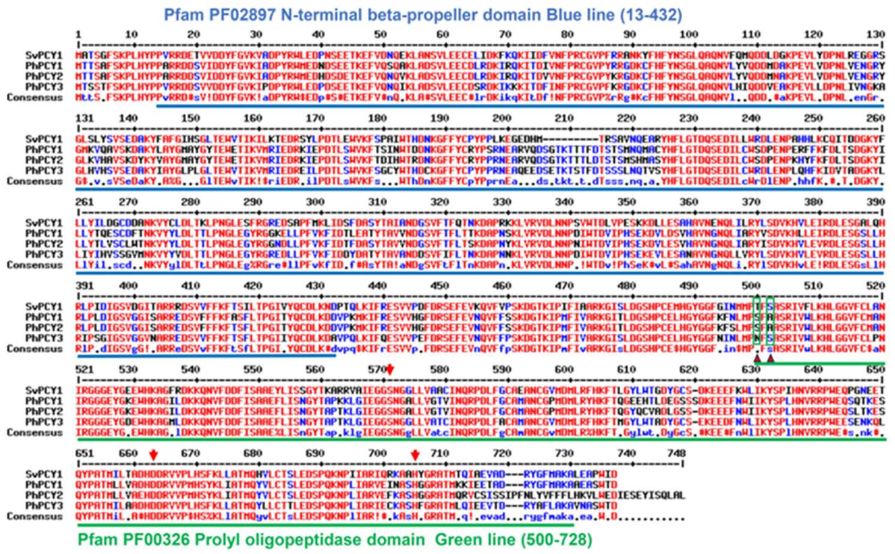

Supplemental Figure 11. Amino acid sequence alignment and mutation site analysis of *PhPCY1/2/3* and *SvPCY1*.

The PCYs have beta-propeller domain (13-432) at the N-terminal (Pfam PF02897) under the blue line and prolyl oligopeptidase domain (500-728) at the C-terminal (Pfam PF00326) under the green line.The conserved triad amino acids are marked with red arrows (S562, D653, H695 in SvPCY1 and S571, D662, H704 in PhPCY3). Meanwhile, N500 and S502 sites were highlighted with green text box and red triagnle.

Substrate Name	Substrate PreCPs [14-35/36]																						
	14	15	16	17	18	19	20	21	22	23	24	25	26	27	28	29	30	31	32	33	34	35	36
PreHB [14-35]	I	F	G	G	L	P	P	P	S	Q	E	L	I	N	G	D	D	I	S	L	M	V	
Residue Sites (AA)	V84	G401	G402							M498	R505	G570	N594	R707		S502	N500	K414	N104		Q712		
																A503							
																S504							
PrePE [14-36]	V	I	F	G	P	P	L	G	P	S	Q	V	M	I	N	G	D	D	I	S	V	M	V
Residue Sites (AA)							K250			E356	N494			N594	M498		H488	N594		R719	S502		N500
										R369	N496						G570	R719			S504		
																					R505		
PrePF [14-35]	L	S	G	G	Y	L	P	P	S	Q	E	L	I	N	G	D	D	I	S	V	M	V	
Amino Acid (AA)		R88			H133	H133					N104		K414			N500	H488	N594	S502		N500	N500	
		S136									T709					R505	G570	R719	S504				
		V137									Q712												
PrePG [14-35]	F	S	F	G	P	L	A	P	S	Q	V	L	I	N	G	D	D	I	S	V	M	V	
Residue Sites (AA)	N594	R719		N500	K414		K414		G401	V84			Q103	R81		G706			N500			N496	
	R719													R707		R707							
														T709		Q712							
														Q712									

Supplemental Figure 12. Key catalytic sites identified via molecular docking.

Hydrogen-bonding interactions in 4 Å were analyzed between the residue sites of PhPCY3 and the substrate PreCPs [14-35/36], and the residues binding position at the substrate were also identified and recorded. The core peptide regions have the pink background color and the follower peptide at C termial have the blue background color, while the common residue site N500 and S502 has a red background.

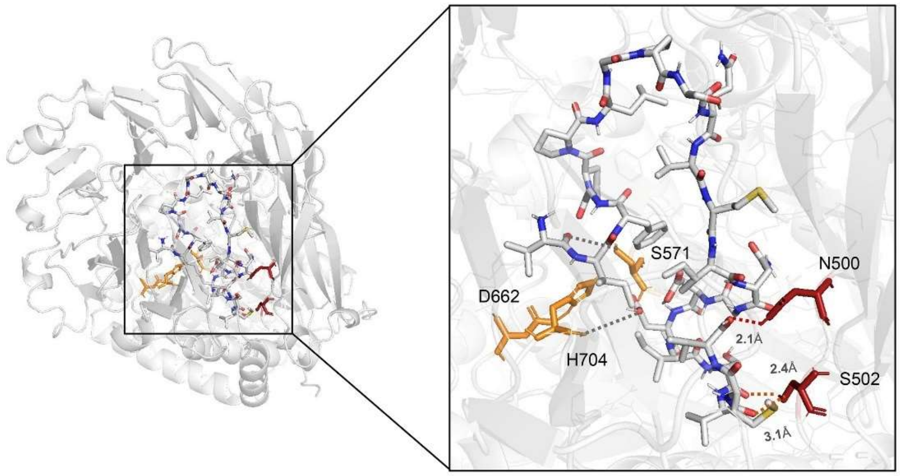

Substrate PE [14-36] molecular docking with PhPCY3

Supplemental Figure 13. Molecular docking the substrate PE [14-36] with PhPCY3 within 4Å and triad amino acids.

Hydrogen-bonding interactions between the residue sites (N500 and S502) of PhPCY3 and the substrate PE [14-36] (color the sticks with heteroatom) within 4 Å were highlighted by dark red dashed lines. The triad amino acids (S571, D662 and H704) are highlighted with orange sticks in the molecular docking. The triad residues nearest to oxygen atom in the substrate are highlighted by the gray dashed lines.

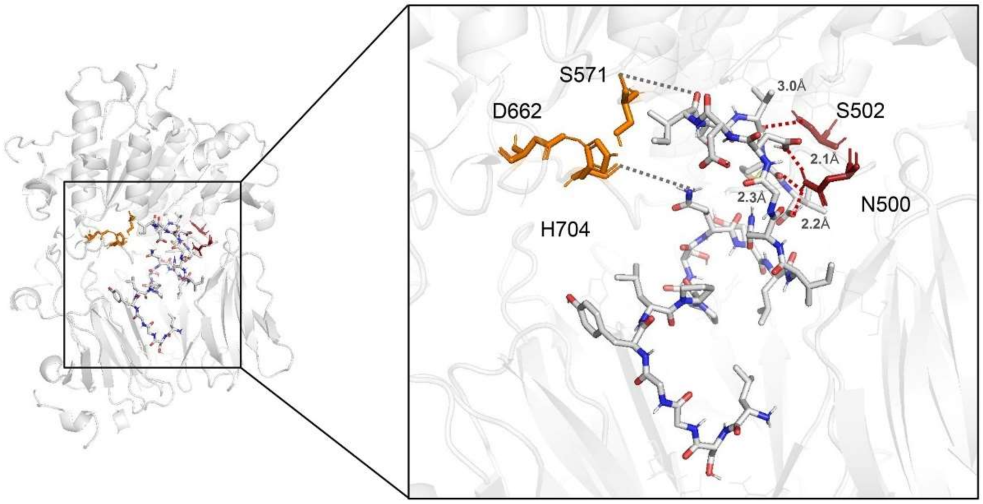

Substrate PF [14-35] molecular docking with PhPCY3

Supplemental Figure 14. Molecular docking the substrate PF [14-35] with PhPCY3 within 4Å and triad amino acids.

Hydrogen-bonding interactions between the residue sites (N500 and S502) of PhPCY3 and the substrate PF [14-35] (color the sticks with heteroatom) within 4 Å were highlighted by dark red dashed lines. The triad amino acids (S571, D662 and H704) are highlighted with orange sticks in the molecular docking. The triad residues nearest to oxygen atom in the substrate are highlighted by the gray dashed lines.

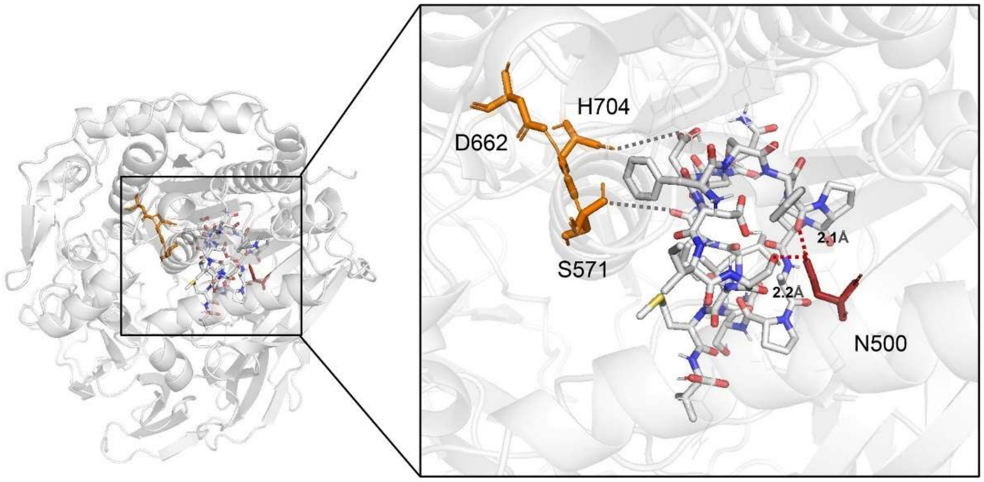

Substrate PG [14-35] molecular docking with PhPCY3

Supplemental Figure 15. Molecular docking the substrate PG [14-35] with PhPCY3 within 4Å and triad amino acids.

Hydrogen-bonding interactions between the residue site (N500) of PhPCY3 and the substrate PG [14-35] (color the sticks with heteroatom) within 4 Å were highlighted by dark red dashed lines. The triad amino acids (S571, D662 and H704) are highlighted with orange sticks in the molecular docking. The triad residues nearest to oxygen atom in the substrate are highlighted by the gray dashed lines.

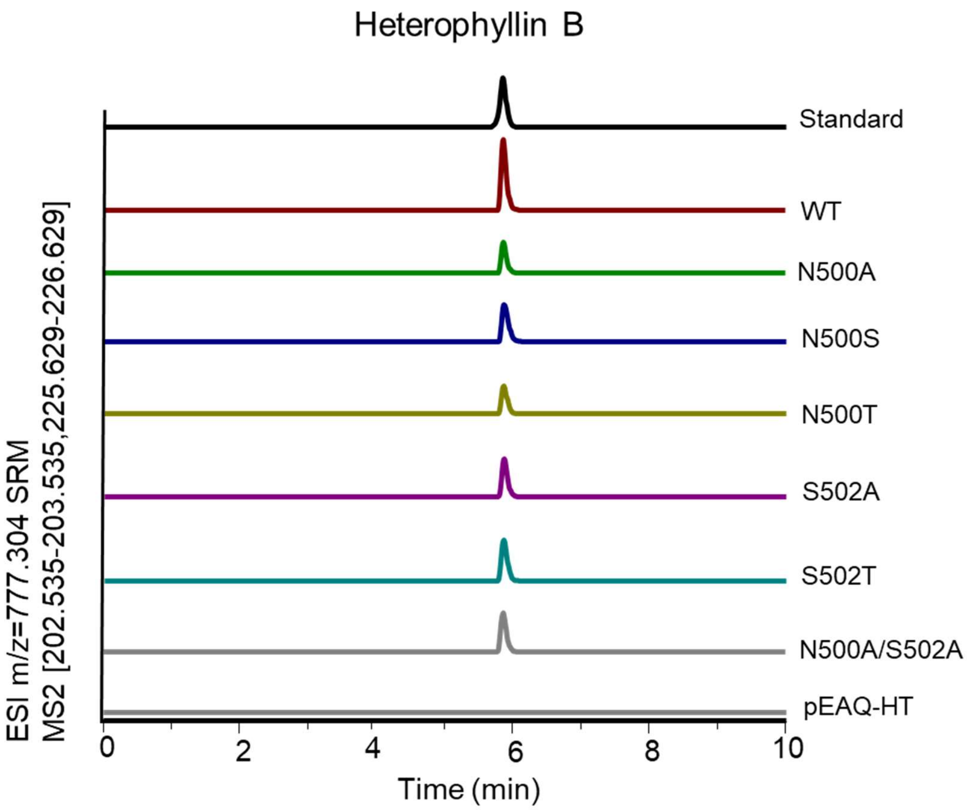

Supplemental Figure 16. The ion chromatograms (SRM) of heterologous expression mutations of *PhPCY3* with *PhPreHB* in *N. benthamiana* leaves. The ion chromatograms (SRM) of *N. benthamiana* leaf samples which have expressed *PhPreHB* with *PhPCY3* (WT) (or *PhPCY3* mutants at the N500 and S502 sites, including N500A, N500S, N500T, S502A, S502T, N500A/S502A) were detected using LC-MS in the selected reaction monitoring (SRM) mode, respectively.

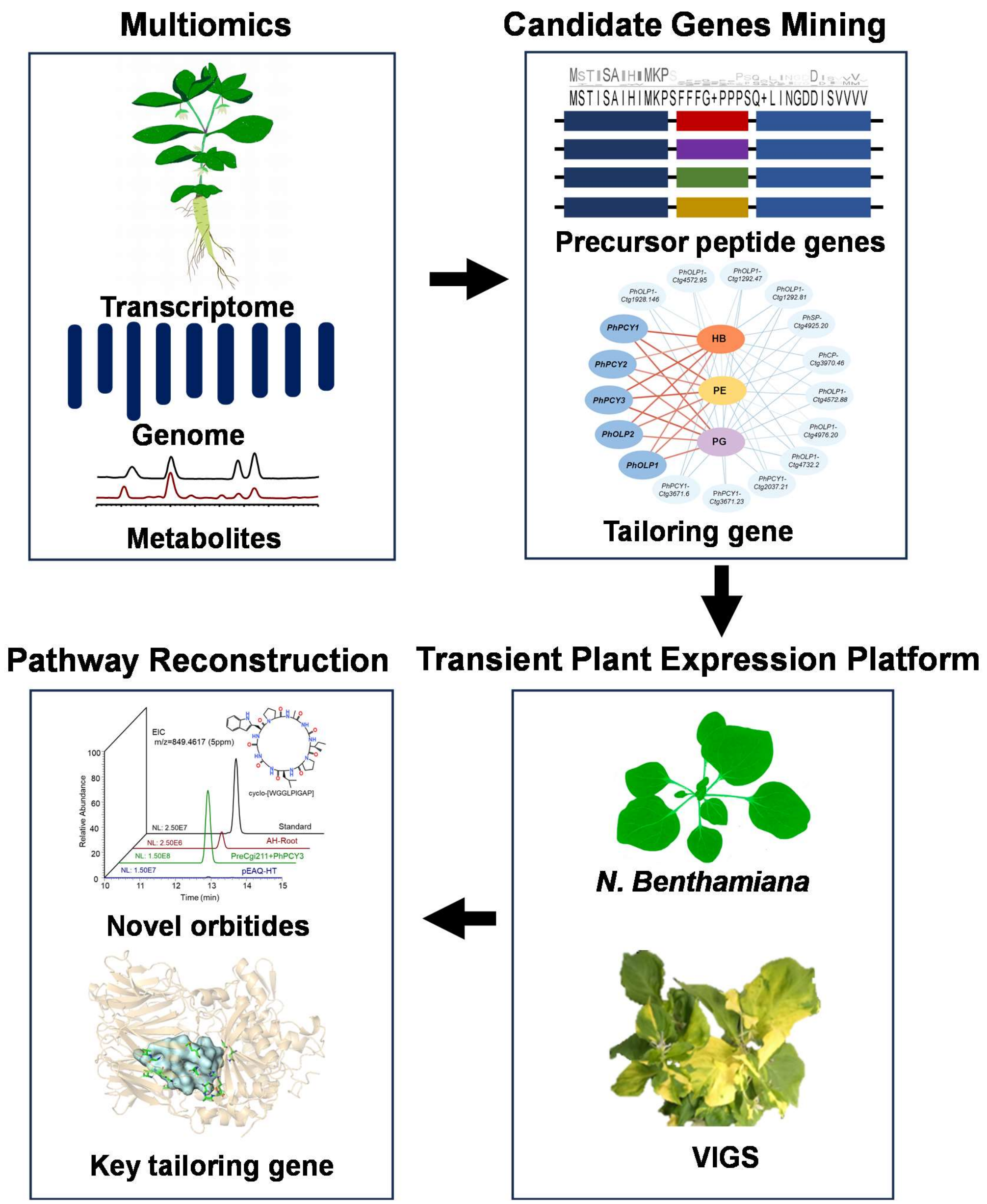

Supplemental Figure 17. Reverse genetics strategy for mining novel orbitides via a transient expression platform. Multiple omics data have been used to explore the candidate genes in the orbitide biosynthesis pathway. Their functions *in vivo* were elucidated using the heterologous expression system in *N. benthamiana* and the virus-induced gene silencing (VIGS) system in *P. heterophylla*. Novel orbitides were discovered through pathway reconstruction with the key tailoring gene.

Supplemental Table 1. Primers for all the candidate genes with pEAQ-HT and pMAL-c5x vectors. The primers were designed by CE Design V 1.04 software, the pEAQ-HT vector was linearized at *Age*I and *Xho*I sites and the pMAL-c5x vector was linearized at *Bam*HI and *Eco*RI sites.

Full Names	Primer Names	Primer Sequences (5'-3')
PhPreHB	pEAQ-PreHB-F	caaattcgcgaccggtATGTCTACTATTTCAGCCATCCACATTATGAAG
	pEAQ-PreHB-R	agttaaaggcctcgagTTACACCATGAGGGAAATATCATCTCCATTTATCAG
PhPrePE	pEAQ-PrePE-F	caaattcgcgaccggtATGTCTACTATTTCAGCCTTCCACATTATGAAG
	pEAQ-PrePE-R	agttaaaggcctcgagTTACACCATGACGGAAATATCATCCCCAT
PhPrePF	pEAQ-PrePF-F	caaattcgcgaccggtATGTCTACTATTTCAGCCATCCACATTATGAAG
	pEAQ-PrePF-R	agttaaaggcctcgagTTATACCATGACGGAAATATCATCTCCATTTATCAGC
PhPrePG	pEAQ-PrePG-F	caaattcgcgaccggtATGTCTACTATTTCAGCCATCCACATTATGAAG
	pEAQ-PrePG-R	agttaaaggcctcgagTTAGACCATGACGGAAATATCATCCCCATT
PhOLP1-	pEAQ-OLP1-F	caaattcgcgaccggtATGGAGAAATCTAGCAAGAATATGAACAAAATCATGG
Ctg4232.8	pEAQ-OLP1-R	agttaaaggcctcgagCTAACATATTGGTAAATCTTCTGGTGGAGACT
PhOLP1-	pEAQ-OLP2-F	caaattcgcgaccggtATGGCATTCAAAAAAACACTAGTACAAAGATTATTCAAC
Ctg5689.43	pEAQ-OLP2-R	agttaaaggcctcgagCTAATTAACCTTATGGTAATGATCATACTTGATCAACCTAG
PhPCY1-	pEAQ-PCY1-F	caaattcgcgaccggtATGACTACCTCCGCCTTCTCCA
Ctg883.47	pEAQ-PCY1-R	agttaaaggcctcgagTTAATCCGTCCAAGAGGCTTCAGCT
PhPCY1-	pEAQ-PCY2-F	caaattcgcgaccggtATGACTACCTCCGCCTTCTCCA
Ctg883.48	pEAQ-PCY2-R	agttaaaggcctcgagTCATAATGCCAGTTGAGAAATATACTCACTCTCTATGT
PhPCY1-	pEAQ-PCY3-F	caaattcgcgaccggtATGACTTCCTCCACCTTCTCCAAAC
Ctg883.50	pEAQ-PCY3-R	agttaaaggcctcgagTTAATCTGTCCAAGAGGCGTTGACCG
PhPCY1-	pEAQ-PCY4-F	caaattcgcgaccggtATGAGCACGCCTCTATTACGATTACG
Ctg2037.21	pEAQ-PCY4-R	agttaaaggcctcgagTTAGTCAATCCAAGAAGCATTCACCACT
PhPCY1-	pEAQ-PCY5-F	caaattcgcgaccggtATGATGTCTTCATCTGTCTCCGCC
Ctg3671.23	pEAQ-PCY5-R	agttaaaggcctcgagTTAATCAGTCCACGGGGAATTCACGAC
PhPCY1-	pEAQ-PCY6-F	caaattcgcgaccggtATGGGCGTTGAAGTGTCGTTG
Ctg3671.6	pEAQ-PCY6-R	agttaaaggcctcgagTTAGTTGATCCATGACACATCCATCATCTTAGC
pMAL-c5x-	pMAL-PCY3-F	CGAGATATCGTCGACGGATCCATGACTTCCTCCACCTTCTCCA
PhPCY3-	pMAL-PCY3-R	TTAATTACCTGCAGGGAATTCTTAATCTGTCCAAGAGGCGTTGA

Supplemental Table 2. Primers for the candidate genes with pTRV2 vector. VIGS Tool in Solanaceae Genomics Network (https://vigs.solgenomics.net) has been used to analyze the target sequences. The primers were designed by CE Design V 1.04 software and the vector was linearized at *Eco*RI and *Bam*HI sites.

Primer Names	Primer Sequences (5'-3')
pTRV2-*PhPreHB-F*	gtgagtaaggttaccgaattcATGTCTACTATTTCAGCCATCCACA
pTRV2-*PhPreHB-R*	cgtgagctcggtaccggatccTTACACCATGAGGGAAATATCATCTC
pTRV2-*PhPrePE-F*	gtgagtaaggttaccgaattcATGTCTACTATTTCAGCCTTCCACAT
pTRV2-*PhPrePE-R*	cgtgagctcggtaccggatccTTACACCATGACGGAAATATCATCC
pTRV2-*PhPrePG-F*	gtgagtaaggttaccgaattcATGTCTACTATTTCAGCCATCCACA
pTRV2-*PhPrePG-R*	cgtgagctcggtaccggatccTTAGACCATGACGGAAATATCATCC
pTRV2-*PhChlH 1-F*	gtgagtaaggttaccgaattcTGAACAGGCCAATACCCTGG
pTRV2-*PhChlH 1-R*	cgtgagctcggtaccggatccAAGTAGTGGCTCACATCAGTTAAAGAA
pTRV2-*PhPCY3-F*	gtgagtaaggttaccgaattcAAAGTACTCGCCGCTCCATAAC
pTRV2-*PhPCY3-R*	cgtgagctcggtaccggatccGCGTTGACCGCCTTTGCC

Supplemental Table 3. qRT-PCR primers for precursor peptide genes and *PhPCY3*. The primers were designed by Primer Premier 5.0 software.

Primer Names	Primer Sequences (5'-3')
Phβ-Actin-F	CTGTATTTACGCTCAGGTGG
Phβ-Actin-R	CATTGTGCTCAGTGGTGG
PhPreHB-F	CATCCACATTATGAAGCCGAGTA
PhPreHB-R	CCCAACGGTGACGGACAT
PhPrePE-F	ATGTCTACTATTTCAGCCTTCCAC
PhPrePE-R	TCATCCCCATTTATCATCACC
PhPrePG-F	CAGCCATTCACATTATGAAGCC
PhPrePG-R	CATCCCCATTTATCAGCACC
PhPCY3-F	TGGCACAAGGCAGGAATG
PhPCY3-R	CGTTATGGAGCGGCGAGTA

Supplemental Table 4. Primers for site-directed mutation at N500 and S502 site of PhPCY3. The primers were designed by CE Design V 1.04 software and the pEAQ-HT vector was linearized at *Age*I and *Xho*I sites.

Primer Names	Primer Sequences (5'-3')
PhPCY3(WT)-F1	ctgcccaaattcgcgaccggtATGACTTCCTCCACCTTCTCCA
PhPCY3(WT)-R2	accagagttaaaggcctcgagTTAATCTGTCCAAGAGGCGTTGA
PhPCY3-N500A-F2	GTTTAACATAAACTTGATGCCAGCTTTTTCTGCTAGTCGTATAGTG
PhPCY3-N500A-R1	CACTATACGACTAGCAGAAAAAGCTGGCATCAAGTTTATGTTAAAC
PhPCY3-N500S-F2	GTTTAACATAAACTTGATGCCATCTTTTTCTGCTAGTCGTATAGTG
PhPCY3-N500S-R1	CACTATACGACTAGCAGAAAAAGATGGCATCAAGTTTATGTTAAAC
PhPCY3-N500T-F2	GTTTAACATAAACTTGATGCCAACTTTTTCTGCTAGTCGTATAGTG
PhPCY3-N500T-R1	CACTATACGACTAGCAGAAAAAGTTGGCATCAAGTTTATGTTAAAC
PhPCY3-S502A-F2	ATAAACTTGATGCCAAATTTTGCTGCTAGTCGTATAGTGTGGCT
PhPCY3-S502A-R1	AGCCACACTATACGACTAGCAGCAAAATTTGGCATCAAGTTTAT
PhPCY3-S502T-F2	ATAAACTTGATGCCAAATTTTACTGCTAGTCGTATAGTGTGGCT
PhPCY3-S502T-R1	AGCCACACTATACGACTAGCAGTAAAATTTGGCATCAAGTTTAT
PhPCY3-N500A&S502A-F2	GTTTAACATAAACTTGATGCCAGCTTTTGCTGCTAGTCGTATAGTGTGGCTG
PhPCY3-N500A&S502A-R1	CAGCCACACTATACGACTAGCAGCAAAAGCTGGCATCAAGTTTATGTTAAAC

Supplemental Method 1. Supplementary methods for LC-MS analysis.

(a) HPLC/TSQ-MS (Thermo Scientific™)

Detection Method: LC-MS/MS---SRM

Instrument: UltiMate 3000 HPLC + TSQ Quantum Access Max

Mode: Negative

Column: XBridge BEH C18 Column (Waters, 130 Å, 2.5 μm, 2.1 mm × 100 mm)

Mobile phase: Solvent A: Acetonitrile;

Solvent B: Water with 0.1% (v/v) formic acid

Flow rate: 0.3 mL/min

Injection volume: 5.0 μl

Mobile phase elution gradient (See the table below for details)

Time	Solvent A (%)	Solvent B (%)
0	10	90
1.0	10	90
4.5	90	10
4.5	90	10
4.6	10	90
6.0	10	90
6.0	Stop Run	

Particle fragment information [M-H]-：

Parent M	Product M	CE	T lens
777.304	203.035	48	132
777.304	226.129	49	132
783.295	647.456	36	99
783.295	753.550	26	99
815.291	618.395	33	104
815.291	785.563	26	104
876.329	153.061	58	145
876.329	300.063	54	145

Mass spectrometer parameters:

Instrument	TSQ Quantum Access
Mode	ESI positive
Vaporizer Temperature	320°C
Capillary Temperature	340°C
Spray Voltage	3600 V
Sheath Gas Pressure	35 L/h
Aux Gas Pressure	10 L/h
Spare Gas Pressure	0.0 L/h
Scan Width	1.0 m/z
Scan Time	0.08

(b) HPLC/ Q Exactive HFX MS (Thermo Scientific™)

Detection Method: Full scan/ddMS2 mode

Instrument: UltiMate 3000 HPLC + Q Exactive™ HFX Mass Spectrometer

Mode: Negative and Positive

Column: ACQUITY UPLC BEH C18 VanGuard Pre-column

(Waters, 130 Å, 1.7 µm, 2.1 mm × 5 mm)

Mobile phase: Solvent A: Acetonitrile

Solvent B: Water with 0.1% (v/v) formic acid

Flow rate: 0.3 mL/min

Injection volume: 5.0 µL

Mobile phase elution gradient (See the table below for details)

Time	Solvent A (%)	Solvent B (%)
0	10	90
2.0	10	90
15.0	95	5
20.0	95	5
20.0	95	5
25.0	10	90
25.0	Stop Run	

Mass spectrometer parameters:

Instrument	Q Exactive™ HFX MS
Ion Source	HESI
Vaporizer Temperature	320°C
Capillary Temperature (+ or + -)	340°C
Capillary Temperature (-)	350°C
Spray Voltage (+)	3000 V
Spray Voltage (-)	3500 V
Sheath Gas Pressure (+ or + -)	35 L/h
Sheath Gas Pressure (-)	35 L/h
Aux Gas Pressure (+ or + -)	10 L/h
Aux Gas Pressure (-)	10 L/h
Spare Gas Pressure (+ or + -)	0.0 L/h
Spare Gas Pressure (-)	0.0 L/h
Probe Heater Temp (+ or + -)	340°C
Probe Heater Temp (-)	375°C
S-Lens RF Level	50.00

Supplemental Note 1. The CDS sequence of precursor peptide genes in *P. heterophylla*. The fasta file described the CDS sequences of the precursor peptide genes in different cultivated *P. heterophylla*.

Supplemental Note 2. Sequences of the candidate genes that may be involved in orbitide biosynthesis. The word file described the CDS sequences of PhSP, PhCP, PhOLPs and PhPCYs.

>PhPCY1-Ctg883.50 **(PhPCY3)**

ATGACTTCCTCCACCTTCTCCAAACCCTTGCACTACCCGCCTGTCCGCCGCGACGAT
TCCGTCGTCGACGACTATTTCGGTGTCAAAATCCCCGACCCATACCGTTGGCTGGAG
GATCCAGATTCGGAAGAGACGAAAGAGTTTGTACAGAATCAAATAAAGCTTGCGGATT
CAGTGCTCGAAGAGTGCGATTCAAGAGACAAAATCAAGAAGAAAATCACTGATTTTAT
CAATTTTCCGCGTTGTGGTGTTCCATTTAAGCGCGGTGATAAGTGTTTTCATTTTTATA
ATTCTGGCCTTCAGGCGCAAAATGTGCTTCATATTCAGGATGATGTGGAAGCAAAGC
CAGAGGTGCTACTTGATCCTAACCTTATTGTGAACGGAAAAGCTGGTTTGCACGTGC
ATTCTGTAAGCGAGGACGCCAAATATATTGCATACGGTCTGCCTTTAGGTTTGACTGA
ATGGGTGACTATCAAAGTAATGAGAATTGAAGACCGAGAAATTTTACCAGACACTTTAT
CGTGGGTGAAGTTTAGTGGTTGTTATTGGACCCATGACTGTAAAGGATTTTTCTATTG
CCCGTATCCACCCCGCAATGAAGCACAAGAAGAAGATTCTGAAACTAAGACTTCTAC

CTTCGATACTAGTTCTAGCTTGAATCAGACGGTAAGCTATCATTTTCTCGGCACGGAT
CAGTCTGAAGATATTCTGTGCTGGAGAGACCTTGAGAACCCTTTACAACACTTTAAGA
TAGATGTAACTGCTGACGGAAAGTATCTTCTTATCTATATTCATGTGAGCTCTGGTGTG
ATGAACAAAGTATACTATGTGGATTTAACAACGCTGCCTAATGGGCTTGAAGGTTATC
GTGGACGAGAAGACTTGCTTCCTTTCGTAAAGTTTATTGATGATTATGATGCAACGTAT
ACAGCCGTTGCTAATGATGACTCTGTGTTTATTTTCCTAACTAACAAGGACGCTCCCA
ATAATAAGTTGGTTCGTGTTGATTTGAATAATCCCGACATATGGACTGATGTGATTCCA
CATTCTGAGAAGGAAGTGCTTGAATCAGCAAATGCTGTTAATGGAAATCAGCTTCTTG
TCCGTTACCTAAGCGATGTCAAGCATGTTCTTGAAGTTAGAGATCTTGAGAGTGGCTC
CTTGCTGCATCGCATACCGTCAGGCATCGGATCTGTTGGTGGAGTTAATGCTCGACG
GGAAGATAGTGTCGTGTTTTTTAAATTCACAAGCTTCCTGACTCCTGGCATTATTTACC
AATGTGATTTGAAAGATGCTGTTCCACAGCTGAAGATTTTTCAAGAAAGTGTTGTCCC
TGAATTTGACCGTTCTGAGTTTGAGGTTAATCAGGTTTTTTTCCCAGCAAAGATGGT
ACAAAGATACCAATGTTCATAGTGGCGAGAAAGGGAATTTCTTTGGACGGATCACAC
CCATGTGAACTGCATGGTTATGGCGGGTTTAACATAAACTTGATGCCAAATTTTTCTG
CTAGTCGTATAGTGTGGCTGAAACACCTTGGTGGAGTCTTCTGCTTGCCTAATATCCG
AGGTGGTGGTGAATATGGAGATGAATGGCACAAGGCAGGAATGCTTGATAAGAAGCA
GAATGTTTTTGATGACTTCATCTCTGCAGCTGAGTTTCTTATTTCTAATGGCTATACCG
CACCTACAAAATTGGGTATTGAAGGTGGAAGTAACGGTGGCCTTCTTGTTGCTACCT
GTATTAATCAGAGACCAGACCTTTTTGCTTGCGCTATGGCAAACTGTGGTGTAATGGA
CATGCTTCGATTCCATAAATTTACCATGGGTTATCTTTGGACGGCGGATTATGGTTGCT
CTGAAAAAGAGGAAGACTTCAACTGGCTTATAAAGTACTCGCCGCTCCATAACGTGA
GGCGGCCATGGGAGGAATCAGAGAATAAACAGTTACAGTACCCTGCTACAATGATATT
AGCAGCTGATCATGATGATCGTGTCGTGCCTCTGCACTCCTTTAAACTGTTGGCTACT
ATGCAGTACCTTCTTTGCACAACTTTGGAGGACAGCCCGCAGAAGAATCCGTTAATT
GCTCGAATTGAGTGCAAAGCTTCACACTTTGGACGTGCGACTATGTTGCAGATTGAG
GAAGTTACAGATCGGTATGCCTTTCTGGCAAAGGCGGTCAACGCCTCTTGGACAGAT
TAA
